# Controlled Human Infection of Healthy Adults With Lyophilized *Neisseria lactamica* Induces Asymptomatic, Immunogenic Nasopharyngeal Carriage in the United Kingdom and Mali

**DOI:** 10.1093/ofid/ofaf809

**Published:** 2026-01-07

**Authors:** D F Gbesemete, F Haidara, J R Laver, M Ibrahim, J MacLennan, A P Dale, A R Gorringe, Y Traore, F Diallo, H Badji, A Traore, U Onwuchekwa, E Jones, C Webb, J Guy, A A Theodosiou, S N Faust, S O Sow, R S Heyderman, M D Tapia, R C Read

**Affiliations:** Controlled Human Infection Group, Clinical and Experimental Sciences, Faculty of Medicine, University of Southampton, Southampton, UK; NIHR Southampton Biomedical Research Centre and NIHR Southampton Clinical Research Facility, University Hospital Southampton, Southampton, UK; Centre pour les Vaccins en Développement - Mali, Bamako, Mali; Controlled Human Infection Group, Clinical and Experimental Sciences, Faculty of Medicine, University of Southampton, Southampton, UK; Controlled Human Infection Group, Clinical and Experimental Sciences, Faculty of Medicine, University of Southampton, Southampton, UK; Department of Zoology, University of Oxford, Oxford, UK; Controlled Human Infection Group, Clinical and Experimental Sciences, Faculty of Medicine, University of Southampton, Southampton, UK; United Kingdom Health Security Agency, Porton Down, Salisbury, UK; Centre pour les Vaccins en Développement - Mali, Bamako, Mali; Centre pour les Vaccins en Développement - Mali, Bamako, Mali; Centre pour les Vaccins en Développement - Mali, Bamako, Mali; Centre pour les Vaccins en Développement - Mali, Bamako, Mali; Centre pour les Vaccins en Développement - Mali, Bamako, Mali; Controlled Human Infection Group, Clinical and Experimental Sciences, Faculty of Medicine, University of Southampton, Southampton, UK; Controlled Human Infection Group, Clinical and Experimental Sciences, Faculty of Medicine, University of Southampton, Southampton, UK; Controlled Human Infection Group, Clinical and Experimental Sciences, Faculty of Medicine, University of Southampton, Southampton, UK; Controlled Human Infection Group, Clinical and Experimental Sciences, Faculty of Medicine, University of Southampton, Southampton, UK; NIHR Southampton Biomedical Research Centre and NIHR Southampton Clinical Research Facility, University Hospital Southampton, Southampton, UK; Faculty of Medicine and Institute for Life Sciences, University of Southampton, Southampton, UK; Centre pour les Vaccins en Développement - Mali, Bamako, Mali; NIHR Global Health Research Unit on Mucosal Pathogens, Division of Infection & Immunity, University College London, London, UK; Center for Vaccine Development and Global Health, University of Maryland, School of Medicine, Baltimore, Maryland, USA; Controlled Human Infection Group, Clinical and Experimental Sciences, Faculty of Medicine, University of Southampton, Southampton, UK; NIHR Southampton Biomedical Research Centre and NIHR Southampton Clinical Research Facility, University Hospital Southampton, Southampton, UK; Faculty of Medicine and Institute for Life Sciences, University of Southampton, Southampton, UK

**Keywords:** African meningitis belt, controlled human infection, lyophilization, *Neisseria lactamica*, *Neisseria meningitidis*

## Abstract

**Background:**

Carriage of *Neisseria lactamica* (Nlac), a harmless nasopharyngeal commensal, correlates inversely with carriage of *Neisseria meningitidis* (Nmen), a common cause of meningitis and sepsis outbreaks in sub-Saharan Africa. Nasally administered lyophilized Nlac (LyoNlac) might interrupt carriage and transmission of Nmen in sub-Saharan settings without requirement of a cold chain, but whether LyoNlac can establish colonization is undetermined.

**Methods:**

Healthy adult volunteers aged 18–45 years were inoculated intranasally with 10^4^–10^7^ colony forming units (CFU) of reconstituted, lyophilized Nlac strain Y92-1009 (LyoNlac) in 2 dose-ranging controlled human infection studies conducted in the United Kingdom and Mali. Safety was measured as a primary objective. Secondary objectives included the dose achieving ≥70% colonization rates for each setting, colonization kinetics, and serological responses. Both trials were registered with ClinicalTrials.gov (United Kingdom: NCT04135053, Mali: NCT04665791) and are complete.

**Results:**

Intranasal inoculation with LyoNlac was well tolerated with no significant safety concerns. In the United Kingdom, 10^5^ CFU yielded 100% colonization (n = 10/10) while in Mali, 10^7^ CFU achieved 65% colonization (n = 13/20). An increase in Nlac- and Nmen-specific IgG from pre-challenge to day 28 post-challenge was observed in colonized participants—median fold-change [interquartile range] United Kingdom: Nlac 2.24 [1.37–4.24], Nmen 1.39 [1.20–3.70] and Mali: Nlac 1.31 [1.04–1.94], Nmen 1.32 [0.99–1.73]. No significant seroconversion occurred in non-colonized participants.

**Conclusions:**

Intranasal inoculation with LyoNlac was safe and induced immunogenic nasopharyngeal colonization in healthy adults in the United Kingdom and Mali. Future clinical trials to determine whether LyoNlac reduces meningococcal carriage and transmission in the meningitis belt are warranted.

## Teaser

Controlled human infection with a lyophilized preparation of the harmless commensal *Neisseria lactamica* induces safe nasopharyngeal colonization in healthy adults in the United Kingdom and Mali, which induces *Neisseria lactamica*-specific and cross-reactive *Neisseria meningitidis*-specific immune responses.

The meningitis belt is a region of sub-Saharan Africa with a high incidence of meningococcal meningitis [1]. Outbreaks typically occur during the dry season and disappear with the rains, with large epidemics occurring every 5–12 years [2, 3]. Multiple serogroups of *Neisseria meningitidis* (Nmen) are implicated, necessitating either single-component or multi-component vaccines for control. Mass vaccination with the Nmen serogroup A conjugate vaccine, MenAfriVac, and its subsequent introduction into the Expanded Programme on Immunisation (EPI), resulted in a dramatic decline in Nmen A disease and pharyngeal carriage [4, 5], but outbreaks continue to occur with an increase in carriage of, and disease caused by other serogroups such as C, X, and W [5–7]. A recently developed pentavalent ACYWX conjugate vaccine is being introduced into routine childhood immunization schedules across the meningitis belt and in mass vaccination campaigns in the highest risk areas [8]. The impact of this vaccine on meningococcal carriage and disease will be evaluated post-rollout.

Vaccination campaigns are triggered when the incidence of disease crosses a pre-defined epidemic threshold [9]. These are logistically complex and depend upon rapid serogroup identification, adequate vaccine supply, storage and transport infrastructure, a robust cold chain, and trained healthcare staff. These constraints cause delays in response times [10].

Nasopharyngeal colonization with Nmen is prerequisite to the development of meningococcal disease [11], but the majority of colonization events remain asymptomatic, with a minority progressing to invasion and disease (most commonly meningitis, sepsis, or both). Colonization rates vary by location and age. In high income countries, approximately 10% of the population are colonized, increasing from a very low prevalence in infants, to a peak of 24% in adolescence [12]. Across the meningitis belt, overall colonization is lower at approximately 4%, but with significant variation by age, season, and country [13]. During epidemics, colonization prevalence can increase up to 30-fold, suggesting that an increase in asymptomatic colonization drives outbreaks [14]. In addition to providing direct protection from disease, capsular polysaccharide-conjugate vaccines protect against asymptomatic colonization and therefore onward transmission. However, this effect is serogroup specific [4, 15–17]. It follows therefore that the development of an intervention capable of rapidly preventing asymptomatic meningococcal colonization could decelerate or even terminate an outbreak. In support of such an approach are data demonstrating that during a sub-Saharan outbreak, village-wide administration of ciprofloxacin to reduce colonization and therefore person-to-person spread reduced the subsequent attack rate within that village [18].

*Neisseria lactamica* (Nlac) is a common commensal of the human pharynx during childhood [19–21]. It is non-capsulate and extremely rarely associated with disease [22]. Colonization with Nlac is inversely associated with Nmen colonization, both in high income countries [20] and in the meningitis belt [13]. Mathematical modeling suggests that Nmen acquisition is inhibited for several years following natural Nlac colonization which peaks at 2.4 years [23]. Furthermore, in a carriage survey conducted during a meningococcal outbreak, geographical areas with higher rates of colonization with Nlac were found to have a reduced incidence of meningococcal disease [24].

We previously demonstrated that experimental nasopharyngeal Nlac colonization of humans via intra-nasal inoculation significantly reduced colonization with Nmen by both direct displacement and prevention of new meningococcal acquisition events. This effect was evident within 2 weeks post-inoculation, with the effect persisting until at least 16 weeks, and was not restricted to a particular meningococcal clonal complex [25]. The mechanism underpinning this effect could be direct competition for niche occupation or induction of cross-reactive adaptive immunity. In support of the latter hypothesis, we demonstrated that Nlac colonization induces cross-reactive adaptive immune responses, including antibody with anti-meningococcal opsonic activity, and cross-reactive immunological memory [26, 27]. We have observed no safety signals having inoculated more than 350 volunteers, and colonization is stable and long-lasting [25–29].

For Nlac to be used as a live bacterial product in a sub-Saharan outbreak, it would require a formulation that could be deployed conveniently and without a cold chain or medical expertise. We therefore developed a lyophilized formulation of Nlac strain Y92-1009 (LyoNlac) suitable for long-term storage and distribution beyond a cold chain, which could then be reconstituted for use as a nasal inoculum. Given the inherent differences in host immunity between the United Kingdom and sub-Saharan Africa, it was considered vital to demonstrate the colonization potential and immunogenicity of Nlac in a population within the meningitis belt as well as in the United Kingdom. Therefore, to test the hypothesis that inoculation with reconstituted LyoNlac would safely and reliably induce immunogenic nasopharyngeal Nlac colonization, we performed 2 dose-escalation controlled human infection studies. The first study was conducted in the United Kingdom, and the second in Mali, within the meningitis belt [30]. The objectives of these studies were to determine the optimum dose of LyoNlac required to induce nasopharyngeal colonization in healthy adults in each setting and to demonstrate the safety and immunogenicity of experimentally induced colonization.

## METHODS

### Challenge Agent Production

LyoNlac was developed from Nlac strain Y92-1009, ST-3493, clonal complex 613, originally produced according to Good Manufacturing Practice (GMP) principles by UK Health Security Agency at Porton Down, Salisbury, and stored as frozen bacterial stocks. Subsequently, a GMP-like process was used in a dedicated facility at the University of Southampton, with highly structured standard operating procedures and dedicated personnel and equipment, consistent with current recommendations and best practice [31]. Briefly, log phase Nlac, grown in Tryptone Soya Broth supplemented with 0.2% yeast extract (TSB), were washed in PBS, resuspended in a soya-based lyophilization excipient, snap-frozen, lyophilized, and then vacuum sealed in ampoules as described previously [29]. The same batch of LyoNlac was used in both studies (batch number: LyoNlac01-190221).

Previous analysis of LyoNlac viability following storage at different temperature conditions showed ampoules remained above minimum viable product (MVP) levels (≥10^7^ colony forming unit [CFU] per ampoule) for a minimum of approximately 3 months (Supplementary materials and Supplementary Figure 1). These data imply that LyoNlac might be a stable formulation in which to store an infective inoculum beyond a reliable cold chain. Viable counts and purity checks were performed (1) immediately following production, (2) prior to study start, (3) following each inoculation, and (4) following transportation to Mali.

### Patient Consent Statement

The first study was conducted at University Hospital Southampton Clinical Research Facility, United Kingdom, between May 2019 and March 2020 (UK study). This study was registered with ClinicalTrials.gov (NCT04135053), with approval from South-Central Hampshire B Research Ethics Committee (Ref 18/SC/0420). Following review of the safety and colonization data, the second study was conducted at the Centre pour le Développement des Vaccins - Mali, Bamako, Mali, between March and June 2021 (Mali study). It was registered with ClinicalTrials.gov (NCT04665791) with approval from the University of Maryland, Baltimore Institutional Review Board (HM-HP-0093831-2), and the ethics committee of the Faculté de Médecine, Pharmacie et Odonto-Stomatologie, Mali (2020/291/CE/FMOS/FAPH). Both studies were conducted in accordance with the Declaration of Helsinki (1996) and the International Conference on Harmonisation Guidelines for Good Clinical Practice. Written informed consent was obtained from all volunteers prior to enrollment.

### Study Participants

Healthy adult volunteers aged 18–45 were recruited, with exclusion criteria including immunodeficiency, previous experimental challenge with Nlac, contra-indication to ciprofloxacin as a potential rescue therapy, or allergy to soya, as a constituent of the inoculum. In Mali, individuals colonized with Nlac at screening were excluded to avoid confounding culture data. Full eligibility criteria are detailed in Supplementary Table 1.

### Dosing

**Figure 1.**
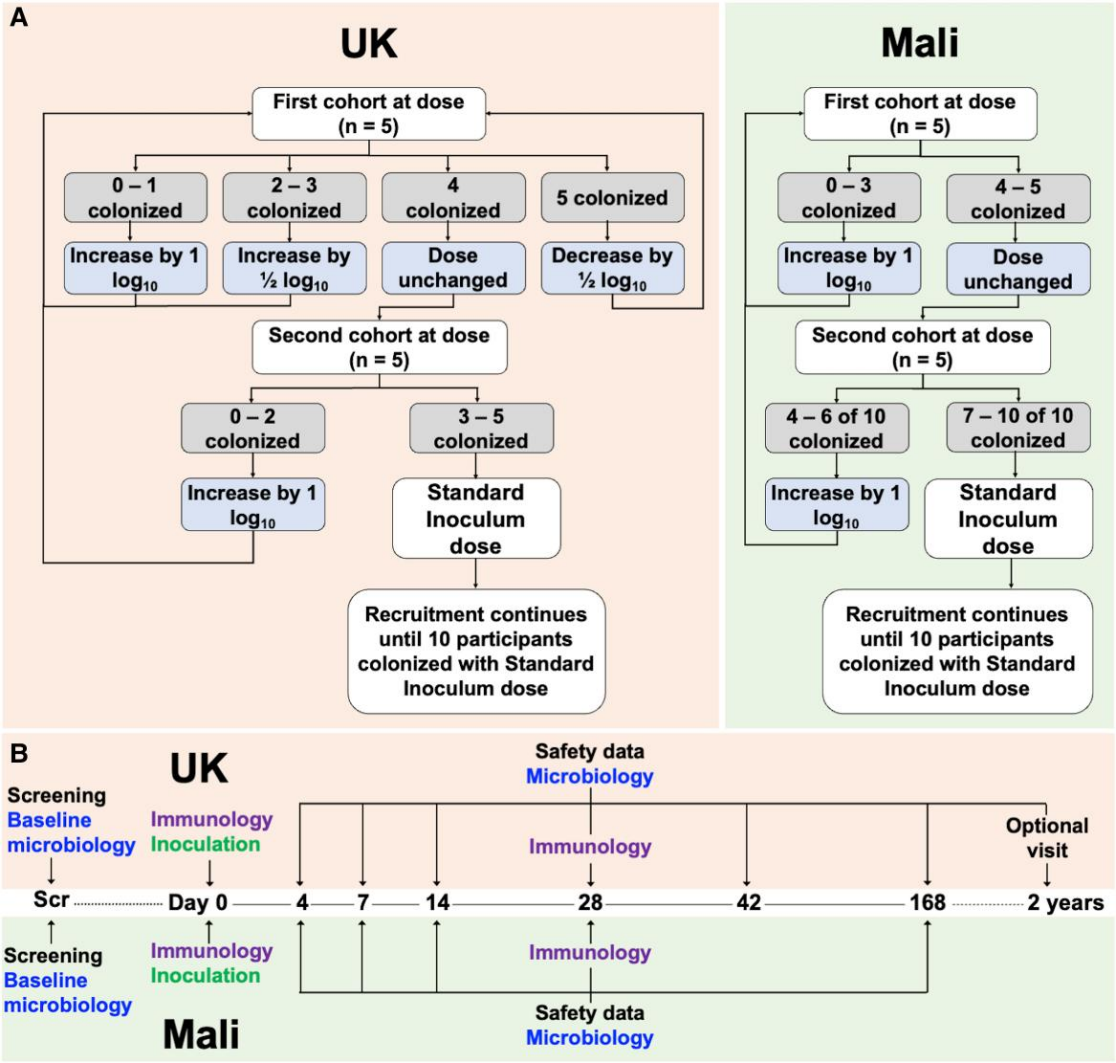
Study design. Dose-ranging strategies (*A*): participants were enrolled according to protocol-defined dose-ranging strategies to establish the standard inoculum dose in the United Kingdom (LHS, pink) and Mali (RHS, green). A starting dose of 10^5^ CFU was used in the United Kingdom. The standard inoculum dose identified in the United Kingdom was used as the starting dose in Mali. For both studies, if the maximum pre-specified inoculum dose of 10^7^ CFU was reached, this was identified as the standard inoculum dose regardless of colonization fraction. Study timeline (*B*): timeline with planned visits, interventions, and clinical samples comparing the UK (top, pink) and Mali (bottom, green) studies. Scr = screening visit; day 0–168 = visit time point in days from inoculation. Screening = informed consent, medical screening, and assessment of eligibility; baseline microbiology/microbiology = throat swab (United Kingdom and Mali) and nasal wash (United Kingdom only) to assess Nlac colonization; immunology = blood sampling for measurement of IgG with specificity to anti-Nlac- and anti-Nmen deoxycholate-extracted outer membrane vesicles (dOMV); inoculation = nasal inoculation with appropriate dose of reconstituted Nlac. Optional 2-year visit (United Kingdom only) (visit window 18 months to 3 years post-inoculation).

Eligible participants were nasally inoculated with LyoNlac according to a protocol-defined dose-ranging strategy aiming to identify a study-specific standard inoculum dose, defined as the dose which induced nasopharyngeal colonization in approximately 80% of participants by day 14 post-challenge (United Kingdom) or at least 70% of participants by day 7 post-challenge (Mali), or the maximum pre-specified dose (10^7^ CFU) if the desired colonization fraction was not achieved (Figure 1*A*). In the UK study, the starting dose was 10^5^ CFU, based on previous studies using frozen stocks of Nlac [26, 27]. The standard inoculum dose identified in the UK study was used as a starting dose in Mali. Participants were inoculated in dose cohorts of 5, with a review of safety and colonization data prior to enrollment of the next cohort. The dose was then repeated or escalated/de-escalated in 0.5–1 log increments according to the colonization fraction, as detailed in Figure 1*A*, until the standard inoculum dose was identified. A target colonization rate of 70%–80% was selected as likely to be clinically impactful but below a level of saturation. Recruitment was continued until at least 10 participants were colonized following inoculation with the standard inoculum dose with a maximum sample size of 35 (United Kingdom) and 100 (Mali) to allow completion of the dose-ranging process.

### Procedures

LyoNlac ampoules were reconstituted with 1 mL sterile water, incubated at room temperature for 5 min and then further diluted as calculated to achieve the intended inoculum dose in 1 mL. Sterile water was used as the diluent for the UK study due to previously observed improved duration of inoculum stability in comparison to PBS diluent, with the aim of optimizing feasibility for future field use. However, due to concerns about reactogenicity (see Results-Safety), the diluent was changed to PBS for the Mali study, as PBS had previously shown to be well tolerated and sufficiently stable following its use to dilute frozen Nlac stocks [25–27, 32]. All inoculum doses were administered within 30 min of dilution. Dilutional viability counting was conducted for each inoculum to determine the actual dose administered (confirmed inoculum dose) as previously described [33].

The inoculation procedure was conducted with the participant lying supine with their neck extended. 0.5 mL of inoculum was administered to each nostril by Pasteur pipette. The participant remained supine for 5 min following inoculation and was then observed for 20–30 min.

Participants attended study visits for monitoring of safety, microbiological, and immunological parameters over 168 days (Figure 1*B*). In the UK study, participants were invited to an optional 2-year visit to assess for ongoing colonization. Additional visits were arranged for any safety concerns if appropriate.

### Microbiology

Nasopharyngeal Nlac colonization was assessed by culture of oropharyngeal swabs (United Kingdom and Mali) and nasal wash samples (United Kingdom only) as previously described [33]. For the Mali study, only oropharyngeal swabs were collected due to an observed higher sensitivity as well as improved feasibility and acceptability in comparison to nasal wash sampling.

In brief, oropharyngeal swabs were plated directly on GC-selective agar plates supplemented with 40 μg mL^−1^ 5-bromo-4-chloro-3-indolyl-β-D-galactopyranoside (GC + Xgal). Nasal wash samples were centrifuged at 500 g for 10 min to pellet mucus and host cells. The supernatant was centrifuged for a further 10 min at 5000 g. The pellet was resuspended in 1 mL of supernatant, and then 50 μL and 250 μL aliquots were plated onto GC + Xgal. All plates were incubated for 24–48 h at 37°C, 5% CO_2_.

Colonies morphologically consistent with *Neisseria* were identified as putative Nlac (blue) or other *Neisseria* (white). Representative Nlac from each clinical sample were sub-cultured and stored for analysis at −80 °C. Colonization with the inoculum strain was confirmed by Y92-1009-specific PCR of genomic DNA extracted from at least one putative Nlac isolate per colonized participant (Supplementary material and Supplementary Table 2). White colonies were identified using API or MALDI-ToF and colonization with Nmen was noted.

Colonized participants were defined as those with any positive culture of Nlac up to day 14 post-challenge.

### Immunology

Serum Nlac- and Nmen-specific immunoglobulin G (Nlac-IgG, Nmen-IgG) titers were measured by enzyme-linked immunosorbent assay (ELISA) using plates coated with Nlac (Y92-1009)- and Nmen (H44/76)-derived deoxycholate extracted outer membrane vesicles (dOMV) as previously described [27, 29] with reference to a positive control serum extracted from whole blood taken from a single donor 28 days following Bexsero vaccination and demonstrated to produce a strong anti-Nlac and anti-Nmen IgG signal. Investigators performing serological assays and analyses were blinded to dose and colonization status.

### Safety

Solicited and unsolicited adverse event data were collected at each study visit, and participants were encouraged to contact the study team if adverse events occurred between study visits. Following self-reported reactogenicity in the UK study, reactogenicity data were collected for 20 min post-inoculation in the Mali study. For the UK study, safety was overseen by an external Data and Safety Monitoring Board. For the Mali study, safety data were reviewed at weekly online internal safety committee meetings.

### Statistical Analysis

Statistical analysis was performed using GraphPad Prism software version 9.5.1. Data were assessed for normality using Shapiro–Wilk. Mann–Whitney and Wilcoxon signed-ranks matched paired tests were used to assess unpaired and paired continuous data, respectively. Fisher's exact test was used to compare proportions between groups. All tests were 2-tailed and *P* values ≤ .05 were considered statistically significant.

## RESULTS

### Recruitment and Study Populations

**Figure 2.**
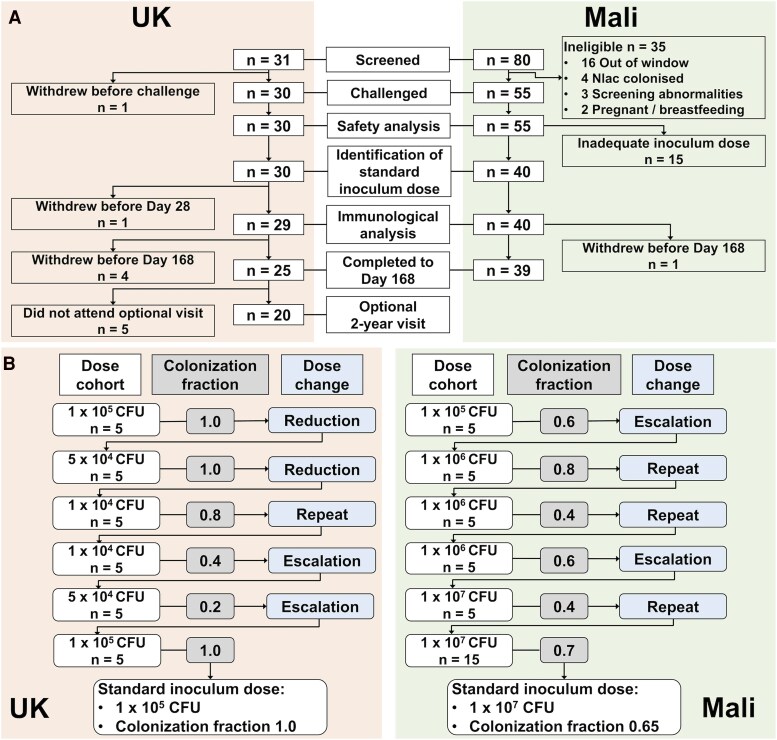
Participant enrollment. Study flow diagram (*A*): participants screened, challenged and included in each analysis in the United Kingdom (LHS, pink) and Mali (RHS, green). Participants in Mali who received a hypertonic inoculum with inadequate Nlac dose are reported as “Inadequate inoculum” (n = 15). These participants were followed up for safety but were not included in any further analyses. *B*, Identification of the standard inoculum dose: enrollment to intended dose cohorts with colonization fraction for each cohort and subsequent dose-ranging step leading to identification of the protocol-defined standard inoculum dose with overall colonization fraction at that dose for the United Kingdom (*A*) and Mali (*B*). Colonization fraction is defined as the proportion of participants in each cohort, or of those who received the standard inoculum dose, with at least one positive culture of *N. lactamica* from nasal wash or throat swab samples taken between inoculation and day 14 post-inoculation.

Participants screened, enrolled and inoculated with Nlac, and who completed follow-up are detailed in Figure 2*A*. In Mali, a PBS dilution error was identified for the first 3 cohorts, resulting in a hypertonic inoculum with resulting inadequate dose of Nlac. These 15 participants were excluded from the colonization and immunogenicity analyses but were followed up for safety. Participant demographics for participants screened and challenged per protocol are shown in Supplementary Table 3.

At screening, nasopharyngeal carriage of wild-type Nlac was 5% (n = 4/80) in Mali and 0% (n = 0/31) in the United Kingdom. Carriage of Nmen was seen in 8.8% (n = 7/80) in Mali and 9.7% (n = 3/31) in the United Kingdom.

### Colonization Status Following Inoculation and Identification of the Standard Inoculum Dose

The standard inoculum dose identified in the United Kingdom was 1 × 10^5^ CFU, achieving a colonization fraction of 100% (n = 10/10). In Mali, the dose-ranging process commenced at this dose and was escalated to the maximum dose of 1 × 10^7^ CFU, which achieved a colonization fraction of 65% (n = 13/20) (Figure 2*B*). The confirmed inoculum dose following viable count of the inoculum and the overall colonization fraction, at each intended dose, are summarized in Supplementary Table 4. At least 1 Nlac isolate for each colonized participant was confirmed by PCR to be the challenge strain (Y92-1009).

There were no significant differences in demographics or baseline Nmen carriage between colonized and non-colonized participants (Supplementary Table 5).

### Duration of Colonization

**Figure 3.**
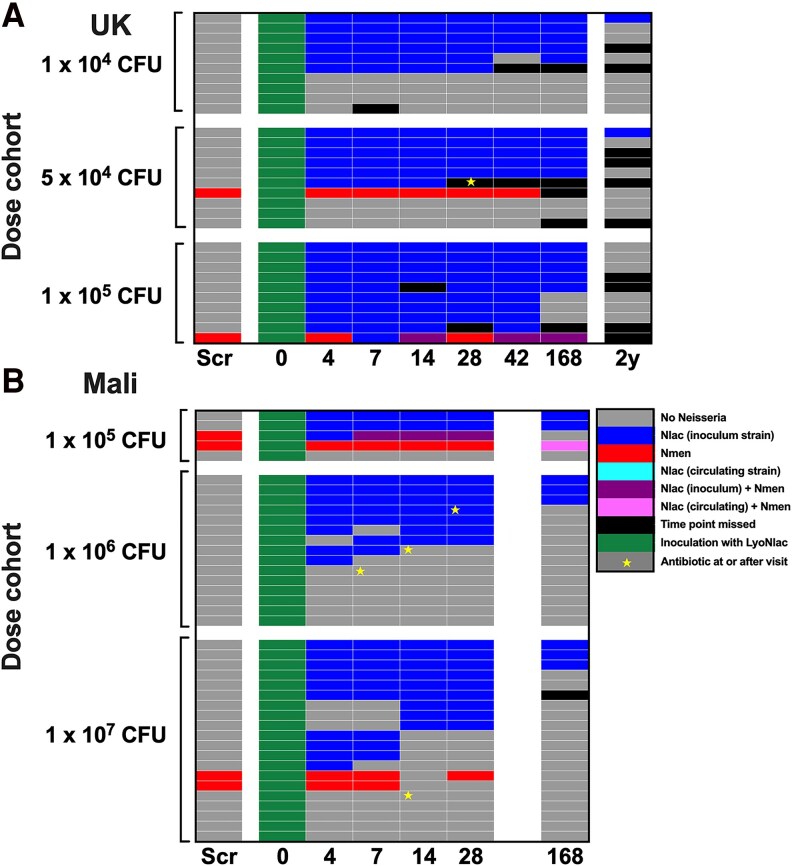
Colonization over time. Colonization status of each individual participant in the United Kingdom as defined by culture of at least 1 colony of Nlac or Nmen from either a nasal wash or throat swab at each time point (*A*), or in Mali as defined by culture of at least one colony of Nlac or Nmen from a throat swab at each time point (*B*). Scr = screening visit; numbers 0–168 indicate visit time point in days post-inoculation; 2y = optional 2-year visit (United Kingdom only, *B*). Inoculum or circulating (non-inoculum) strain of Nlac confirmed by Y92-1009-specific PCR of putative Nlac colonies. Antibiotics given for non-study related purposes: see Supplementary Table 6.

Colonization for each participant over time is shown in Figure 3. In the UK study (Figure 3*A*), of those participants who became Nlac colonized, this was detected by day 4 post-challenge in 95% (n = 21/22) and by day 7 in 100% (n = 22/22). One participant withdrew prior to the day 28 visit and a further 2 prior to the day 168 visit. Of those colonized participants who attended follow-up, 90% (n = 20/21) remained colonized at day 28 and 79% (n = 15/19) remained colonized at day 168. At the optional 2-year visit, 15% (n = 2/13) of previously colonized participants who attended remained colonized.

In the Mali study (Figure 3*B*), of those participants who became Nlac colonized, colonization was not detected until the day 14 visit in 3 participants, with the remainder detected by day 4 (n = 21/25, 84%) or 7 (n = 22/25, 88%). One colonized participant was lost to follow-up prior to the day 168 visit. Of those colonized participants who attended follow-up, 76% (n = 19/25) remained colonized at day 28 and 33% (n = 8/24) remained colonized at day 168. One participant, non-colonized following challenge, was newly Nlac colonized at the day 168 visit. This was confirmed by PCR to be a non-Y92 strain. Several participants received antibiotics during the study for non-study related reasons which in one case appeared to be associated with loss of carriage (Supplementary Table 6).

### Baseline Serology

**Figure 4.**
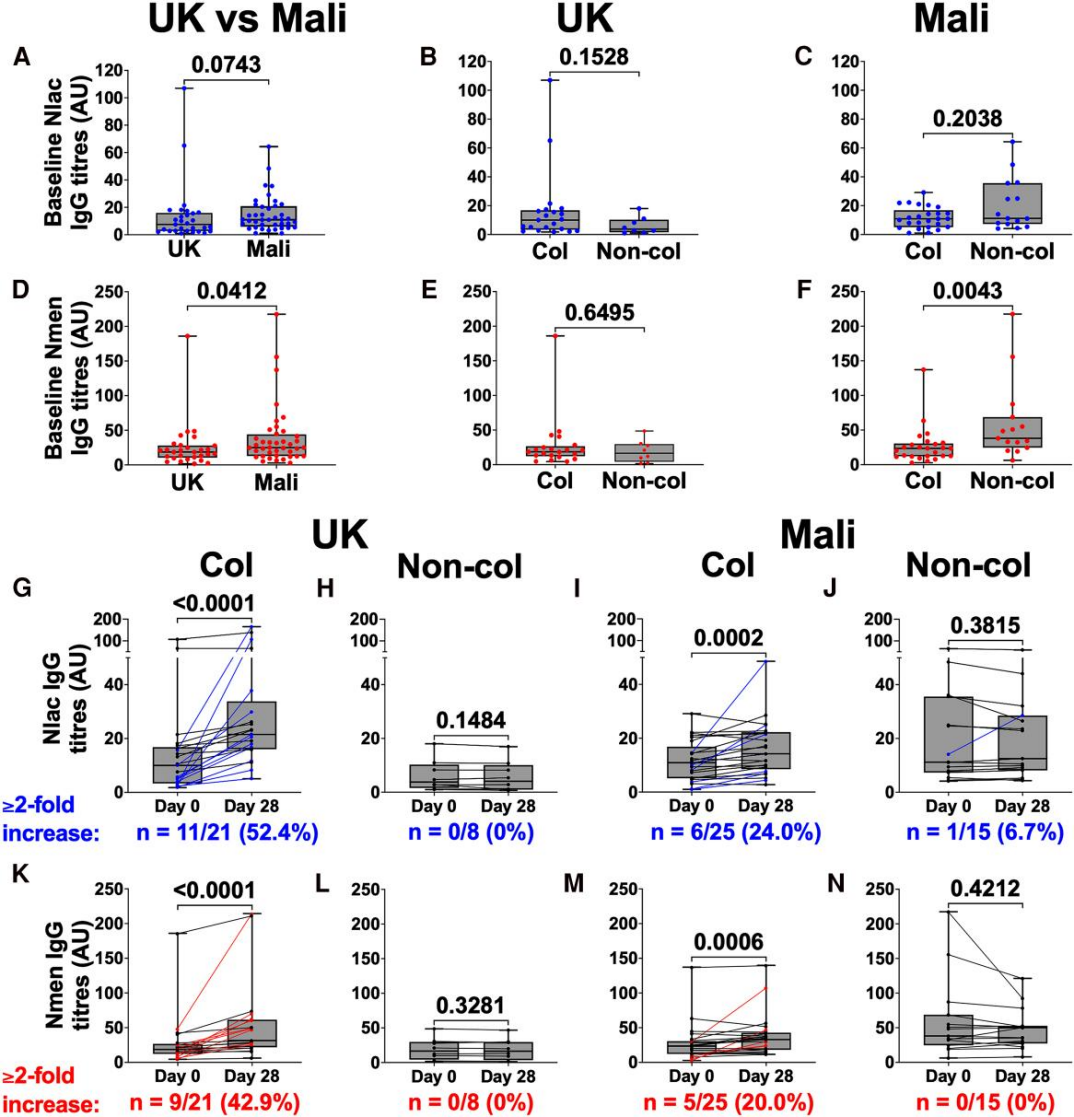
*Neisseria lactamica*– and *Neisseria meningitidis*–specific IgG titers. Sera collected from participants both prior to challenge with Nlac (day 0) and 28 days post-Nlac challenge (day 28) were assayed for IgG with specificity to dOMV derived from (i) Nlac Y92-1009 (anti-Nlac dOMV titers) and (ii) Nmen strain, H44/76 (anti-Nmen dOMV titers). Reciprocal antibody titers were interpolated with reference to a single control serum from an Nlac-colonized individual collected 28 days after challenge in a separate study. Anti-Nlac and anti-Nmen dOMV titers of participants enrolled in the United Kingdom (n = 29) and Mali (n = 40) who were subsequently challenged per protocol with Nlac and followed up to day 28 are shown, with each point representing the interpolated reciprocal titer of 1 participant at 1 time point. Serological data from all intended dose levels are included. Boxes show the median and interquartile range of each dataset and bars represent range. Day 0 (baseline) (*A–F*) anti-Nlac dOMV IgG (blue points, *A–C*) and anti-Nmen dOMV IgG (red points, *D–F*) titers are shown, with comparison between those enrolled in the United Kingdom (n = 29) and Mali (n = 40) (*A* and *D*), and comparing those who would (*Col*) or would not (*Non-col*) go on to become colonized by Nlac, in the United Kingdom (*B* and *E*, n = 29) or Mali (*C* and *F*, n = 40) are shown. *P* values derived using Mann–Whitney *U* test.

Baseline (pre-inoculation) Nlac-IgG and Nmen-IgG titers are shown in Figure 4*A*–*F*. There was a trend toward higher Nlac-IgG (Figure 4*A*) and significantly higher Nmen-IgG (Figure 4*D*) in Mali in comparison to the United Kingdom—median titer [interquartile range] Nlac-IgG UK: 7.51 [2.74–15.90], Mali: 10.97 [5.865–20.89] (*P* = .07) and Nmen-IgG UK: 18.66 [10.45–27.95], Mali: 24.98 [12.92–44.04] (*P* = .04). No difference in baseline titers of Nlac-IgG was seen between subsequently colonized and non-colonized participants in either study (Figure 4*B* and *C*). Participants who subsequently became Nlac colonized had significantly lower baseline Nmen-IgG in Mali (*P* = .004, Figure 4*F*) but not in the United Kingdom (Figure 4*E*).

### Serological Response to Colonization

Nlac- and Nmen-IgG titers at day 28 post-challenge were compared to baseline (Figure 4*G*–*N* and Supplementary Tables 7 and 8). An increase in Nlac-IgG and Nmen-IgG titers from pre-challenge to day 28 post-challenge was observed in colonized participants—median fold-change [interquartile range] United Kingdom: Nlac-IgG 2.24 [1.37–4.24], Nmen-IgG 1.39 [1.20–3.70] and Mali: Nlac-IgG 1.31 [1.04–1.94], Nmen-IgG 1.32 [0.99–1.73] with several participants having a greater than 2-fold increase in titer. In contrast, no significant increase in either Nlac- or Nmen-IgG titers was seen in non-colonized participants, in either study. No increase in serological response to colonization was observed with increasing inoculum dose (Supplementary Tables 7–9).

### Adverse Events

**Figure 5.**
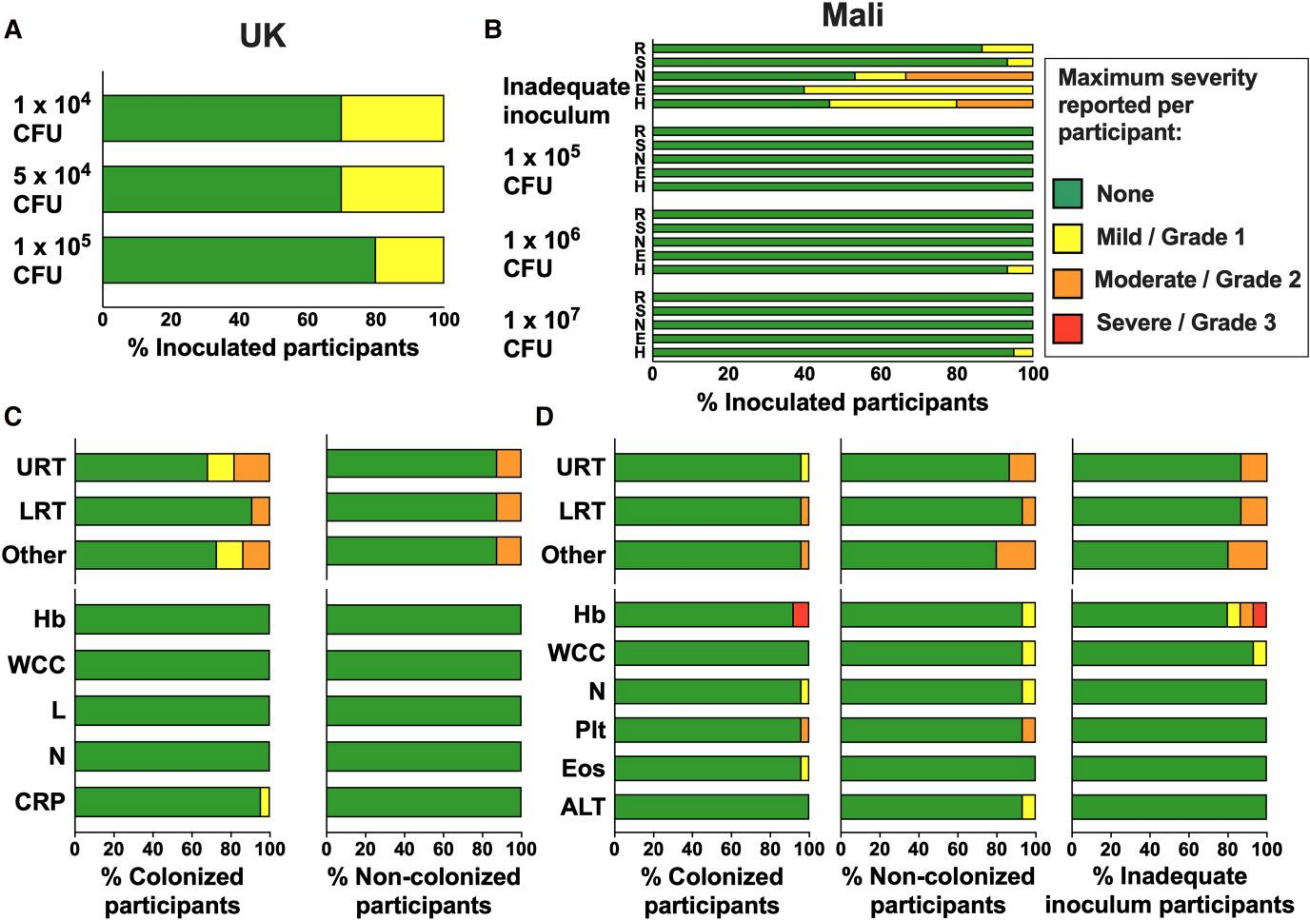
Reactogenicity and adverse events. Reactogenicity and adverse events presented as a proportion of participants per group, maximum severity per participant reported. Green = nil; yellow = mild/grade 1; orange = moderate/grade 2; red = severe/grade 3. United Kingdom: self-reported nasal irritation/stinging following inoculation, presented by intended inoculum dose, 1 × 10^4^ CFU (n = 10), 5 × 10^4^ CFU (n = 10), 1 × 10^5^ CFU (n = 10) (*A*). Mali: solicited reactogenicity within 25 min following inoculation, presented by intended inoculum dose. Participants who received a hypertonic inoculum with inadequate Nlac dose (*Inadequate inoculum*, n = 15), 1 × 10^5^ CFU (n = 15), 1 × 10^6^ CFU (n = 15), 1 × 10^7^ CFU (n = 20). R, rhinorrhea; S, sneezing; N, nasal irritation/stinging; E, eye watering; H, headache (*B*). Adverse events occurring between inoculation and day 28 in the UK study comparing participants who were colonized with Nlac at any point from inoculation to day 14 post-inoculation (*Colonized*, n = 22) and those who were not (*Non-colonized*, n = 8) (*C*). Adverse events occurring between inoculation and day 28 in the Mali study comparing participants who were colonized with Nlac at any point from inoculation to day 14 post-inoculation (*Colonized*, n= 25), those who were not (*Non-colonized*, n = 15), and those who received a hypertonic inoculum with inadequate Nlac dose (*Inadequate inoculum*, n = 15) (*D*). URT, upper respiratory tract symptoms; LRT, lower respiratory tract symptoms; Other, other adverse events; Hb, hemoglobin; WCC, white cell count; L, lymphocyte count; N, neutrophil count; CRP, C-reactive protein; Plt, platelet count; Eos, eosinophil count; ALT, alanine transferase. Blood parameters graded according to study-specific ranges based on institutional normal parameters, with a change of at least one grading band from baseline (see study protocols in Supplementary material).

Adverse events are summarized in Figure 5. In the United Kingdom, n = 8/30 (27%) participants reported transient nasal stinging following inoculation (Figure 5*A*). In Mali, mild to moderate reactogenicity symptoms were reported in n = 13/15 (87%) participants who received a hypertonic, inadequate inoculum (Figure 5*B*). Of those participants who received a per-protocol inoculum, n = 2/40 (5%) reported mild self-resolving headache with no other reactogenicity symptoms reported.

Additional adverse events occurring up to day 28, comparing colonized and non-colonized volunteers in each study are summarized in Figure 5*C* and *D*. Mild to moderate upper and lower respiratory tract symptoms occurred in both studies, with no difference observed between colonized and non-colonized participants. Other adverse events such as headache, injuries, gastroenteritis, or malaria and out of range laboratory parameters occurred in a small number of participants in both studies but none were assessed as being related to inoculation or colonization with Nlac.

The confirmed inoculum dose, determined by viable count of the residual inoculum, was reported to be higher than the acceptable upper limit for 2 participants in the United Kingdom. For one participant, this was reported as greater than 10 times the intended dose and so was reported as an adverse event of special interest. Neither participant reported any symptoms related to this. No other serious adverse events occurred in either study.

## DISCUSSION

We have demonstrated that nasal inoculation with a lyophilized preparation of Nlac (LyoNlac) safely and rapidly induces long-standing, nasopharyngeal colonization in a high proportion of healthy adult volunteers in the United Kingdom (100%) and Mali (65%). Successful colonization induced a humoral immune response, with an increase in both Nlac- and Nmen-IgG.

Reactogenicity was observed in both studies. In the United Kingdom, this was mild nasal stinging which self-resolved within a few minutes and was concluded to be due to the use of water as the inoculum diluent. In Mali, significant reactogenicity was observed in those participants who received an inadequate inoculum, likely due to the incorrect use of a hypertonic diluent. However, for those participants who received a correctly prepared inoculum with PBS as a diluent, the reactogenicity profile was well within acceptable limits.

In previous studies, inocula were prepared from frozen bacterial stocks [25–27]. However, the requirement for temperature-controlled storage and complex inoculum preparation techniques limited the real-world utility of the model. Lyophilization is commonly used to enable stable long-term storage and transport of bacterial strains with reconstitution yielding viable bacteria [34]. Uniquely, we have shown that lyophilized Nlac was readily reconstituted and thrived in a colonization niche. Furthermore, the finding that colonization induced by LyoNlac is immunogenic opens the way for further studies to evaluate the impact on meningococcal colonization and transmission in a high disease burden setting.

The use of lyophilized bacteria to induce colonization has been previously demonstrated with the *B. pertussis* vaccine candidate, BPZE1. This live-attenuated strain has been developed as a lyophilized product which is microbiologically stable at ambient temperatures for at least 2 years [35]. Nasal administration of BPZE1 induces transient nasopharyngeal colonization and seroconversion [36, 37] and reduces nasopharyngeal colonization with *B. pertussis* [38]. Together these data provide proof of principle that live lyophilized bacterial products have utility as mucosal vaccines and support the approach taken with LyoNlac.

The observation that colonization with LyoNlac induced both Nlac- and Nmen-IgG in UK and Malian participants is consistent with our previous findings [26, 27]. We have previously demonstrated that Nlac colonization induces Nmen-cross-reactive IgG in serum, IgA- and IgG-secreting plasma cells, and immunological memory [27]. We have not assessed the generation of anti-Nmen serum bactericidal activity (SBA) in these studies as recent epidemiological and clinical studies suggest that Nlac does not generate potent SBA [26, 39, 40]. However, we have previously demonstrated that inoculation with genetically modified Nlac expressing the anti-Nmen vaccine antigen, *Neisseria* adhesin A, can be utilized to enhance the anti-Nmen response to include generation of anti-Nmen SBA [32]. In future work, we will undertake further genetic modifications of the strain to optimize this potential response.

We have previously demonstrated that experimental Nlac colonization reduces Nmen carriage in healthy adults in the United Kingdom [25]. In the current studies, we have demonstrated the induction of immunogenic colonization, but not the translation of this to an impact on Nmen carriage or disease in the meningitis belt. These studies did not recapitulate the previously observed displacement of Nmen from carriage, with only 2 out of 6 participants who were Nmen colonized at baseline becoming successfully colonized with the inoculum strain of Nlac (Figure 3). However, it must be noted that these studies are far smaller in size than the study in which the displacement phenomenon was originally observed [25]. It should also be noted that the previously demonstrated Nmen displacement phenomenon was limited only to those who were successfully Nlac colonized and that preexisting Nmen colonization was associated with a reduction in successful Nlac colonization following challenge [25]. Essentially therefore, in these studies, Nlac was unable to establish colonization in the majority of Nmen-colonized individuals. The reasons for this are unknown.

However, as the highest risk of meningococcal disease is known to be within the first 2 weeks following Nmen acquisition [41], it is likely that the clinical utility of Nlac would be to prevent new acquisition of Nmen, both during seasonal hyper-endemicity where Nmen carriage prevalence remains low, or to limit the dramatic increase in carriage associated with large epidemics [14]. Individuals already colonized with Nmen, while not free from risk of developing meningococcal disease, become less likely to do so the longer Nmen is carried, so the primary aim of an Nlac-based intervention would be prevention of Nmen spread rather than displacement of resident Nmen. The current studies were not designed to demonstrate a reduction in Nmen acquisition.

Therefore, further studies, designed and powered to assess the impact of Nlac colonization on Nmen colonization, transmission and disease in the African meningitis belt, and whether protection can be optimized via genetic modification of Nlac, are important next steps.

It is notable that we achieved an Nlac colonization fraction of 100% in the UK cohort, higher than the aim of “approximately 80%.” By contrast, in Mali, the colonization fraction did not significantly increase with increasing dose, and only 65% colonization was achieved with the maximum pre-specified dose. This suggests that the maximum possible colonization fraction in Mali population is lower than in the United Kingdom. Possible mechanisms to explain this observation include differences in (1) the composition of the nasopharyngeal microbiome, which modulates resistance against Nlac colonization, and (2) baseline immunity, acquired as a consequence of repeated episodes of transient carriage of Nlac or other Neisseriaceae, which induce cross-reactive immunological memory. In support of this second hypothesis, we have previously demonstrated that experimentally induced Nlac colonization results in the generation of adaptive immune responses, including immunological memory, to Nmen [26, 27]. Furthermore, we have shown that baseline anti-Nmen IgG memory B cell responses positively correlate with Nlac-specific plasma B cell frequencies and IgG titer increases following Nlac challenge and negatively correlate with Nlac colonization density following challenge [27]. These observations strongly suggest that preexisting cross-reactive adaptive immune responses may drive protection against colonization. Consistent with this hypothesis, in the current study, we observed that baseline Nlac/Nmen-IgG titers were higher in Mali than the United Kingdom and that participants protected against Nlac colonization following challenge in Mali had significantly higher baseline Nmen-IgG titers as compared to their unprotected counterparts (Figure 4*F*).

Some limitations in comparisons between the 2 sites are acknowledged. While these 2 studies are similar in design, there were differences in eligibility criteria, follow-up schedule, procedures, and sampling techniques. In addition, the healthy adults enrolled in these studies may not be entirely representative of the population targeted in any possible future intervention, which would ideally require inoculation of the whole population. Further consideration must be given to the potential impact of variables such as age, geographical location within the meningitis belt, immunological parameters, and microbiota composition on the ability of LyoNlac to induce nasopharyngeal colonization. To address the critical issue of variability in colonization potential across different ages, we intend to assess the acceptability, safety, and efficacy of LyoNlac inoculation in a healthy UK pediatric population, which we view as essential preliminary data prior to larger field trials to assess the impact on Nmen transmission in the meningitis belt.

Reactive vaccination remains the gold standard preventative strategy during outbreaks of meningococcal disease in the meningitis belt [9]. However, the mobilization of vaccination campaigns is dependent upon surveillance data with serogroup identification, the availability of serogroup-matched vaccine, and infrastructure for transport and administration, all of which can cause a delay in response time [10].

Potential advantages of LyoNlac include more rapid deployment due to the lack of cold-chain requirement, ease of administration, and serogroup independence. The previously demonstrated impact on Nmen colonization was evident by 2 weeks post-inoculation, the earliest sampling time point reported [25]. In the current studies, Nlac colonization was detectable by day 4 after challenge in the majority of those who became colonized. It is therefore plausible that an impact on Nmen carriage could occur more rapidly than 2 weeks, which would be beneficial in limiting spread during a meningococcal outbreak.

LyoNlac could therefore be a useful adjunct, given prior to or alongside serogroup-matched conjugate vaccines, to mitigate against delays in vaccine availability and therefore slowing the progression of an outbreak while it is still relatively small.

In conclusion, lyophilized Nlac rapidly induces asymptomatic, immunogenic nasopharyngeal colonization in the United Kingdom and Mali. In the future, this approach can be investigated as a preventative strategy to interrupt meningococcal transmission in an outbreak setting.

## Supplementary Material

ofaf809_Supplementary_Data

## Supplementary Data

Supplementary materials are available at *Open Forum Infectious Diseases* online. Consisting of data provided by the authors to benefit the reader, the posted materials are not copyedited and are the sole responsibility of the authors, so questions or comments should be addressed to the corresponding author.

## References

[1] GBD Meningitis Collaborators . Global, regional, and national burden of meningitis, 1990–2016: a systematic analysis for the global burden of disease study 2016. Lancet Neurol 2018; 17:1061–82. 30507391 10.1016/S1474-4422(18)30387-9 PMC6234314

[2] Broutin H, Philippon S, Constantin de Magny G, Courel MF, Sultan B, Guegan JF. Comparative study of meningitis dynamics across nine African countries: a global perspective. Int J Health Geogr 2007; 6:29. 17623084 10.1186/1476-072X-6-29 PMC1939699

[3] Agier L, Martiny N, Thiongane O, et al Towards understanding the epidemiology of Neisseria meningitidis in the African meningitis belt: a multi-disciplinary overview. Int J Infect Dis 2017; 54:103–12. 27826113 10.1016/j.ijid.2016.10.032

[4] Kristiansen PA, Diomande F, Ba AK, et al Impact of the serogroup A meningococcal conjugate vaccine, MenAfriVac, on carriage and herd immunity. Clin Infect Dis 2013; 56:354–63. 23087396 10.1093/cid/cis892

[5] WHO . Control of epidemic meningitis in countries in the African meningitis belt, 2023. Wkly Epidemiol Rec 2024; 99:551–64.

[6] Soeters HM, Diallo AO, Bicaba BW, et al Bacterial meningitis epidemiology in five countries in the meningitis belt of sub-saharan Africa, 2015–2017. J Infect Dis 2019; 220:S165–74. 31671441 10.1093/infdis/jiz358 PMC6853282

[7] McNamara LA, Neatherlin J. WHO Strategic Advisory Group of Experts on Immunization recommendations for use of a novel pentavalent meningococcal ACWXY vaccine: a critical step towards ending meningococcal epidemics in Africa. J Travel Med 2024; 31:taae002. 10.1093/jtm/taae002 PMC11841484 38195714

[8] WHO . Meningococcal vaccines: WHO position paper on the use of multivalent meningococcal conjugate vaccines in countries of the African meningitis belt. Wkly Epidemiol Rec 2024; 99:1–10.

[9] WHO . Standard operating procedures for surveillance of meningitis preparedness and response to epidemics in Africa 2018 [2nd]. [Available at: https://www.who.int/publications/i/item/standard-operating-procedures-for-surveillance-of-meningitis-preparedness-and-response-to-epidemics-in-africa.

[10] Hassan A, Mustapha GU, Lawal BB, et al Time delays in the response to the Neisseria meningitidis serogroup C outbreak in Nigeria—2017. PLoS One 2018; 13:e0199257. 29920549 10.1371/journal.pone.0199257 PMC6007901

[11] Stephens DS, Greenwood B, Brandtzaeg P. Epidemic meningitis, meningococcaemia, and Neisseria meningitidis. Lancet 2007; 369:2196–210. 17604802 10.1016/S0140-6736(07)61016-2

[12] Christensen H, May M, Bowen L, Hickman M, Trotter CL. Meningococcal carriage by age: a systematic review and meta-analysis. Lancet Infect Dis 2010; 10:853–61. 21075057 10.1016/S1473-3099(10)70251-6

[13] Diallo K, Trotter C, Timbine Y, et al Pharyngeal carriage of Neisseria species in the African meningitis belt. J Infect 2016; 72:667–77. 27018131 10.1016/j.jinf.2016.03.010 PMC4879866

[14] Koutangni T, Mainassara B, Mueller H, E J. Incidence, carriage and case-carrier ratios for meningococcal meningitis in the African meningitis belt: a systematic review and meta-analysis. PLoS One 2015; 10:e0116725. 25658307 10.1371/journal.pone.0116725 PMC4319942

[15] Clark SA, Borrow R. Herd protection against meningococcal disease through vaccination. Microorganisms 2020; 8:1675. 33126756 10.3390/microorganisms8111675 PMC7693901

[16] Maiden MC, Ibarz-Pavon AB, Urwin R, et al Impact of meningococcal serogroup C conjugate vaccines on carriage and herd immunity. J Infect Dis 2008; 197:737–43. 18271745 10.1086/527401 PMC6767871

[17] Balmer P, Burman C, Serra L, York LJ. Impact of meningococcal vaccination on carriage and disease transmission: a review of the literature. Hum Vaccin Immunother 2018; 14:1118–30. 29565712 10.1080/21645515.2018.1454570 PMC5989891

[18] Coldiron ME, Assao B, Page AL, et al Single-dose oral ciprofloxacin prophylaxis as a response to a meningococcal meningitis epidemic in the African meningitis belt: a 3-arm, open-label, cluster-randomized trial. PLoS Med 2018; 15:e1002593. 29944651 10.1371/journal.pmed.1002593 PMC6019097

[19] Gold R, Goldschneider I, Lepow ML, Draper TF, Randolph M. Carriage of Neisseria meningitidis and Neisseria lactamica in infants and children. J Infect Dis 1978; 137:112–21. 415097 10.1093/infdis/137.2.112

[20] Cartwright KA, Stuart JM, Jones DM, Noah ND. The Stonehouse survey: nasopharyngeal carriage of meningococci and Neisseria lactamica. Epidemiol Infect 1987; 99:591–601. 3123263 10.1017/s0950268800066449 PMC2249239

[21] Bennett JS, Griffiths DT, McCarthy ND, et al Genetic diversity and carriage dynamics of Neisseria lactamica in infants. Infect Immun 2005; 73:2424–32. 15784588 10.1128/IAI.73.4.2424-2432.2005 PMC1087434

[22] Walsh L, Clark SA, Derrick JP, Borrow R. Beyond the usual suspects: reviewing infections caused by typically-commensal Neisseria species. J Infect 2023; 87:479–89. 37797844 10.1016/j.jinf.2023.09.007

[23] Coen PG, Cartwright K, Stuart J. Mathematical modelling of infection and disease due to Neisseria meningitidis and Neisseria lactamica. Int J Epidemiol 2000; 29:180–8. 10750621 10.1093/ije/29.1.180

[24] Olsen SF, Djurhuus B, Rasmussen K, et al Pharyngeal carriage of Neisseria meningitidis and Neisseria lactamica in households with infants within areas with high and low incidences of meningococcal disease. Epidemiol Infect 1991; 106:445–57. 1904825 10.1017/s0950268800067492 PMC2271871

[25] Deasy AM, Guccione E, Dale AP, et al Nasal inoculation of the commensal Neisseria lactamica inhibits carriage of Neisseria meningitidis by young adults: a controlled human infection study. Clin Infect Dis 2015; 60:1512–20. 25814628 10.1093/cid/civ098

[26] Evans CM, Pratt CB, Matheson M, et al Nasopharyngeal colonization by Neisseria lactamica and induction of protective immunity against Neisseria meningitidis. Clin Infect Dis 2011; 52:70–7. 21148522 10.1093/cid/ciq065

[27] Dale AP, Theodosiou AA, Gbesemete DF, et al Effect of colonisation with Neisseria lactamica on cross-reactive anti-meningococcal B-cell responses: a randomised, controlled, human infection trial. Lancet Microbe 2022; 3:e931–43. 36462524 10.1016/S2666-5247(22)00283-X PMC7615047

[28] Pandey A, Cleary DW, Laver JR, et al Microevolution of Neisseria lactamica during nasopharyngeal colonisation induced by controlled human infection. Nat Commun 2018; 9:4753. 30420631 10.1038/s41467-018-07235-5 PMC6232127

[29] Theodosiou AA, Bogaert D, Cleary DW, et al Controlled human infection model of Neisseria lactamica in late pregnancy investigating mother-to-infant transmission in the UK: a single-arm pilot trial. Lancet Microbe 2025; 6:100986. 39986292 10.1016/j.lanmic.2024.100986

[30] Greenwood B . Manson lecture. Meningococcal meningitis in Africa. Trans R Soc Trop Med Hyg 1999; 93:341–53. 10674069 10.1016/s0035-9203(99)90106-2

[31] Smith E, M M, Mandla A, et al **2023**. Available at: https://www.hic-vac.org/sites/default/files/SMART PRACTISES V2.1 7 June 2023.pdf.

[32] Laver JR, Gbesemete D, Dale AP, et al A recombinant commensal bacteria elicits heterologous antigen-specific immune responses during pharyngeal carriage. Sci Transl Med 2021; 13:eabe8573. 34233953 10.1126/scitranslmed.abe8573 PMC7615050

[33] Dale AP, Gbesemete D, Read RC, Laver JR. Neisseria lactamica controlled human infection model. In: Bidmos FA, Bossé J, Langford P, eds. Methods in molecular biology. Vol 2414. New York: Springer US; 2022:387–404. 10.1007/978-1-0716-1900-1_21 34784048

[34] Morgan CA, Herman N, White PA, Vesey G. Preservation of micro-organisms by drying; a review. J Microbiol Methods 2006; 66:183–93. 16632005 10.1016/j.mimet.2006.02.017

[35] Thalen M, Debrie AS, Coutte L, et al Manufacture of a stable lyophilized formulation of the live attenuated Pertussis vaccine BPZE1. Vaccines (Basel) 2020; 8:523. 32933132 10.3390/vaccines8030523 PMC7565209

[36] Buddy Creech C, Jimenez-Truque N, Kown N, et al Safety and immunogenicity of live, attenuated intranasal Bordetella pertussis vaccine (BPZE1) in healthy adults. Vaccine 2022; 40:6740–6. 36220716 10.1016/j.vaccine.2022.09.075

[37] Keech C, Miller VE, Rizzardi B, et al Immunogenicity and safety of BPZE1, an intranasal live attenuated pertussis vaccine, versus tetanus-diphtheria-acellular pertussis vaccine: a randomised, double-blind, phase 2b trial. Lancet 2023; 401:843–55. 36906345 10.1016/S0140-6736(22)02644-7

[38] Gbesemete D, Ramasamy MN, Ibrahim M, et al Efficacy, immunogenicity, and safety of the live attenuated nasal pertussis vaccine, BPZE1, in the UK: a randomised, placebo-controlled, phase 2b trial using a controlled human infection model with virulent Bordetella pertussis. Lancet Microbe 2025; 6:101211. 41344352 10.1016/j.lanmic.2025.101211

[39] Trotter C, Findlow J, Balmer P, et al Seroprevalence of bactericidal and anti-outer membrane vesicle antibodies to Neisseria meningitidis group B in England. Clin Vaccine Immunol 2007; 14:863–8. 17494636 10.1128/CVI.00102-07 PMC1951059

[40] Gorringe AR, Taylor S, Brookes C, et al Phase I safety and immunogenicity study of a candidate meningococcal disease vaccine based on Neisseria lactamica outer membrane vesicles. Clin Vaccine Immunol 2009; 16:1113–20. 19553555 10.1128/CVI.00118-09 PMC2725532

[41] Wilder-Smith A, Barkham TM, Ravindran S, Earnest A, Paton NI. Persistence of W135 Neisseria meningitidis carriage in returning Hajj pilgrims: risk for early and late transmission to household contacts. Emerg Infect Dis 2003; 9:123–6. 12533295 10.3201/eid0901.020131 PMC2873737

