# Supplementary material for: Controlled Human Infection of Healthy Adults With Lyophilized Neisseria lactamica Induces Asymptomatic, Immunogenic Nasopharyngeal Carriage in the United Kingdom and Mali
Source: Open Forum Infect Dis. 2026 Jan 7;13(1):ofaf809. doi: 10.1093/ofid/ofaf809 (PMC12822493; doi:10.1093/ofid/ofaf809)
Supplement: ofaf809_Supplementary_Data [file ofaf809_supplementary_data.zip › ofaf809_Supplementary_Data.docx]

**SUPPLEMENTARY MATERIAL**

**LyoNlac quality control**

Analysis of LyoNlac was conducted to ensure that following a standard reconstitution step, ampoules contained cultivable Nlac with no contamination, with a viability of at least the maximum dose required as per protocol (1 x 10^7^ CFU), termed the minimum viable product. Stability of the viability over time, and across different storage conditions, was also assessed.

A batch of 136 ampoules was produced for use in these studies. Following production, quality control checks were performed on 13 out of 136 ampoules, selected evenly across the batch. All ampoules were plated on non-selective media to confirm purity and no contamination was detected. Viable counts were performed in triplicate, using serial ten-fold dilutions of each reconstituted ampoule. The mean batch viability was 3.4 x 10^8^ CFU (95% CI 3.2-3.6 x 10^8^). The remaining ampoules were stored in a temperature-monitored refrigerator.

11 months after production, the mean batch viability was 2.8 x 10^8^ CFU (95% CI 2.1-3.6 x 10^8^ CFU), determined from the viable count of 9 further ampoules. Prior to the start of study B the remaining ampoules were transferred to Mali under refrigerated conditions. The batch viability in Mali was 1.1 x 10^8^ CFU (95% CI 5.7 x 10^7^ – 1.6 x 10^8^ CFU), determined from the viable count of 3 ampoules, approximately 25 months after production. Thus some reduction in viability of LyoNlac was seen over time, but it remained well above the minimum viable product after more than two years of refrigerated storage (Supplementary Figure 1A).

In order to assess the stability of LyonLac under different temperature conditions, ampoules of a second batch were exposed to different storage temperatures over 91 days. Four different conditions were used, all cycling between two temperatures for 16 and 8 hour periods, to simulate day/night storage at ambient temperatures (25 °C / 37 °C) and intermittent refrigeration (4 °C / 37 °C). The batch viability was determined at weekly intervals by viable count of four ampoules per temperature condition and timepoint (Figure S1B-E). The fastest loss of viability was seen in ampoules exposed to the laboratory simulated ambient day/night cycle, cycling 16 hours at 37 °C and 8 hours at 25 °C. In these ampoules the minimum viable product of 10^7^ CFU per ampoule was reached after 84 days storage. Those ampoules exposed to the refrigerated cycle of 16 hours at 4 °C and 8 hours at 37 °C showed negligible degradation of viability over 91 days.


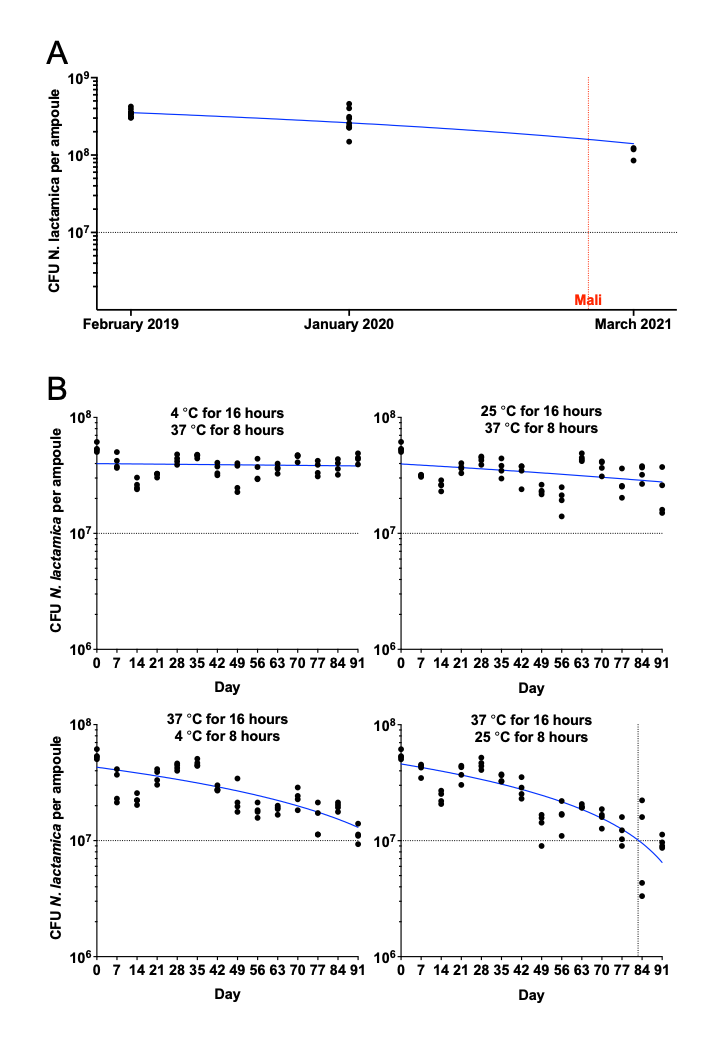


**Supplementary Figure 1: LyoNlac stability**

(**A**) LyoNlac viable count over time with continuous refrigeration, points represent mean of triplicate counts of ampoules, blue line represents linear regression, dotted horizontal line represents minimal viable product (1 x 10^7^ CFU), vertical red dotted line indicates time of refrigerated transport to Mali.

(**B**) Viability of LyoNlac exposed to temperature day/night (16h/8h) cycling as indicated. Points represent mean of duplicate counts of ampoules, blue line represents linear regression, dotted horizontal line represents minimal viable product (1 x 10^7^ CFU), vertical black dotted line indicates time of reaching minimal viable product.

**Supplementary Table 1: Eligibility criteria**

| **Inclusion criteria** | **UK** | **Mali** |
| --- | --- | --- |
| Healthy adults aged 18 to 45 years inclusive on the day of enrolment | ✔ | ✔ |
| Residing outside the demographic surveillance area |  | ✔ |
| Fully conversant in the English language | ✔ |  |
| Able and willing (in the investigator’s opinion) to comply with all study requirements | ✔ | ✔ |
| Provide written informed consent to participate in the trial | ✔ | ✔ |
| For females only, willingness to practice continuous effective contraception during the study (Study A) / until the Day 28 visit (Study B) and a negative pregnancy test on the day(s) of screening and inoculation | ✔ | ✔ |
| TOPS registration completed and no conflict found | ✔ |  |
| **Exclusion criteria** |  |  |
| Current active smokers defined as having smoked a cigarette or cigar in the last four weeks | ✔ |  |
| *N. lactamica* detected on throat swab taken at the screening visit |  | ✔ |
| Individuals who have a current infection at the time of inoculation |  | ✔ |
| Individuals who have been involved in other clinical trials involving receipt of an investigational product over the last 12 weeks or if there is planned use of an investigational product during the study period | ✔ | ✔ |
| Individuals who have previously been involved in clinical trials investigating meningococcal vaccines or experimental challenge with *N. lactamica* | ✔ |  |
| Use of systemic antibiotics within the period 30 days prior to the challenge | ✔ | ✔ |
| Any confirmed or suspected immunosuppressive or immune-deficient state, including HIV infection; malignancy, asplenia; recurrent, severe infections and chronic (more than 14 days) immunosuppressant medication within the past 6 months (topical steroids are allowed) | ✔ | ✔ |
| Use of immunoglobulins or blood products within 3 months prior to enrolment. | ✔ | ✔ |
| History of allergic disease or reactions likely to be exacerbated by any component of the inoculum, specifically soya | ✔ | ✔ |
| Contraindications to the use of ciprofloxacin, specifically a history of epilepsy, prolonged QT interval, hypersensitivity to quinolones or a history of tendon disorders related to quinolone use | ✔ |  |
| Any clinically significant abnormal finding on clinical examination or screening investigations | ✔ | ✔ |
| Any other significant disease, disorder, or finding which may significantly increase the risk to the volunteer because of participation in the study, affect the ability of the volunteer to participate in the study or impair interpretation of the study data, for example recent surgery to the nasopharynx | ✔ | ✔ |
| Occupational, household or intimate contact with immunosuppressed persons | ✔ | ✔ |
| Pregnancy, lactation (UK and Mali) or intention to become pregnant during the study (UK) | ✔ | ✔ |

**Y92-1009 Specific PCR**

Bacterial isolates from clinical samples were analysed by strain specific PCR to distinguish between colonisation with the challenge strain (Y92-1009) and colonisation with community acquired strains of Nlac. Putative Nlac colonies were subcultured on chocolate blood agar (CBA) plates, confirming pure growth by visual inspection. DNA extraction from collated individual colonies was performed using the Genomic DNA Purification Kit (Fisher Scientific, Loughborough UK), according to the manufacturer’s instructions.

A multiplex PCR was performed using three custom primer pairs (Table S4). Primer pair Z targeted the *lacZ* gene, present in all Nlac strains, whilst Primer pairs A and C were specific to sequences uniquely found in strain Y92-1009 according to meta-analysis of genomic sequences deposited in PubMLST (1).

Resultant amplicons were separated by agarose gel electrophoresis and bands were visualised under ultraviolet illumination. Images were captured by Image Lab software (Bio-Rad). The presence of all three, correctly-sized amplicons: A, C and Z were required to define the source of the genomic DNA as Y92-1009. The presence of amplicon Z alone, or amplicon Z along with one or more amplicons of unanticipated size indicated the source of the genomic DNA was a non-Y92-1009 strain of Nlac.

**Supplementary Table 2: Y92-1009 specific PCR primer pairs**

|  | **Primer sequence (F: forward, R: reverse)** | **Amplicon length (bases)** | **Annealing temperature (°C)** | **Extension time (seconds)** |
| --- | --- | --- | --- | --- |
| **A** | F: GTGCTGAATTTATAGACGGGC  R: AAGCTAGGTCTACTTGGTTTAG | 388 | 62 | 7.76-11.64 |
| **C** | F: AGGGACCGACATCTTTCATAC  R: TTGCAGGCTCTTTCCAAAC | 594 | 64 | 11.88-17.82 |
| **Z** | F: CGGGCAAACTTGCGCGG  R: GCAAACCGAAACGGGGCAGG | 745 | 72 | 14.90-22.35 |

1. Jolley KA, Bray JE, Maiden MCJ. Open-access bacterial population genomics: BIGSdb software, the PubMLST.org website and their applications. Wellcome Open Res. 2018;3:124.

**Supplementary Table 3: Participant demographics**

|  | **Screened** | | **Challenged** | |
| --- | --- | --- | --- | --- |
|  | **UK** | **Mali** | **UK** | **Mali** |
| **n** | 31 | 80 | 30 | 40 |
| **Age:**  **Median**  **(IQR)** | 28.5  (23.0-33.1) | 26.3  (21.3-36.4) | 29.0  (22.8-33.5) | 24.3  (21.3-33.9) |
| **Biological sex:**  **Male n (%)**  **Female n (%)** | 9 (29.0)  22 (71.0) | 24 (30.0)  56 (70.0) | 8 (26.7)  22 (73.3) | 13 (32.5)  27 (67.5) |

Demographic information for participants screened and challenged per protocol. NB: The 15 participants challenged with an incorrect inoculum dose in Mali are not included in the challenged group as not per protocol. Age expressed as Median (interquartile range), *p* value derived from Mann Whitney test. Biological sex expressed as n (%), *p* value derived from Fisher’s exact test. Age unknown for two screened and one challenged participant in Mali so participants excluded from age results.

**Supplementary Table 4: Colonization fraction at each intended inoculum dose**

|  | **UK** | | | **Mali** | | |
| --- | --- | --- | --- | --- | --- | --- |
| **Intended dose**  **(CFU)** | 1 x 10^4^ | 5 x 10^4^ | 1 x 10^5^ | 1 x 10^5^ | 1 x 10^6^ | 1 x 10^7^ |
| **Number of participants** | 10 | 10 | 10 | 5 | 15 | 20 |
| **Colonization fraction** | 0.60  (0.31-0.83) | 0.60  (0.31-0.83) | 1.00  (0.72-1.00) | 0.60  (0.23-0.93) | 0.60  (0.36-0.80) | 0.65  (0.43-0.82) |
| **Confirmed inoculum dose** | 9.7 x 10^3^  (7.4x10^3^, 1.1x10^4^) | 4.4 x 10^4^  (4.0x10^4^, 6.2x10^4^) | 8.0 x 10^4^  (5.1x10^4^, 2.2x10^5^) | 7.7 x 10^4^  (7.5x10^4^, 7.7x10^4^) | 1.0 x 10^6^  (8.8x10^5^, 1.1x10^6^) | 1.5 x 10^7^  (1.4x10^7^, 1.6x10^7^) |

Overall number of participants challenged, colonization fraction (95% confidence interval), and median confirmed inoculum dose (interquartile range) calculated by viable count of the residual inoculum, for each intended inoculum dose in the UK and Mali.

Supplementary Table 5: Comparison of baseline characteristics

|  | UK | | | Mali | | |
| --- | --- | --- | --- | --- | --- | --- |
|  | Colonized | Non-colonized | *p* | Colonized | Non-colonized | *p* |
| n | 22 | 8 |  | 25 | 15 |  |
| Age | 30.2  (25.5-37.5) | 26.0  (21.7-30.0) | 0.09 | 22.5  (19.5-32.3) | 28.6  (23.1-36.3) | 0.09 |
| Biological sex:  Male  Female | 5 (22.7)  17 (77.3) | 3 (37.5)  5 (62.5) | 0.64 | 9 (36)  16 (64) | 4 (26.7)  11 (73.3) | 0.73 |
| Baseline Nmen carriage | 1 (4.5) | 1 (12.5) | 0.47 | 1 (4) | 3 (20.0) | 0.14 |

Baseline characteristics of participants enrolled in the UK and Mali with comparison between participants subsequently colonized and non-colonized with Nlac in each study. Age expressed as Median (interquartile range), *p* value derived from Mann Whitney test. Biological sex and Nmen carriage expressed as n (%), *p* value derived from Fisher’s exact test. *p* values ≤ 0.05 considered statistically significant.

**Supplementary Table 6: Non-study related antibiotic use**

| **Study site** | **Dose** | **Timepoint** | **Antibiotic** | **Indication** | **Associated with loss of colonization** |
| --- | --- | --- | --- | --- | --- |
| UK | 5 x 10^4^ CFU | Day 28 | Oral metronidazole  Single dose | Bacterial vaginosis | Unknown (Did not attend Day 28 visit) |
| Mali | 1 x 10^6^ CFU | Day 20 | Oral amoxicillin  7 days | Bronchitis | No (ongoing colonization at Day 28 visit) |
| Mali | 1 x 10^6^ CFU | Day 7 | Oral norfloxacin  7 days | Suspected urogenital infection | Likely (no longer colonized at Day 14 visit) |
| Mali | 1 x 10^6^ CFU | Day 6 | Oral metronidazole and ciprofloxacin  7 days | Gastroenteritis | No (non-colonized) |
| Mali | 1 x 10^7^ CFU | Day 9 | Oral amoxicillin  7 days | Rhinobronchitis | No (non-colonized) |

Use of antibiotics within the study period for non-study related indications

Supplementary Table 7: Serological data: UK

|  |  | | Nlac IgG titer | | | | Nmen IgG titer | | | |
| --- | --- | --- | --- | --- | --- | --- | --- | --- | --- | --- |
| Inoculum dose (CFU) | | | **Day 0** | **Day 28** | **Absolute change** | **Fold change** | **Day 0** | **Day 28** | **Absolute change** | **Fold change** |
|  | | Colonized participants | | | | | | | | |
| 1 | 10^4^ | | 2.26 | 5.06 | 2.8 | 2.24 | 4.44 | 6.17 | 1.73 | 1.39 |
| 2 | 10^4^ | | 106.96 | 138.67 | 31.71 | 1.3 | 185.97 | 211.72 | 25.75 | 1.14 |
| 3 | 10^4^ | | 5.1 | 21.52 | 16.42 | 4.22 | 4.68 | 47.11 | 42.43 | 10.07 |
| 4 | 10^4^ | | 4.93 | 17.92 | 12.99 | 3.63 | 22.46 | 28.14 | 5.68 | 1.25 |
| 5 | 10^4^ | | 2.52 | 106.38 | 103.86 | 42.21 | 8.15 | 62.35 | 54.2 | 7.65 |
| 6 | 10^4^ | | 16.24 | 23.23 | 6.99 | 1.43 | 18.66 | 22.8 | 4.14 | 1.22 |
| 7 | 5x10^4^ | | 3.91 | 16.67 | 12.76 | 4.26 | 20.83 | 48.88 | 28.05 | 2.35 |
| 8 | 5x10^4^ | | 21.44 | 25.28 | 3.84 | 1.18 | 40.6 | 73.81 | 33.21 | 1.82 |
| 9 | 5x10^4^ | | 1.78 | 11.59 | 9.81 | 6.51 | 6.25 | 25.62 | 19.37 | 4.1 |
| 10 | 5x10^4^ | | 7.51 | 11.05 | 3.54 | 1.47 | 42.66 | 50.53 | 7.87 | 1.18 |
| 11 | 5x10^4^ | | 13.14 | 23 | 9.86 | 1.75 | 13.29 | 27.58 | 14.29 | 2.08 |
| 12 | 5x10^4^ | | 65.11 | 65.23 | 0.12 | 1 | 28.3 | 31.44 | 3.14 | 1.11 |
| 13 | 10^5^ | | 15.55 | 165.36 | 149.81 | 10.63 | 48.03 | 214.37 | 166.34 | 4.46 |
| 14 | 10^5^ | | 2.76 | 37.76 | 35 | 13.68 | 18.42 | 60.64 | 42.22 | 3.29 |
| 15 | 10^5^ | | 10.48 | 16.29 | 5.81 | 1.55 | 21.91 | 29.2 | 7.29 | 1.33 |
| 16 | 10^5^ | | 10 | 29.81 | 19.81 | 2.98 | 16.05 | 15.64 | -0.41 | 0.97 |
| 17 | 10^5^ | | 17.35 | 19.1 | 1.75 | 1.1 | 15.47 | 20.74 | 5.27 | 1.34 |
| 18 | 10^5^ | | 14.23 | 15.83 | 1.6 | 1.11 | 21.43 | 20.44 | -0.99 | 0.95 |
| 19 | 10^5^ | | 2.1 | 8.19 | 6.09 | 3.9 | 13.37 | 16.83 | 3.46 | 1.26 |
| 20 | 10^5^ | | 18.1 | 26.13 | 8.03 | 1.44 | 24.87 | 49.76 | 24.89 | 2 |
| 21 | 10^5^ | | 5.66 | 20.54 | 14.88 | 3.63 | 11.12 | 70.19 | 59.07 | 6.31 |
| 22 | 10^5^ | | 15.55 | 165.36 | 149.81 | 10.63 | 48.03 | 214.37 | 166.34 | 4.46 |
| Median | | | 10.00 | 21.52 | 9.81 | 2.24 | 18.66 | 31.44 | 14.29 | 1.39 |
| IQR | | | 3.34-16.80 | 16.06-33.79 | 3.69-18.12 | 1.37-4.24 | 12.21-26.59 | 21.77-61.50 | 3.80-37.72 | 1.20-3.70 |
|  | | Non-colonized participants | | | | | | | | |
| 1 | 10^4^ | | 0.98 | 0.67 | -0.31 | 0.68 | 20.88 | 19.78 | -1.1 | 0.95 |
| 2 | 10^4^ | | 8.26 | 8.18 | -0.08 | 0.99 | 9.77 | 9.01 | -0.76 | 0.92 |
| 3 | 10^4^ | | 4.67 | 5.33 | 0.66 | 1.14 | 12.16 | 12.92 | 0.76 | 1.06 |
| 4 | 10^4^ | | 18.01 | 16.93 | -1.08 | 0.94 | 27.6 | 29.78 | 2.18 | 1.08 |
| 5 | 5x10^4^ | | 1.2 | 1.1 | -0.1 | 0.92 | 2.39 | 1.62 | -0.77 | 0.68 |
| 6 | 5x10^4^ | | 10.91 | 10.7 | -0.21 | 0.98 | 48.66 | 46.71 | -1.95 | 0.96 |
| 7 | 5x10^4^ | | 2.72 | 2.79 | 0.07 | 1.03 | 30.44 | 28.82 | -1.62 | 0.95 |
| 8 | 5x10^4^ | | 2.85 | 0.92 | -1.93 | 0.32 | 1.6 | 1.28 | -0.32 | 0.8 |
| Median | | | 3.76 | 4.06 | -0.16 | 0.96 | 16.52 | 16.35 | -0.77 | 0.95 |
| IQR | | | 1.58-10.25 | 0.97-10.07 | -0.89-0.03 | 0.74-1.02 | 4.24-29.73 | 3.47-29.54 | -1.49-0.49 | 0.83-1.04 |

Sera collected from participants in the UK both prior to Nlac challenge (Day 0) and 28 days post-Nlac challenge (Day 28), were assayed for IgG with specificity to dOMV derived from: (i) Nlac Y92-1009 (Nlac IgG titers) and (ii) Nmen strain, H44/76 (Nmen IgG titers). Reciprocal antibody titers were interpolated with reference to a single control serum from an Nlac-colonized individual collected 28 days after challenge in a separate study. Nlac-IgG and Nmen-IgG titers at Day 0, Day 28, absolute change from Day 0 to Day 28 and fold-change from Day 0 to Day 28 shown for participants who became Nlac-colonized (**Colonized**) or who did not become Nlac-colonized (**Non-colonized**) following per protocol challenge and who were followed up to Day 28 are shown. Median and interquartile range of each dataset also shown.

Supplementary Table 8: Serological data: Mali

| Inoculum dose (CFU) | | Nlac IgG titer | | | | | | | Nmen IgG titer | | | | | | |
| --- | --- | --- | --- | --- | --- | --- | --- | --- | --- | --- | --- | --- | --- | --- | --- |
|  |  | **Day 0** | **Day 28** | | **Absolute change** | | **Fold change** | | **Day 0** | **Day 28** | | **Absolute change** | | **Fold change** | |
|  | | Colonized participants | | | | | | | | | | | | | |
| 1 | 10^5^ | 21.05 | 21.71 | | 0.66 | | 1.03 | | 137.22 | 139.84 | | 2.62 | | 1.02 | |
| 2 | 10^5^ | 10.08 | 21.53 | | 11.45 | | 2.14 | | 23.64 | 41.64 | | 18.00 | | 1.76 | |
| 3 | 10^5^ | 8.18 | 24.94 | | 16.76 | | 3.05 | | 31.51 | 36.85 | | 5.34 | | 1.17 | |
| 4 | 10^6^ | 21.90 | 19.30 | | -2.60 | | 0.88 | | 12.77 | 15.42 | | 2.65 | | 1.21 | |
| 5 | 10^6^ | 7.29 | 10.08 | | 2.79 | | 1.38 | | 28.51 | 29.78 | | 1.27 | | 1.04 | |
| 6 | 10^6^ | 3.80 | 7.64 | | 3.84 | | 2.01 | | 7.83 | 11.62 | | 3.79 | | 1.48 | |
| 7 | 10^6^ | 1.14 | 6.93 | | 5.79 | | 6.08 | | 5.02 | 30.23 | | 25.21 | | 6.02 | |
| 8 | 10^6^ | 14.48 | 48.58 | | 34.10 | | 3.35 | | 29.59 | 107.32 | | 77.73 | | 3.63 | |
| 9 | 10^6^ | 12.42 | 11.61 | | -0.81 | | 0.93 | | 44.89 | 43.96 | | -0.93 | | 0.98 | |
| 10 | 10^6^ | 20.39 | 22.07 | | 1.68 | | 1.08 | | 17.77 | 16.94 | | -0.83 | | 0.95 | |
| 11 | 10^6^ | 4.71 | 5.25 | | 0.54 | | 1.11 | | 13.35 | 13.34 | | -0.01 | | 1.00 | |
| 12 | 10^6^ | 7.93 | 11.74 | | 3.81 | | 1.48 | | 2.69 | 46.89 | | 44.20 | | 17.43 | |
| 13 | 10^7^ | 29.14 | 25.25 | | -3.89 | | 0.87 | | 24.94 | 37.25 | | 12.31 | | 1.49 | |
| 14 | 10^7^ | 11.69 | 17.22 | | 5.53 | | 1.47 | | 11.47 | 24.26 | | 12.79 | | 2.12 | |
| 15 | 10^7^ | 14.61 | 16.68 | | 2.07 | | 1.14 | | 41.49 | 38.05 | | -3.44 | | 0.92 | |
| 16 | 10^7^ | 1.00 | 4.41 | | 3.41 | | 4.41 | | 9.32 | 23.79 | | 14.47 | | 2.55 | |
| 17 | 10^7^ | 10.90 | 14.23 | | 3.33 | | 1.31 | | 20.38 | 19.77 | | -0.61 | | 0.97 | |
| 18 | 10^7^ | 5.02 | 9.37 | | 4.35 | | 1.87 | | 11.93 | 19.32 | | 7.39 | | 1.62 | |
| 19 | 10^7^ | 19.05 | 28.58 | | 9.53 | | 1.50 | | 33.57 | 56.59 | | 23.02 | | 1.69 | |
| 20 | 10^7^ | 22.21 | 22.76 | | 0.55 | | 1.02 | | 63.42 | 51.86 | | -11.56 | | 0.82 | |
| 21 | 10^7^ | 14.27 | 22.43 | | 8.16 | | 1.57 | | 25.02 | 32.92 | | 7.90 | | 1.32 | |
| 22 | 10^7^ | 3.15 | 2.75 | | -0.40 | | 0.87 | | 12.52 | 12.28 | | -0.24 | | 0.98 | |
| 23 | 10^7^ | 5.25 | 6.73 | | 1.48 | | 1.28 | | 12.60 | 14.92 | | 2.32 | | 1.18 | |
| 24 | 10^7^ | 10.90 | 14.23 | | 3.33 | | 1.31 | | 24.26 | 35.10 | | 10.84 | | 1.45 | |
| 25 | 10^7^ | 9.41 | 9.85 | | 0.44 | | 1.05 | | 23.55 | 35.66 | | 12.11 | | 1.51 | |
| Median | | 10.90 | 14.23 | | 3.33 | | 1.31 | | 23.55 | 32.92 | | 5.34 | | 1.32 | |
| IQR | | 5.14-16.83 | 8.51-22.25 | | 0.54-5.66 | | 1.04-1.94 | | 12.23-30.55 | 18.13-42.80 | | -0.13-13.63 | | 0.99-1.73 | |
|  | | Non-colonized participants | | | | | | | | | | | | | |
| 1 | 10^5^ | 11.19 | | 10.48 | | -0.71 | | 0.94 | 68.70 | | 51.54 | | -17.16 | | 0.75 |
| 2 | 10^5^ | 4.45 | | 5.22 | | 0.77 | | 1.17 | 6.27 | | 7.93 | | 1.66 | | 1.26 |
| 3 | 10^6^ | 48.41 | | 44.07 | | -4.34 | | 0.91 | 155.87 | | 121.33 | | -34.54 | | 0.78 |
| 4 | 10^6^ | 7.31 | | 8.13 | | 0.82 | | 1.11 | 38.14 | | 35.91 | | -2.23 | | 0.94 |
| 5 | 10^6^ | 5.39 | | 4.30 | | -1.09 | | 0.80 | 32.44 | | 22.52 | | -9.92 | | 0.69 |
| 6 | 10^6^ | 7.90 | | 9.08 | | 1.18 | | 1.15 | 34.32 | | 35.39 | | 1.07 | | 1.03 |
| 7 | 10^6^ | 14.05 | | 28.49 | | 14.44 | | 2.03 | 33.06 | | 50.31 | | 17.25 | | 1.52 |
| 8 | 10^6^ | 24.62 | | 23.53 | | -1.09 | | 0.96 | 55.08 | | 51.23 | | -3.85 | | 0.93 |
| 9 | 10^7^ | 35.56 | | 32.06 | | -3.50 | | 0.90 | 48.52 | | 50.15 | | 1.63 | | 1.03 |
| 10 | 10^7^ | 24.94 | | 22.85 | | -2.09 | | 0.92 | 20.31 | | 27.32 | | 7.01 | | 1.35 |
| 11 | 10^7^ | 64.32 | | 58.49 | | -5.83 | | 0.91 | 87.47 | | 78.49 | | -8.98 | | 0.90 |
| 12 | 10^7^ | 4.13 | | 4.76 | | 0.63 | | 1.15 | 19.21 | | 20.36 | | 1.15 | | 1.06 |
| 13 | 10^7^ | 9.41 | | 9.85 | | 0.44 | | 1.05 | 50.91 | | 52.72 | | 1.81 | | 1.04 |
| 14 | 10^7^ | 11.04 | | 12.49 | | 1.45 | | 1.13 | 24.65 | | 30.89 | | 6.24 | | 1.25 |
| 15 | 10^7^ | 36.07 | | 26.48 | | -9.59 | | 0.73 | 217.55 | | 92.54 | | -125.01 | | 0.43 |
| Median | | 11.19 | | 12.49 | | -0.71 | | 0.96 | 38.14 | | 50.15 | | 1.07 | | 1.03 |
| IQR | | 7.31-35.56 | | 8.13-28.49 | | -3.5-0.82 | | 0.91-1.15 | 24.65-68.70 | | 27.32-52.72 | | -9.92-1.81 | | 0.78-1.25 |

Sera collected from participants in Mali both prior to Nlac challenge (Day 0) and 28 days post-Nlac challenge (Day 28), were assayed for IgG with specificity to dOMV derived from: (i) Nlac Y92-1009 (Nlac IgG titers) and (ii) Nmen strain, H44/76 (Nmen IgG titers). Reciprocal antibody titers were interpolated with reference to a single control serum from an Nlac-colonized individual collected 28 days after challenge in a separate study. Nlac-IgG and Nmen-IgG titers at Day 0, Day 28, absolute change from Day 0 to Day 28 and fold-change from Day 0 to Day 28 shown for participants who became Nlac-colonized (**Colonized**) or who did not become Nlac-colonized (**Non-colonized**) following per protocol challenge and who were followed up to Day 28 are shown. Median and interquartile range of each dataset also shown.

Supplementary Table 9: Serological response stratified by intended inoculum dose

|  |  | UK | | | | Mali | | | |
| --- | --- | --- | --- | --- | --- | --- | --- | --- | --- |
|  | Intended inoculum dose (CFU) | 10^4^ | 5 x10^4^ | 10^5^ | *p* | 10^5^ | 10^6^ | 10^7^ | *p* |
|  | n | 6 | 6 | 9 |  | 3 | 9 | 13 |  |
| Nlac-IgG | Titer change  Median  (IQR) | 14.7  (5.9-49.8) | 6.8  (2.7-10.6) | 8.0  (3.8-27.4) | 0.32 | 11.5  (0.7-16.8) | 2.8  (-0.1-4.8) | 3.3  (0.5-4.9) | 0.41 |
|  | Fold change  Median  (IQR) | 2.9  (1.4-13.7) | 1.6  (1.1-4.8) | 3.0  (1.3-7.3) | 0.80 | 2.1  (1.0-3.1) | 1.4  (1.0-2.7) | 1.3  (1.0-1.5) | 0.61 |
| Nmen-IgG | Titer change  Median  (IQR) | 15.7  (3.5-45.4) | 16.8  (6.7-29.3) | 7.3  (1.5-50.7) | 0.96 | 5.3  (2.6-18.0) | 2.7  (-0.4-34.7) | 8.0  (-0.4-12.6) | 0.91 |
|  | Fold change  Median  (IQR) | 1.3  (1.2-8.3) | 1.9  (1.2-2.8) | 1.3  (1.1-3.9) | 0.97 | 1.2  (1.0-1.8) | 1.2  (1.0-4.8) | 1.5  (1.0-1.7) | 0.93 |

Sera collected from participants in Mali both prior to Nlac challenge (Day 0) and 28 days post-Nlac challenge (Day 28), were assayed for IgG with specificity to dOMV derived from: (i) Nlac Y92-1009 (Nlac-IgG titers) and (ii) Nmen strain, H44/76 (Nmen-IgG titers). Reciprocal antibody titers were interpolated with reference to a single control serum from an Nlac-colonized individual collected 28 days after challenge in a separate study. Absolute change in titer (Titer change) and fold change in Nlac-IgG and Nmen-IgG titers from Day 0 to Day 28 are shown for participants who became Nlac-colonized following per protocol challenge at each intended inoculum dose, and who were followed up to Day 28 are shown. Median and interquartile range of each dataset also shown. *p* values derived from Kruskall-Wallis test.
