# Supplementary material for: Controlled Human Infection of Healthy Adults With Lyophilized Neisseria lactamica Induces Asymptomatic, Immunogenic Nasopharyngeal Carriage in the United Kingdom and Mali
Source: Open Forum Infect Dis. 2026 Jan 7;13(1):ofaf809. doi: 10.1093/ofid/ofaf809 (PMC12822493; doi:10.1093/ofid/ofaf809)
Supplement: ofaf809_Supplementary_Data [file ofaf809_supplementary_data.zip › Lac5 (UK study) Protocol v3.0 clean.pdf]

A human controlled infection study to assess safety, colonisation and immunogenicity following nasal inoculation with reconstituted lyophilised wild type *Neisseria lactamica* : Lactamica 5

Sponsor Study reference: ERGO 31543

NHS REC Study Reference: 18/SC/0420

Protocol version: 3.0

Date: 28/09/2021

Chief Investigator: Professor R.C. Read

University Hospital Southampton NHS Foundation Trust

Version	Date	Authors	Modifications
1.6	22nd May 2018	Diane Gbesemete Jay Laver Robert Read	
2.0	21st November 2018	Diane Gbesemete Jay Laver Robert Read	Alteration to eligibility criteria<br>Clarification of assessment of laboratory results at screening<br>Adverse event grading moved to an SOP
3.0	28th September 2021	Diane Gbesemete Jay Laver Robert Read	Addition of optional 2 year follow up visit

A human controlled infection study to assess colonisation and immunogenicity following nasal inoculation with reconstituted lyophilised wild type *Neisseria lactamica*

Study references: ERGO 31543
RHM MED 1555

Chief Investigator: **Professor Robert C. Read**
University of Southampton
C Level
South Lab & Pathology Block
Southampton
University Hospital Southampton NHS Foundation Trust
Tremona Road, Southampton, SO16 6YD
Email: r.c.read@soton.ac.uk

Principal Investigator: **Professor Saul N. Faust**
NIHR Clinical Research Facility
University of Southampton
C Level, West Wing, Mailpoint 218
University Hospital Southampton NHS Foundation Trust
Tremona Road, Southampton, SO16 6YD
Email: s.faust@soton.ac.uk

Investigators: **Jay Laver**, Senior Research Fellow
University of Southampton
C Level South Lab & Pathology Block
Southampton
University Hospital Southampton NHS Foundation Trust
Tremona Road, Southampton, SO16 6YD
Email: j.r.laver@soton.ac.uk

Diane Gbesemete, Clinical Research Fellow
NIHR Clinical Research Facility
University of Southampton
C Level, West Wing, Mailpoint 218
University Hospital Southampton NHS Foundation Trust
Tremona Road, Southampton, SO16 6YD
Email: diane.gbesemete@uhs.nhs.uk

Andrew Vaughan, Research Fellow
University of Southampton
C Level
South Lab & Pathology Block
Southampton
University Hospital Southampton NHS Foundation Trust
Tremona Road, Southampton, SO16 6YD
Andrew.Vaughan@soton.ac.uk

**Trial Site:**	**NIHR-CRF**<br>University Hospital Southampton NHS Foundation Trust<br>C Level, West Wing, Mailpoint 218<br>University Hospital Southampton NHS Foundation Trust<br>Tremona Road, Southampton, SO16 6YD
**Sponsor**	**The University of Southampton**<br>University Road<br>Southampton<br>SO17 1BJ
**Funded by**	National Institute for Health Research, Health Protection Research Unit (NIHR HPRU)<br>University College London

Confidentiality Statement

This document contains confidential information that must not be disclosed to anyone other than the sponsor, the investigator team, and members of the independent ethics committee. This information cannot be used for any purpose other than the evaluation or conduct of the clinical investigation without the prior written consent of Professor R.C. Read**.**

Investigator Agreement

"I have read this protocol and agree to abide by all provisions set forth therein.

I agree to comply with the principles of the International Conference on Harmonisation Tripartite Guideline on Good Clinical Practice."

Professor R.C. Read	………………………	…………………
Chief Investigator	Investigator Signature	Date

Conflict of Interest

1. “According to the Declaration of Helsinki, 2008, I have read this protocol, and declare no/the following (delete as appropriate) conflict of interest”

Professor R.C. Read	………………………	……………….
Chief Investigator	Investigator Signature	Date

2. “According to the Declaration of Helsinki, 2008, I have read this protocol, and declare no/the following (delete as appropriate) conflict of interest”

Professor S.N. Faust	………………………	……………….
Principal Investigator	Investigator Signature	Date

3. “According to the Declaration of Helsinki, 2008, I have read this protocol, and declare no/the following (delete as appropriate) conflict of interest”

D. Gbesemete	………………………	……………….
Investigator	Signature	Date

4. “According to the Declaration of Helsinki, 2008, I have read this protocol, and declare no/the following (delete as appropriate) conflict of interest”

J.R.Laver	………………………	……………….
Investigator	Signature	Date

5. “According to the Declaration of Helsinki, 2008, I have read this protocol, and declare no/the following (delete as appropriate) conflict of interest”

A. Vaughan	………………………	……………….
Investigator	Signature	Date

Table of Contents

1. Synopsis 10

2. Abbreviations 12

3. Background and rationale 14

3.1 The rationale for the current study 14

3.2 *Neisseria lactamica* and *Neisseria meningitidis* 14

3.3 Carriage of *Neisseria spp.* 15

3.4 Immunity and carriage 15

3.5 Vaccine induced immunity to carriage 16

3.6 Human challenge with *Neisseria lactamica* 16

3.7 Lyophilised *N. lactamica* 16

4. Objectives 17

4.1 Primary objective 17

4.2 Secondary objectives 17

5. Description and justification of the study design 17

5.1 Overview 17

5.2 Study volunteers 18

5.3 Challenge procedure 18

5.4 First volunteers – safety 18

5.5 Assessment of colonisation 18

5.6 Dose ranging strategy 18

5.7 Duration of volunteer participation 20

5.8 Definition of the start and the end of the study 20

5.9 Potential benefits for the volunteers. 20

5.10 Involvement of the NHS Research Ethics Committee 20

6. Inoculum 20

6.1 Selection of strain of *N. lactamica* 20

6.2. Supply and storage of the stock strain 20

6.3 Lyophilisation of *N. lactamica* 20

6.4 Preparation of nasal inoculum 20

6.5 The optimal and safe dose of the inoculum 21

6.6 Monitoring of the *N. lactamica* dose administered to volunteers 21

7. Recruitment and withdrawal of trial volunteers 21

7.1 Recruitment 21

7.2 Volunteer information sheet 22

7.3 Screening visit 22

7.4 Inclusion and exclusion criteria 23
8. *N. lactamica* challenge (Day 0) 25
8.1 Trial site 25
8.2 Clinical team involved in the challenge 25
8.3 Infection control 25
8.4 Challenge Procedures 25
9. Follow up visits 27
9.1 Main study 27
9.2 Optional 2 year sub-study 27
10. Clinical and laboratory monitoring 28
10.1 Clinical team 28
10.2 Monitoring of volunteers 28
10.3 Management of possible *N. lactamica* disease 28
10.4 Potential adverse events 29
10.5 Withdrawal of volunteers 29
11 Laboratory procedures 30
11.1 Laboratory work 30
11.2 Processing and storage of samples 30
11.3 Labelling of samples 31
11.4 Microbiological sampling 31
11.5 Immunological analyses 31
11.6 Residual clinical samples 31
12. Assessment of safety 31
12.1 Definitions 31
12.2 Causality assessment 33
12.3 Reporting procedures for AEs 34
12.4 Safety profile review 36
12.5 Study committees 36
13. Analysis and Statistical considerations 37
13.1 Endpoints of the study 37
13.2 Sample size 37
13.3 Statistical analysis 37
14. Study quality and Management procedures 38
14.1 Investigator procedures 38
14.2 Monitoring 38
14.3 Study amendments 38

14.4 Protocol deviation....38
14.5 Quality Control, Quality Assurance and statutory inspection....38
14.6 Serious breaches....39
14.7 Study progress....39
14.8 Study completion/termination....39
14.9 Exploitation and dissemination....39
15. Ethics....39
15.1 Declaration of Helsinki....39
15.2 ICH guidelines for good clinical practice....39
15.3 Informed consent....40
15.4 Informing participants' General Practitioners....40
15.5 Research ethics committee....40
15.6 Volunteer confidentiality....40
16. Data handling and record keeping....40
16.1 Data handling....40
16.2 Record keeping....41
16.3 Source data and case report forms (CRFs)....41
16.4 Data protection....41
17. Financing and insurance....42
17.1 Financing....42
17.2 Insurance....42
17.3 Compensation for time....42
18. References....43

1. Synopsis

**Title**	**A human controlled infection study to assess colonisation and immunogenicity following nasal inoculation with reconstituted lyophilised wild type *Neisseria lactamica***
**Sponsor**	The University of Southampton
**Trial Centre**	NIHR Clinical Research Facility, Southampton University Hospital NHS Foundation Trust, Southampton, SO16 6YD
**Trial Sponsor Code**	ERGO 31543
**Design**	Prospective dose ranging human challenge study<br>Nasal inoculation with reconstituted, previously lyophilised *Neisseria lactamica* with dose escalation / de-escalation
**Population**	Healthy volunteers aged 18-45 years
**Sample size**	Total up to 35 volunteers<br>Dose ranging study – 5 volunteers per dose cohort
**Follow up duration**	Challenge at day 0, follow up on Days 4, 7, 14, 28, 42 and 168<br>2 year substudy – optional 2 year visit
**Planned Trial Period**	June 2018 – May 2022
**Primary Objective**	i. To assess the safety of nasal inoculation of healthy volunteers with reconstituted lyophilised wild type *Neisseria lactamica*
**Secondary Objectives**	i. To establish the dose of nasally administered reconstituted lyophilised *Neisseria lactamica* required to induce nasopharyngeal colonisation in approximately 80% of inoculees<br>ii. To demonstrate the immunogenicity of colonisation induced by reconstituted lyophilised wild type *Neisseria lactamica*<br>iii. To examine the kinetics and density of colonisation induced by reconstituted lyophilised wild type *Neisseria lactamica*<br>iv. To assess the genetic stability of previously lyophilised *Neisseria lactamica* carried over 168 days
**Safety endpoints**	i. Occurrence of unsolicited adverse events within the study period<br>ii. Occurrence of serious adverse events within the study period

**Microbiological endpoints**	i.	Assessment of colonisation – culture of N. lactamica from throat swabs taken between day 4 and day 14 post challenge
	ii.	Microbiological assays to characterise kinetics and density of Nlac colonisation from day 4 to day 168 post challenge and at 2 years post challenge in a subgroup of participants
	iii.	Assays to determine genetic stability of Nlac colonising the nasopharynx from day 4 to day 168 post challenge and at 2 years post challenge in a subgroup of participants
**Immunological endpoints**	i.	Rise in serological specific antibody titre comparing day 0 versus day 28 post challenge
	ii.	Rise in mucosal specific antibody titre comparing day of screening versus day 28 post challenge
**Microbial challenge material**	Reconstituted lyophilised Wild type *Neisseria lactamica*– dose 10^3 – 10^7 cfu	

2. Abbreviations

AE	Adverse Event
ALP	Alkaline Phosphatase
ALS	Advanced Life Support
ALT	Alanine transaminase
AR	Adverse Reaction
CFU	Colony Forming Unit
CI	Chief Investigator
CRF	Case Report Form
CRP	C Reactive Protein
ECG	Electrocardiogram
ESC	External Safety Committee
GCP	Good Clinical Practice
GP	General Practitioner
HRA	Health Research Authority
ICH	International Conference on Harmonisation
ILS	Immediate Life Support
MHRA	Medicine and Healthcare products Regulatory Agency
NHS	National Health Service
NIHR	National Institute for Health Research
NIHR HPRU	National Institute for Health Research, Health Protection Research Unit
NIHR-CRF	NIHR Clinical Research Facility
Nlac	*Neisseria lactamica*
Nmen	*Neisseria meningitidis*
PBS	Phosphate Buffered Saline
PCR	Polymerase Chain Reaction

PI	Principal Investigator
QA	Quality assurance
QC	Quality control
REC	Research Ethics Committee
SAE	Serious Adverse Event
SAR	Serious Adverse Reaction
SI	Standard Inoculum
SmPC	Summaries of Product Characteristics
SOP	Standard Operating Procedure
SUSAR	Serious Unexpected Serious Adverse reaction
TOPS	The Over-volunteering Prevention System
UAR	Unexpected Adverse Reaction
UHS NHS FT	University Hospital Southampton NHS Foundation Trust
UK	United Kingdom

3. Background and rationale

3.1 The rationale for the current study

This study is a planned and funded investigation forming part of the National Institute for Health Research, Health Protection Research Unit (NIHR HPRU) in Mucosal Immunology which is based at University College London. One of the planned experimental initiatives is experimental human challenge with *Neisseria lactamica* (Nlac) to be undertaken in Mali, which will investigate mucosal immune responses in participants following inoculation with Nlac. These studies will determine whether there are cross protective responses against *Neisseria meningitidis* (Nmen) and whether, as occurs in UK volunteers, there is an Nlac-induced suppression of meningococcal carriage. We have previously nasally inoculated over 350 volunteers with Nlac, but now seek to modify the method by using reconstituted lyophilised Nlac (lyoNlac).

In this pilot research we will develop and validate a modification of the methodology previously used in our UK-based human challenge experiments. The research to be conducted in this study will inform us whether intranasal inoculation of reconstituted lyophilised Nlac (hereafter, lyoNlac), can result in immunising colonisation of participants and the optimal dose to achieve this. Previous challenges have been conducted using frozen stocks of Nlac but this is relatively unsatisfactory because of instability of frozen stocks, and will not be practical in Mali. The facility to reconstitute dry powdered lyoNlac into water and inoculate as a nose drop will greatly simplify the experimental method. However, we do not know whether lyoNlac can be inoculated directly into volunteers and induce successful colonisation.

In summary this is a pilot study of the safety, efficacy and immunogenicity of the intranasal inoculation of healthy human volunteers with lyoNlac. This study will determine the efficacy of lyoNlac as an agent for inducing nasopharyngeal colonisation with this organism, which is an important methodological development for ease of administration of Nlac in experimental human challenge studies. We will aim to determine the dose of inoculum required to induce colonisation in 80% of volunteers (the Standard Inoculum or SI). This SI will be used in future studies aiming to optimise duration of colonisation and immunogenicity induced by lyoNlac.

3.2 *Neisseria lactamica* and *Neisseria meningitidis*

Nlac and Nmen are Gram negative diplococci which both colonise the human nasopharynx. Nlac is non-pathogenic, non-encapsulated and lactose-fermenting and is a common commensal, particularly in young children. Transmission occurs through close contact and only a few cases of clinical significance have been reported, (Bidmos, Neal et al. 2011) (Denning and Gill 1991) (Brown, Ragge et al. 1987).

Nmen, the causative agent of meningococcal disease, is a human-adapted, often encapsulated species that uses the human nasopharynx as its sole biological niche (Laver, Hughes et al. 2015). In the vast majority of interactions with humans, the organism colonises silently, and disease is extremely rare.

Although Nlac and Nmen colonise the same location within the upper respiratory tract, previous studies suggest they engage with the human mucosal immune system in very different ways (Lauer and Fisher 1976). In contrast to Nmen, Nlac maintains a purely

commensal relationship with the host. Nlac lacks a polysaccharide capsule, so any adaptive immune responses to this bacterium must be directed at non-capsular antigens, providing a good platform for assessing non-anti-polysaccharide immunity against colonising bacteria when compared with wild type Nlac.

3.3 Carriage of *Neisseria spp.*

The highest rate of natural carriage of the harmless commensal Nlac occurs in infants. Carriage wanes in toddlers and older children and by the time a person reaches adolescence, carriage is about 1%.

This differs to the pattern of carriage of Nmen. In the UK, at any given time, an average of 10% of the human population are carrying this organism in the nose and throat (detectable by throat swabbing) but this varies with age. The highest rates of carriage are seen in teenagers and University students. Activities associated with carriage of Nmen include attendance at pubs or clubs, smoking, residence in student halls of residence and saliva exchange, for example kissing (Laver, Hughes et al. 2015). Infants have low rates of carriage, but this increases gradually as childhood progresses. Although carriage of the organism is quite common, disease is extremely rare – currently less than 1 per 100,000 per annum in the UK. Therefore, the likelihood of disease even when a person is carrying a virulent strain of Nmen, is very low (Trotter, Gay et al. 2006).

There is an epidemiological relationship between carriage of the commensal Nlac and meningococcal disease. Age-specific rates of Nmen carriage and disease were inversely proportional to carriage of Nlac (Cartwright, Stuart et al. 1987). The mechanism of this relationship is undetermined, but is not due to cross-protective antibody production, as the early years of life associated with high rates of Nlac carriage predate the development of natural bactericidal meningococcal antibodies (Trotter, Gay et al. 2006). Furthermore, in a controlled infection study, we showed that intranasal inoculation of young adults with live Nlac was followed by development of humoral immunity to Nlac in those who carried the inoculated strain, but this did not induce significant cross-reactive antibodies (Evans, Pratt et al. 2011).

3.4 Immunity and carriage

Colonisation by Nlac is an immunising event; we proved this in humans by inoculating university students intranasally with the harmless commensal Nlac and we observed both specific systemic and mucosal antibody responses by 4 weeks. Likewise a human challenge model has been used to study responses to pneumococci in which, in addition to humoral responses, T cell responses were induced by asymptomatic carriage of wild-type *Streptococcus pneumoniae,* with an increase in IL-17A^{+} Th cells in both the blood and bronchoalveolar lavage fluid by 21-56 days post challenge, suggesting that similar to animal models of carriage, Th17-mediated responses are also mobilised in humans (Wright, Bangert et al. 2013) (Davenport, Guthrie et al. 2003). However, repeated colonisation by the same species is known to occur throughout the lifetime of humans (Glennie, Banda et al. 2012). Experimental challenge with defined bacteria could tease out the mechanisms of this, which include waning of immunity over time, the induction of an incorrectly polarised T cell response, lack of cross-reactivity between strains or active immune evasion mechanisms employed by bacteria to subvert host immune effector mechanisms.

3.5 Vaccine induced immunity to carriage

Asymptomatic oropharyngeal carriage of Nmen is prerequisite for meningococcal disease. Glycoconjugate vaccines have had dramatic effects on disease incidence, at least partly due to herd protection conferred by reduced carriage and transmission, akin to the effect of pneumococcal and *Haemophilus influenzae* type B vaccines. Recent vaccine developments include a new subcapsular vaccine, 4CMenB, which induces bactericidal antibodies against a range of strains, including serogroup B, but we showed in a large prospective randomised study that the effect on carriage of serogroup B *N. meningitidis* is relatively modest (Read, Baxter et al. 2014).

The herd protection conferred by glycoconjugate vaccines is a result of vaccine-induced modification of colonisation reducing inter-host transmission (Maiden et al., 2008: Impact of meningococcal serogroup C conjugate vaccines on carriage and herd immunity. J Infect Dis. 2008 Mar 1;197(5):737-43.) Future successful vaccines will target pathogen colonisation, using antigens known to induce immunity critical for colonisation, in age groups most likely to transmit to others.

3.6 Human challenge with *Neisseria lactamica*

Nlac has been shown to be safe in human challenge as we have found in over 350 volunteers experimentally nasally inoculated with the wild type organism (Evans, Pratt et al. 2011) (Deasy, Guccione et al. 2015). Even at a very low dose of 10^4 colony forming units (CFU), long lasting colonisation with Nlac is easily induced in 35-65% of participants. In 80-90% of those successfully colonised, this is detectable by 1-2 weeks after inoculation. Data regarding earlier detection of colonisation is currently lacking. (Evans, Pratt et al. 2011) (Deasy, Guccione et al. 2015). In a subgroup of volunteers who were not successfully colonised despite two challenges with 10^4 CFU, increasing the inoculum dose to 10^5 CFU resulted in successful colonisation of 50% of the subgroup. (Evans, Pratt 2011) Colonisation has a clear effect on the nasal mucosal microbiome, in that meningococcal acquisition is effectively inhibited in participants who carry the organism (Deasy, Guccione et al. 2015). Colonisation is immunogenic with an increase in specific serum IgG by 2 weeks and specific salivary IgA by 4 weeks (Evans, Pratt et al. 2011).

3.7 Lyophilised *N. lactamica*

In previous human challenge work we have used frozen stocks of *N. lactamica* which are thawed and diluted and then administered to the participants. The disadvantages of this technique are that (a) stocks need to be maintained at -80°C, (ii) there is a gradual decline in viable counts from frozen stocks, (iii) frozen stocks are relatively difficult to transport, and (iv) dilution of frozen stock can be an inaccurate process resulting in lower or higher inocula than planned.

Lyophilisation (freeze drying) is a long-established process in which bacteria are dessicated to form a powder material which allows long term storage with efficient reconstitution to a viable state on re-addition of water or PBS when stocks are needed.

We have shown that our stock of Nlac can be lyophilised, that lyophilised Nlac (lyoNlac) can be reconstituted to yield viable CFU and that lyoNlac retains its viability for at least 3 months following the lyophilisation process. Recovery rates of viable CFU from lyoNlac batches average 50% of the number of viable CFU subjected to freeze-drying. Cryoprotection of mid-to-late log phase Nlac is achieved using a mixture of commercially available soya milk

(Alpro) and commercially available sucrose (10%) (w/v) in PBS, which is used to resuspend a washed bacterial cell pellet prior to freezing. Frozen bacteria are then dried in a strong vacuum overnight to generate a powder that can be stored until needed, and contain defined amounts of reconstitutable bacteria.

LyoNlac is then reconstituted in sterile water to yield cultivable *N. lactamica* which is phenotypically and genetically identical to our inoculum stocks of the bacterium. In the experiments described here, LyoNlac reconstituted in water will be administered to study volunteers by nasal inoculation.

4. Objectives

4.1 Primary objective

To assess the safety of nasal inoculation of healthy volunteers with reconstituted lyophilised wild type *Neisseria lactamica*

4.2 Secondary objectives

i. To establish the dose of nasally administered reconstituted lyophilised *Neisseria lactamica* required to induce nasopharyngeal colonisation in approximately 80% of inoculees
ii. To demonstrate the immunogenicity of colonisation induced by reconstituted lyophilised wild type *Neisseria lactamica*
iii. To examine the kinetics and density of colonisation induced by reconstituted lyophilised wild type *Neisseria lactamica*
iv. To assess the genetic stability of previously lyophilised *Neisseria lactamica* carried over 168 days

5. Description and justification of the study design

5.1 Overview

This is a dose-ranging human challenge study in which participants will be inoculated intranasally with reconstituted lyoNlac (0.5 ml per nostril). This study seeks to determine the minimum dose of inoculum that results in colonisation of approximately 80% of volunteers. A dose ranging strategy will be used, challenging volunteers with reconstituted lyoNlac in cohorts of 5 volunteers per dose. The initial dose will be 10^5 colony-forming units (CFU) and will be escalated or de-escalated by ½-1 $\log_{10}$ depending upon the proportion of volunteers colonised with viable *N. lactamica*. The maximum number of CFU that will be used for challenge is 10^7 CFU. There is no lower limit to the number of CFU that can be used for challenge. Once the minimum number of CFU sufficient to colonise 80% of 5 volunteers is determined, a further 5 volunteers will be challenged with that dose to confirm 70-90% colonisation. This dose will then be defined as the Standard Inoculum (SI). Further volunteers will be challenged with the SI until a total of 10 volunteers have been colonised at that dose as a sample size of 10 is required to show immunogenicity. Safety parameters will

be monitored during each visit. Immunogenicity will be assessed at day 28 in comparison to baseline.

5.2 Study volunteers

Healthy volunteers aged 18-45 years will be recruited and challenged in staggered cohorts of 5. In our previous studies using a liquid suspension of wild type *N. lactamica* administered intranasally, colonisation was achieved in 35% of *N. lactamica* -inoculated non-smokers at a dose of 10^4 CFU, (Deasy, Guccione et al. 2015) and increasing the inoculum to 10^5 CFU increased the subsequent carriage of *N. lactamica* to 50% of volunteers (Evans, Pratt et al. 2011). We estimate that the total number of volunteers required will be in the range 15-35.

5.3 Challenge procedure

Our priority is to conduct this study without causing harm to the volunteer. As *N. lactamica* is a non-virulent commensal organism and based on previous challenge studies with this organism, we consider that the likelihood of disease resulting from inoculation of the volunteers is so low as to be considered negligible.

5.4 First volunteers – safety

For each cohort receiving an escalated dose the first volunteer will be challenged individually. A safety review will be carried out on or after day 4 and the safety report will be signed by the PI or CI. Providing there are no safety concerns the remaining volunteers in the cohort will be challenged in groups up to three with a safety review on or after day 4 following each challenge.

For dose de-escalation or a second cohort of volunteers to receive a given dose, volunteers will be challenged in groups of a maximum of five.

If eradication therapy or treatment is given to any volunteer due to safety concerns, the next volunteer will be challenged individually. If two or more volunteers out of a group of five receive treatment due to safety concerns, no new volunteers will be challenged until the data have been reviewed by the external safety committee and study continuation approved.

5.5 Assessment of colonisation

Colonisation is defined as the culture of viable *N. lactamica* from a throat swab or nasal wash taken on or before day 14 post inoculation.

5.6 Dose ranging strategy

Volunteers will be challenged in in cohorts of 5 volunteers with a specified dose. The dose will then be escalated, de-escalated or unchanged for the following cohort of 5 depending on the number of volunteers colonised, as shown in Figures 5.1 and 5.2 below. This process will be repeated until the dose inducing approximately 80% colonisation has been identified. Once colonisation has been induced in 7-9 out of a total of 10 volunteers at a given dose, this dose will be defined as the standard inoculum (SI). Further volunteers will then be challenged with the SI until a total of 10 volunteers have been colonised with this dose.

The initial dose will be 10^5 CFU and the maximum dose used will be 10^7 CFU. If 70-90% colonisation is not achieved with 10^7 CFU then this dose may be used as the SI for future studies, accepting a lower colonisation rate.

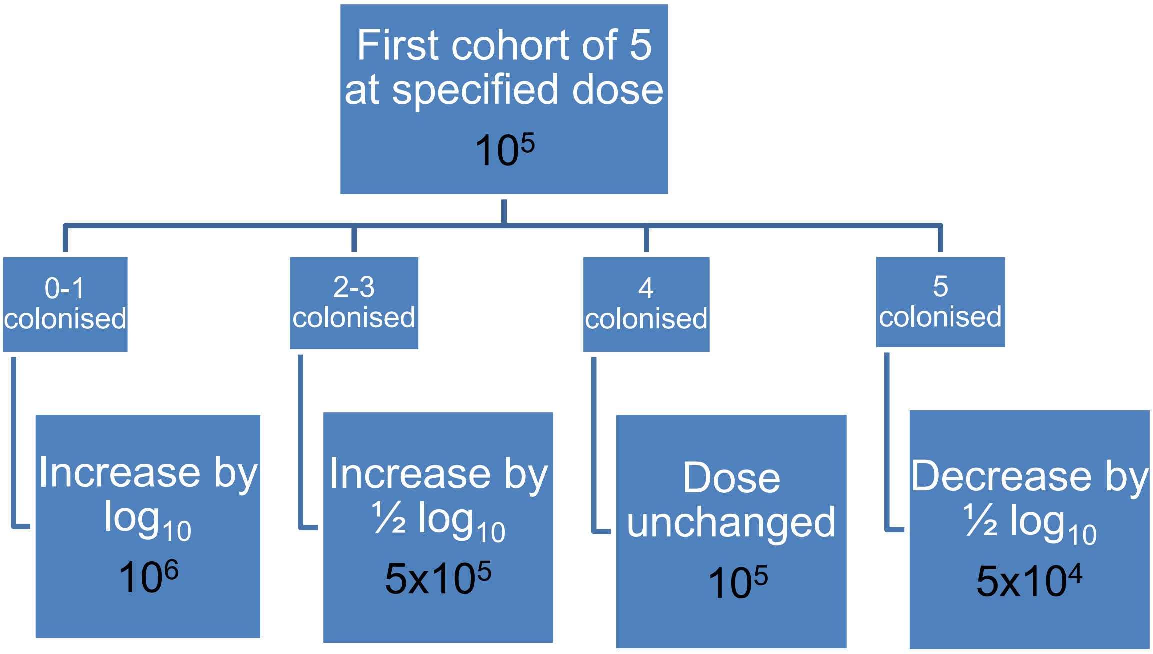

Figure 5.1: Dose ranging strategy for first cohort challenged with a specified dose. The doses for the first cohort in the study are given as an example.

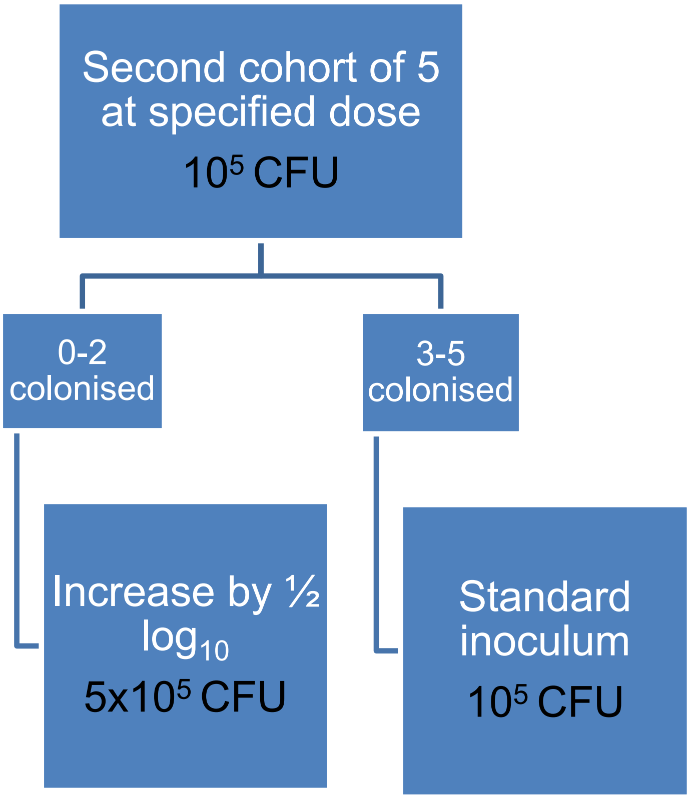

Figure 5.2: Dose ranging strategy for the second cohort challenged with a specified dose. The doses for the first cohort in the study are given as an example.

5.7 Duration of volunteer participation

The duration of involvement of volunteers in the study will be from the screening visit (up to 30 days prior to inoculation) until 168 days from inoculation. For volunteers who choose to participate in the optional 2 year substudy, they will have a final visit 18 months to 3 years years after the initial challenge.

5.8 Definition of the start and the end of the study

The start of the study is defined as the date of screening of the first volunteer. The end of the study is defined as 12 months after the date of the last visit of the last volunteer to allow for sample processing and data analysis.

5.9 Potential benefits for the volunteers.

Volunteers will not benefit directly from participation in this study. However, it is hoped that the information gained from this study will contribute to knowledge about nasopharyngeal colonisation and therefore to the development of safe and effective vaccines in the future. Volunteers will also receive information about their general health status.

5.10 Involvement of the NHS Research Ethics Committee

This study protocol has been submitted to Oxford A Regional Ethics Committee (REC) for approval of this human challenge study.

6. Inoculum

6.1 Selection of strain of *N. lactamica*

A wild-type strain of *Neisseria lactamica* strain Y92-1009 (sequence type 3493, clonal complex 613) will be used because we have previously used it safely in experimental challenge of over 350 human volunteers (Deasy, Guccione et al. 2015).

6.2. Supply and storage of the stock strain

Stocks of *N. lactamica* Y92-1009 (sequence type 3493, clonal complex [CC] 613) in Frantz medium containing 30% (v/v) glycerol were supplied by the Current Good Manufacturing Practices pharmaceutical manufacturing facilities at Public Health England (Porton Down, United Kingdom) as vials of $1x10^6$ bacteria, suspended in Frantz medium containing 30% (v/v) glycerol, transported and stored at -80°C. These stocks were transferred to the University Hospital Southampton under temperature-monitored conditions and are stored at -80°C in a locked, dedicated, temperature monitored freezer.

6.3 Lyophilisation of *N. lactamica*

The lyophilised stock will be prepared in the Medical School laboratory LC70 at the University of Southampton by Dr Jay Laver, following the standard operating procedure "**Production, storage and monitoring of lyophilised *N. lactamica* inoculum for human challenge studies".** A chain of accountability will be recorded at a GMP-like standard.

6.4 Preparation of nasal inoculum

The nasal inoculum will be prepared for inoculation by reconstituting lyoNlac in sterile water following the SOP: **Preparation and monitoring of lyoNlac for nasal inoculation**

6.5 The optimal and safe dose of the inoculum

In previous studies using nasal inoculation with liquid suspensions of wild type *N. lactamica*, colonisation was achieved in 35% of *N. lactamica* -inoculated non-smokers at a dose of 10^4 CFU, (Deasy, Guccione et al. 2015) and increasing the inoculum to 10^5 CFU increased the subsequent carriage of *N. lactamica* to 50% of volunteers (Evans, Pratt et al. 2011). There have been no safety concerns with inoculation at these doses. We will start at a dose of 10^5 and escalate or de-escalate the dose aiming for the minimum dose required to achieve 80% colonisation.

6.6 Monitoring of the *N. lactamica* dose administered to volunteers

After the inoculum is given a sample of the residual inoculum will be diluted and cultured overnight. The dose will then be evaluated by viable count and the species confirmed.

7. Recruitment and withdrawal of trial volunteers

7.1 Recruitment

Healthy volunteers will be recruited through various media. Care will be taken not to recruit from vulnerable groups (mental health or other capacity issues or those under 18 years old). The recruitment strategy will be approved by the Health Research Authority (HRA). Volunteers may be recruited by use of an advertisement +/- registration form formally approved by the ethics committee and distributed or posted in the following places:

- In public places, including buses and trains, with the agreement of the owner / proprietor
- In newspapers or other literature for circulation
- On radio via announcements
- On a website operated by our group or with the agreement of the owner or operator (including on-line recruitment through our web-site)
- As a post on a Twitter, Facebook or Gumtree account owned and operated by our group
- Video message posted on the National Health Service (NHS) YouTube channel.
- By e-mail distribution to a group or list only with the express agreement of the network administrator or with equivalent authorisation
- By email distribution to individuals who have already expressed an interest in taking part in any clinical trial at the NIHR-CRF Southampton
- On stalls or stands at exhibitions or fairs
- Via presentations (e.g. presentations at lectures or invited seminars)
- Direct mail-out to individuals who have requested information about participation in research studies

- Southampton NIHR-CRF Database of Healthy Volunteers: We may contact individuals from this database who have previously expressed an interest in receiving information about future studies for which they may be eligible

7.2 Volunteer information sheet

A volunteer information sheet will be given to the volunteer at least 24 hours before the screening visit. The volunteer information sheet will include all risks of participating in this study and safety measures that are involved in the study and will be formally approved as part of the REC and HRA application.

7.3 Screening visit

Individuals who have expressed an interest in taking part in the study will be invited to attend a screening session after a short telephone screening. During the screening visit the study will be explained. If the volunteer has any questions they can be addressed during this visit. The screening visit can be up to 30 days prior to challenge on day 0.

7.3.1 Informed consent

All volunteers will sign and date the informed consent form before any study specific procedures are performed. At the screening visit, the volunteer will be fully informed of all aspects of the trial, the potential risks and their obligations. The following will be emphasised:

- Participation in the study is entirely voluntary
- Refusal to participate involves no penalty or loss of medical benefits
- The volunteer may withdraw from the study at any time
- The volunteer is free to ask questions at any time to allow him or her to understand the purpose of the study and the procedures involved
- There is no direct benefit from participating
- The volunteer will be registered on the TOPS database (The Over-volunteering Prevention System; www.tops.org.uk)
- The aims of the study and all tests to be carried out will be explained. The volunteer will be given the opportunity to ask about details of the trial, and will then have time to consider whether or not to participate

The volunteer will be asked to sign and date two copies of the consent form, which will also be signed and dated by the investigator. One original will be given to the volunteer and the other will be stored in the NIHR-CRF. A copy will be stored in the volunteer's medical file.

7.3.2 Medical history and physical examination

A detailed medical history and physical examination including height, weight and vital signs will be conducted, making sure that all inclusion criteria and no exclusion criteria are met.

7.3.3 Screening investigations

A throat swab will be taken to look for *N. lactamica* and *N. meningitidis* carriage at screening. Females will have a pregnancy test. A nasal wash will be taken according to the SOP:

Collection of a nasal wash sample for culture and as a baseline for assessing mucosal immunogenicity. Screening blood tests and an ECG will be performed at screening.

The following reference ranges are provided for the purpose of guidance only. Results that fall outside of these ranges may not be of clinical significance but should be considered on an individual basis and may be repeated once prior to confirming eligibility.

	Lower limit	Upper limit
**Haematology**		
Haemoglobin [g/L]	Male: 130<br>Female: 120	Male: 170<br>Female: 150
White Cell Count [x 10^9/L]	4	11
Platelet Count [x 10^9/L]	150	450
Neutrophil count [x 10^9/L]	2	7.5
Lymphocyte count [x 10^9/L]	1.5	4.0
**Biochemistry**		
Sodium [mmol/L]	133	146
Potassium [mmol/L]	3.5	5.3
Urea [mmol/L]		7.8
Creatinine [µmol/L]		97
Albumin [g/L]	35	
Total bilirubin [µmol/L]		20
ALT [IU/L]		Male: 50<br>Female: 43
ALP [IU/L]		130
C reactive protein [mg/l]		10
Pregnancy test	Positive	
**ECG**		
QTc	Male: ≥440 ms.<br>Female: ≥460 ms	

Table 7.1 Lower and upper values of normal in blood and urine tests are based on normal values Southampton University Hospital NHS Foundation Trust Clinical Laboratory

7.4 Inclusion and exclusion criteria

7.4.1 Inclusion criteria

The volunteer must satisfy all the following inclusion criteria to be eligible for the study:

- Healthy adults aged 18 to 45 years inclusive on the day of enrolment
- Fully conversant in the English language
- Able and willing (in the investigator’s opinion) to comply with all study requirements
- Provide written informed consent to participate in the trial
- For females only, willingness to practice continuous effective contraception (see below) during the study and a negative pregnancy test on the day(s) of screening and inoculation
- TOPS registration completed and no conflict found

7.4.2 Exclusion criteria

The volunteer may not enter the study if any of the following criteria apply:

- Current active smokers defined as having smoked a cigarette or cigar in the last four weeks
- Individuals who have a current infection at the time of inoculation
- Individuals who have been involved in other clinical trials involving receipt of an investigational product over the last 12 weeks or if there is planned use of an investigational product during the study period
- Individuals who have previously been involved in clinical trials investigating meningococcal vaccines or experimental challenge with *N. lactamica*
- Use of systemic antibiotics within the period 30 days prior to the challenge
- Any confirmed or suspected immunosuppressive or immune-deficient state, including HIV infection; asplenia; recurrent, severe infections and chronic (more than 14 days) immunosuppressant medication within the past 6 months (topical steroids are allowed)
- Use of immunoglobulins or blood products within 3 months prior to enrolment.
- History of allergic disease or reactions likely to be exacerbated by any component of the inoculum, specifically soya.
- Contraindications to the use of ciprofloxacin, specifically a history of epilepsy, prolonged QT interval, hypersensitivity to quinolones or a history of tendon disorders related to quinolone use
- Any clinically significant abnormal finding on clinical examination or screening investigations
- Any other significant disease, disorder, or finding which may significantly increase the risk to the volunteer because of participation in the study, affect the ability of the volunteer to participate in the study or impair interpretation of the study data, for example recent surgery to the nasopharynx
- Occupational, household or intimate contact with immunosuppressed persons
- Pregnancy or lactation

7.4.3 Effective contraception for female volunteers

Female volunteers are required to use an effective form of contraception during this study. Acceptable forms of contraception include:

- Established use of oral, injected or implanted hormonal methods of contraception
- Placement of an intrauterine device or intrauterine system
- Total abdominal hysterectomy
- Barrier methods of contraception (condom or occlusive cap with spermicide)

- Male sterilisation if the vasectomised partner is the sole partner for the subject
- True abstinence when this is in line with the preferred and usual lifestyle of the subject

8. *N. lactamica* challenge (Day 0)

8.1 Trial site

The challenge will take place in the NIHR Clinical Research Facility at University Hospital Southampton NHS Foundation Trust (UHS NHS FT). (http://www.uhs.nhs.uk/ClinicalResearchinSouthampton/Trials-and-facilities/NIHRWellcome-Trust-Clinical-Research-Facility/Our-facility.aspx).

8.2 Clinical team involved in the challenge

The challenge will be conducted by the study doctor together with a study nurse. The study doctor will be responsible for the administration of the inoculum. Advanced Life Support (ALS) trained doctors and Immediate Life Support (ILS) trained nurses will be present at inoculation and available within the NIHR-CRF for the post inoculation period of observation. At least 2 healthcare professionals will be available within the NIHR-CRF and a research clinician will be contactable by telephone whenever volunteers are present on the unit for follow up visits. The NIHR-CRF is situated in the University Hospital Southampton NHS Foundation Trust and a resuscitation team and intensive care facilities are available. Volunteers will have a 24 hours 7 days per week contact number to contact the Principal Investigator (PI) and research team in case of any adverse reactions during the study.

8.3 Infection control

The research team will adhere to the University Hospital Southampton Standard Infection Prevention and Control Precautions (Version 5 25/4/2016) for all patient contact and procedures including the challenge procedure.

8.4 Challenge Procedures

8.4.1 Confirmation of identity of the volunteer

Before any procedure is performed the identity of the volunteer will be confirmed by asking his/her name and date of birth and comparing it to the case report form and the label on the inoculum.

8.4.2 Review prior to the challenge

Prior to any procedure taking place the volunteer will be asked if he has any questions about the study and agrees to continue with the study. Eligibility will be reconfirmed before the challenge is conducted.

Medical history

The study doctor will take an interim medical history asking if there are any new medical issues since the screening visit. Special attention will be given to any possible signs of infection such as fever. If there are any significant abnormalities found, the challenge will be postponed at the discretion of the study doctor.

Physical examination
Vital signs including heart rate, blood pressure, respiratory rate and temperature will be recorded prior to the challenge procedure. Physical examination will be performed if clinically indicated. If there are any significant abnormalities found, the challenge may be postponed at the discretion of the study doctor.

Laboratory investigations
A throat swab will be taken for culture and blood samples will be taken as a baseline for the assessment of cellular and humoral immunity. A saliva sample will be collected according to the SOP: **Collection of saliva using an Oracol saliva swab**. Collection of all clinical samples will adhere to the UHS Standard Infection Control Precautions Policy (Version 5.0, 25/4/16).

8.4.3 Challenge

Members of the study team present at the challenge
The challenge will be conducted by the study doctor with a study nurse present.

Location of the challenge
The challenge will take place in the NIHR-CRF.

Time schedule of the challenge
The inoculation will take about 15 minutes, after which the volunteer will remain in the NIHR-CRF for further observation for a total of 30 minutes.

Preparing the inoculum
The inoculum will be prepared in the NIHR-CRF laboratory using a dedicated category II safety hood by technical staff. Two people will be present during preparation: one team member will prepare the inoculum, while the other team member will check the procedure which will be carried out according to the SOP: **Preparation and monitoring of lyoNlac for nasal inoculation**.

Administering the inoculum
The challenge procedure will be carried out by one of the study doctors according to the SOP: **Performance of nasal inoculation for human challenge studies**.

9. Follow up visits

9.1 Main study

Volunteers will attend for follow up visits as detailed in table 9. At each visit the volunteers will be assessed for local and systemic adverse events and concomitant medication use. Measurement of vital signs and physical examination will be performed if clinically indicated and laboratory samples will be taken at the time-points indicated in the schedule of attendances. An appointment will be made for the next follow up visit.

	Screening	Challenge		Follow up				
Timeline (days)	≤ 30	0	4	7	14	28	42	168
Visit window			0	+/-1	+/- 2	+/- 3	+/-3	+/-7
TOPS confirmation	+							
Volunteer Information Sheet	+							
Informed consent	+							
Vital signs	+	+	(+)	(+)	(+)	(+)	(+)	(+)
Medical history	+							
Physical examination	+	(+)	(+)	(+)	(+)	(+)	(+)	(+)
Pregnancy test (females only)	+	+						
Electrocardiogram	+							
Review eligibility		+						
Challenge		+						
Review of adverse events and concomitant medications		+	+	+	+	+	+	+
Nasal wash	+		+	+	+	+	+	+
Throat swab (microbiology)	+	+	+	+	+	+	+	+
Saliva sample		+				+		
Safety bloods (ml)	8		8					
Immunological blood tests (ml)		20*				20*		
Cumulative blood volume (ml)		28*	36*			56*		

Table 9: Summary of monitoring procedures during the study – (+) if clinically indicated, *Up to maximum volume of.

9.2 Optional 2 year sub-study

Volunteers who have participated in the main part of the study will be invited to attend a single additional visit to look for long term colonisation with *N. lactamica*. It will be highlighted that this is an optional visit. The window for this visit will be 18 months to 3 years post-inoculation.

Volunteers who agree to participate in this sub-study will attend a further visit at which they will be given an opportunity to discuss their involvement with a member of the study team and then complete a 2 year sub-study informed consent form.

Information will be collected about their general health and any significant medical events since the Day 168 visit. This information will be recorded but not reported as AEs/SAEs unless they are assessed as being related to the study procedures.

Vital signs will be recorded. A physical examination will be carried out if this is felt to be required by the study team.

A throat swab and nasal wash will be obtained to look for ongoing colonisation with *N. lactamica*.

10. Clinical and laboratory monitoring

10.1 Clinical team

The clinical team involved in the monitoring of the volunteer will consist of the clinical investigators, NIHR-CRF research fellows and NIHR-CRF research nurses. At least two ILS or ALS trained staff members will be present on the NIHR-CRF throughout the period of observation following the challenge procedure. The NIHR-CRF is treated as a hospital ward and so is covered by the hospital emergency response teams and medical intensive care support 24 hours per day.

10.2 Monitoring of volunteers

After the challenge the volunteers will be monitored in the NIHR-CRF for 30 minutes. They will have their vital signs measured at the end of this period of observation. A 24/7 emergency telephone contact number for the research team will be available in case of any concerns or systemic symptoms. The investigator will consider an extra clinical review if the volunteer has any symptoms that are moderate or severe.

10.3 Management of possible *N. lactamica* disease

In the event of development of fever, any symptoms or abnormal safety bloods the volunteer will be reviewed by the study doctor. If required then safety bloods will be taken including a blood culture sent through the routine hospital microbiology service.

If no immediate treatment is indicated then the symptoms will be investigated as appropriate for patients with undefined infections.

Potential *N. lactamica* disease affecting the respiratory tract only with a low clinical likelihood of bacteraemia will be treated / eradicated using ciprofloxacin 500 mg daily for 7 days, and the volunteer will be referred to their General Practitioner. Ciprofloxacin proved effective at eradicating *N. meningitidis* (Deasy, Guccione et al. 2015) and should eradicate *N. lactamica* carriage within 24 hours (Fraser, Gafter-Gvili et al. 2006). The strain used is fully sensitive to this antibiotic with an exceptionally low MIC on E-testing. The first dose will be taken under supervision of the study team (directly observed treatment).

If the doctor makes a clinical diagnosis of sepsis and considers that this may require immediate treatment, the volunteer will be referred to the acute medical services of the hospital (located within the same building). In the event of any suspicion of bacteraemia due to GM *N. lactamica* it is likely that the acute medical services will administer ceftriaxone and manage in a similar way to meningococcal disease.

In the unlikely event of volunteers experiencing severe illness during the period of the study, there is a 24/7 intensive care service available at the University Hospital of Southampton NHS Foundation Trust. In case of resuscitation of the volunteer being required, a 24/7 resuscitation service is available in the NIHR Clinical Research Facility, which is considered a hospital ward for resuscitation purposes and which is located in the centre of the University Hospital of Southampton.

10.4 Potential adverse events

10.4.1 Phlebotomy

The maximum volume of blood drawn over the study period (56 mls) should not compromise these otherwise healthy volunteers. There may be minor bruising, local tenderness or pre-syncopal symptoms associated with venepuncture, which will not be documented as Adverse Events (AEs) if they occur.

10.4.2 Inoculation with N. lactamica

The inoculation with 0.5 millilitres of *N. lactamica* suspension per nostril can cause some irritation of the nasal mucosa that will disappear within a few seconds. Very occasionally, instillation may induce coughing or sneezing, but this can be prevented by slow instillation down the superior wall of the nares.

10.4.3 Throat swab, saliva sample and nasal wash

The nasal samples taken by throat swab and nasal wash can cause some irritation of the nasal mucosa and can induce coughing or sneezing. This nasal discomfort will disappear within a few minutes and will not be recorded as an AE. The collection of saliva samples is not expected to cause any discomfort.

10.4.4 N. lactamica disease

We have inoculated over 350 individuals in the past with wild type *N. lactamica* and no serious adverse events have occurred. In the unlikely event that the lyophilised *N. lactamica* causes disease we would anticipate that this would be either due to involvement of the respiratory tract (which would be signalled by fever and cough) or due to invasion of the bloodstream (which would be signalled by fever and features of sepsis akin to meningococcal disease). Suspected or confirmed *N. lactamica* disease will be recorded as an AE,

10.4.5 Eradication therapy

If *N. lactamica* disease is suspected, ciprofloxacin eradication therapy may be used. The side effects of ciprofloxacin in young adults may include:

- Abdominal ache, diarrhoea and nausea.
- Tiredness and headaches.
- Rash and itching.
- Facial swelling - very rarely breathing difficulties may occur with the facial swelling.
- Pain and inflammation around the joints

Volunteers should contact the clinical study team if this occurs.

Female volunteers using the oral contraceptive pill will be advised to use alternative forms of contraception for two weeks following eradication therapy as ciprofloxacin may interfere with the oral contraceptive pill.

10.5 Withdrawal of volunteers

In accordance with the principles of the current revision of the Declaration of Helsinki (updated 2008) and any other applicable regulations, a volunteer has the right to withdraw from the study at any time and for any reason, and is not obliged to give his reasons for

doing so. In addition the volunteer may withdraw/be withdrawn from further study procedures at any time in the interests of the volunteer's health and well-being, or for any of the following reasons:

- Administrative decision by the Investigator.
- Ineligibility (either arising during the study or retrospectively, having been overlooked at screening).
- Significant protocol deviation.
- Volunteer non-compliance with study requirements.
- An AE, which requires discontinuation of the study involvement or results in inability to continue to comply with study procedures.
- The reason for withdrawal from further study procedures will be recorded in the Case Report Form (CRF). Except in case of complete consent withdrawal, long-term safety data collection will be continued. For all AEs, appropriate follow-up visits or medical care will be arranged, with the agreement of the volunteer, until the AE has resolved, stabilised or a non-trial related causality has been assigned. Any volunteer who withdrew consent or is withdrawn from further study procedures may be replaced.
- If a volunteer withdraws from the study, blood samples collected before their withdrawal from the trial will be used/stored unless the volunteer specifically requests otherwise. Data from volunteers withdrawn from the study will be included in the analysis of results relating to the study's primary objective.
- In all cases of subject withdrawal, excepting those of complete consent withdrawal, long-term safety data collection will continue as appropriate if subjects have received the inoculum.

11 Laboratory procedures

11.1 Laboratory work

Standard operating procedures for all laboratory work will be followed. Investigators will conform to well established laboratory safety standards.

11.2 Processing and storage of samples

Blood samples

Blood samples for immunological assays will be processed in the NIHR-CRF lab and frozen at -80ºC. Immunological assays (serology and cellular immunology specific to *N. lactamica*) will be conducted according to the procedures established in the test laboratories.

Safety blood samples will be labelled with the participant's hospital number and sent to the University Hospital Southampton's clinical laboratory for processing.

Throat swabs, nasal washes and saliva samples

Saliva samples will be processed in the NIHR-CRF lab and frozen at -80ºC and then analysed according to the procedures established in the test laboratories. Throat swabs and nasal wash samples will be processed and cultured in the NIHR-CRF laboratory with further microbiological analysis in the University of Southampton laboratory. Individual *Neisseria lactamica* colonies will be selected for storage in glycerol medium and frozen to -80ºC to await PCR and sequencing for exact characterisation. The remainder of processed throat swab and nasal wash samples will be frozen at -80ºC for a RT PCR assay which will be conducted according to procedures established in the test laboratory.

11.3 Labelling of samples

Samples will be clearly identified with the study code, the volunteer's unique anonymised identifier, sample ID and time point, and be recorded in the study log. Samples will not be labelled with any personal identifiable information.

11.4 Microbiological sampling

Estimates of population density will be measured at each sampling point. Positive colonisation will be defined as the culture of at least one isolate on or before day 14 after inoculation (Deasy, Guccione et al. 2015). The duration of colonisation will be recorded.

11.5 Immunological analyses

N. lactamica specific IgG will be measured using previously described methods. Nasal washes will be analysed for *N.lactamica* specific IgA and IgG. Saliva samples will be analysed for specific IgA. Other potentially relevant immunological or microbiological assays may be performed on stored samples at the discretion of the investigator.

11.6 Residual clinical samples

Any remaining biological samples will be transferred to a Human Tissue Authority (HTA) licensed tissue bank (the Southampton Research Biorepository) operating under the Faculty of Medicine's HTA licence) at the end of the study for use in future ethically approved studies. A copy of the informed consent form containing personal identifiable information will be stored by the HTA licensed tissue bank but will only be accessible by authorised individuals.

12. Assessment of safety

Safety of the volunteers will be assessed by analysing the frequency, incidence and nature of adverse events and serious adverse events arising during the study.

12.1 Definitions

Adverse Event (AE)

An AE is any untoward medical occurrence in a volunteer, including a dosing error, which may occur during or after administration of the inoculum and does not necessarily have a causal relationship with the intervention. An AE can therefore be any unfavourable and unintended sign (including an abnormal laboratory finding), symptom or disease temporally associated with the study intervention, whether or not considered related to the study intervention.

Adverse Reaction (AR)

An AR is any untoward or unintended response to the inoculum. This means that a causal relationship between the inoculum and an AE is at least a reasonable possibility, i.e., the relationship cannot be ruled out. All cases judged by either the reporting medical investigator or the sponsors as having a reasonable suspected causal relationship to the inoculum (i.e. possibly, probably or definitely related to the inoculum) will qualify as adverse reactions.

Unexpected Adverse Reaction (UAR)

An adverse reaction, the nature or severity of which is not consistent with the applicable information about the inoculum in the protocol, is considered as an unexpected adverse reaction.

Serious Adverse Event (SAE)

An SAE is an AE that results in any of the following outcomes, whether or not considered related to the study intervention.

- Death (i.e., results in death from any cause at any time)
- Life-threatening event (i.e., the volunteer was, in the view of the investigator, at immediate risk of death from the event that occurred). This does not include an AE that, if it occurred in a more serious form, might have caused death.
- Persistent or significant disability or incapacity (i.e. substantial disruption of one's ability to carry out normal life functions).
- Hospitalisation other than admission in the NIHR-CRF, regardless of length of stay, even if it is a precautionary measure for continued observation. Hospitalisation (including inpatient or outpatient hospitalisation for an elective procedure) for a pre-existing condition that has not worsened unexpectedly does not constitute a serious AE.
- An important medical event (that may not cause death, be life threatening, or require hospitalisation) that may, based upon appropriate medical judgment, jeopardise the volunteer and/or require medical or surgical intervention to prevent one of the outcomes listed above. Examples of such medical events include allergic reaction requiring intensive treatment in an emergency department or clinic, blood dyscrasias, or convulsions that do not result in inpatient hospitalisation.
- Congenital anomaly or birth defect.

Serious Adverse Reaction (SAR)

An adverse event (expected or unexpected) that is both serious and, in the opinion of the reporting investigator or sponsors, believed to be possibly, probably or definitely due to the inoculum or any other study treatments, based on the information provided in the protocol.

Suspected Unexpected Serious Adverse Reactions (SUSARs)

A SUSAR is a SAE that is unexpected and thought to be possibly, probably or definitely related to the inoculum.

12.2 Causality assessment

For each AE, an assessment of the relationship of the AE to the study intervention(s) will be undertaken. The relationship of the adverse event with the study procedures will be categorised as unrelated, unlikely to be related, possibly related, probably related or definitely related (Table 12.1). An intervention-related AE refers to an AE for which there is a possible, probable or definite relationship to the study intervention. The investigator will use clinical judgment to determine the relationship. Alternative causes of the AE, such as the natural history of pre-existing medical conditions, concomitant therapy, other risk factors and the temporal relationship of the event to the challenge will be considered and investigated.

0	**No Relationship**	No temporal relationship to the challenge ***and***<br>Alternate aetiology (clinical state, environmental or other interventions); ***and***<br>Does not follow known pattern of response to *N.lactamica*
1	**Unlikely**	Unlikely temporal relationship to the challenge ***and***<br>Alternate aetiology likely (clinical state, environmental or other interventions) ***and***<br>Does not follow known pattern of response to *N.lactamica*
2	**Possible**	Reasonable temporal relationship to the challenge; ***or***<br>Event not readily produced by clinical state, environmental or other interventions; ***or***<br>Follows expected pattern of response to *N.lactamica*
3	**Probable**	Reasonable temporal relationship to the challenge; ***and***<br>Event not readily produced by clinical state, environment, or other interventions ***or***<br>Follows expected pattern of response to *N.lactamica*
4	**Definite**	Reasonable temporal relationship to the challenge; ***and***<br>Event not readily produced by clinical state, environment, or other interventions; ***and***<br>Follows expected pattern of response to *N.lactamica*

Table 12.1: Guidelines for assessing the relationship of an AE to inoculation with *N. lactamica*

12.3 Reporting procedures for AEs

If an adverse event occurs in this research project it will first be reported to the on duty clinical research fellow or research physician, who will investigate and document it in the CRF and medical notes. If the AE is considered to be related and/or serious, a report will be written and sent to the chief and principal investigators (Professor R.C. Read, and Professor S.N. Faust) who will review the report and inform the Chair (or nominated alternate committee member) of the external safety committee. The Sponsor and HRA will be informed if the AE is assessed by the investigators or the external safety committee as having potential to cause harm to the volunteer or subsequent volunteers.

AEs that result in a volunteer's withdrawal from the study or that are present at the end of the study will be followed up (if volunteer consents to this) until a satisfactory resolution or stabilisation occurs, or until a non-study related causality is assigned.

12.3.1 Severity grading of clinical adverse events

The severity of clinical adverse events will be assessed according to the scales in table 12.2. Vital signs and laboratory adverse events will be assessed according to the SOP: **Assessment of laboratory and clinical adverse events for *N. lactamica* human challenge studies.**

**GRADE 0**	None
**GRADE 1**	Mild: Transient or mild discomfort (< 48 hours); no medical intervention/therapy required
**GRADE 2**	Moderate: Mild to moderate limitation in activity - some assistance may be needed; no or minimal medical intervention/therapy required
**GRADE 3**	Severe: Marked limitation in activity, some assistance usually required; medical intervention/therapy required, hospitalisation possible

Table 12.2: Severity grading criterion for AEs.

12.3.2 Reporting procedures for serious AEs (SAEs)

In order to comply with current regulations on serious adverse event reporting to regulatory authorities, the event will be documented accurately and notification deadlines respected. SAEs will be reported to the Principal Investigator immediately when the study team is aware of their occurrence, as described in the relevant SOP. The external safety committee will be notified of SAEs deemed possibly, probably or definitely related to study interventions; the sponsor will be notified immediately (within 24 hours) when the investigators are aware of their occurrence. SAEs will not normally be reported to the ethical committee(s) unless there is a clinically important increase in occurrence rate, an unexpected outcome, or a new event that is likely to affect safety of trial volunteers, at the discretion of the Chief Investigator. In addition to the expedited reporting above, the investigator shall include all SAEs in the annual Development Safety Update Report (DSUR) report.

12.3.3 Reporting procedures for SUSARs

The chief investigator will report all SUSARs to the ethical committee(s) within required timelines. The chief investigator will also inform all investigators concerned of relevant information about SUSARs that could adversely affect the safety of participants. In addition, the chief investigator will report any SUSARs relating to licensed products used in the trial (ciprofloxacin) to the Medicine and Healthcare products Regulatory Agency (MHRA) using the electronic ‘Yellow Card’ System.

All SUSARs and deaths occurring during the study will be reported to the sponsor. For all deaths, any autopsy reports and relevant medical reports will be made available for reporting to the relevant authorities.

12.3.4 Procedures to be followed in the event of abnormal findings

Abnormal clinical findings from medical history, examination or blood tests, will be assessed as to their clinical significance. If a test is deemed clinically significant, it may be repeated, to ensure it is not a single occurrence. If a test remains clinically significant, the volunteer will be informed and appropriate medical care arranged as appropriate with the permission of

the volunteer. Decisions to exclude the volunteer from enrolling in the trial or to withdraw a volunteer from the trial will be at the discretion of the Investigator.

12.3.5 Foreseeable medical occurrences

The following medical occurrences are foreseeable:

- Local sensation effects in the nose following inoculation.
- Local bruises following venesection

12.3.6 Adverse events of special interest

Adverse events of special interest will be reported as SAEs. These are:

- Severe hypersensitivity reactions to the inoculum (e.g. anaphylaxis)
- Overdosing of the inoculum

12.4 Safety profile review

The safety profile will be assessed on an on-going basis by the investigators.

12.5 Study committees

12.5.1 External Safety Committee (ESC)

An external safety committee will be appointed prior to recruitment.

The role of the ESC is to provide overall supervision for the trial and provide advice through its independent Chair. The ultimate decision for the continuation of the trial lies with the Chief investigator following advice from the ESC.

The ESC will be responsible for reviewing and assessing this protocol prior to commencement of the trial, recruitment, interim monitoring of safety and effectiveness, trial conduct and external data. The ESC will first convene prior to trial initiation and will then define the frequency of subsequent meetings (at least annually).

All correspondence between investigator and ESC will be conveyed by the investigator to the trial Sponsor. The study protocol and implemented safety procedures will be discussed with the ESC before starting the study.

The chair of the ESC may be contacted for advice and independent review by the investigator or trial Sponsor in the following situations:

- Following any SAE deemed to be possibly, probably, or definitely related to a study intervention.
- Any other situation where the Investigator or trial sponsor feels independent advice or review is important.

12.5.2 Ethics Committee

The study will be reviewed by the HRA. A progress report will be submitted to the REC 12 months after the date on which the favourable opinion on which the form was given.

13. Analysis and Statistical considerations

13.1 Endpoints of the study

13.1.1 Safety endpoints

1. Occurrence of unsolicited adverse events within the study period
2. Occurrence of serious adverse events within the study period

Safety analysis will be carried out for all volunteers that received the inoculum, regardless of whether or not they complete the study

13.1.2 Microbiological endpoints

1. Assessment of colonisation – culture of N. lactamica from throat swabs taken between day 4 and day 14 post challenge
2. Microbiological assays to characterise kinetics and density of Nlac colonisation from day 4 to day 168 post challenge and at 2 years post challenge in a subgroup of participants
3. Assays to determine genetic stability of Nlac colonising the nasopharynx from day 4 to day 168 post challenge and at 2 years post challenge in a subgroup of participants

13.1.3 Immunological endpoints

1. Rise in serological specific antibody titre comparing day 0 versus day 28 post challenge
2. Rise in mucosal specific antibody titre comparing day of screening versus day 28 post challenge.

13.2 Sample size

Volunteers will be challenged in cohorts of 5 volunteers, with a dose ranging strategy continuing until 10 volunteers have been colonised by day 14 with a dose inducing colonisation in approximately 80% (the standard inoculum). Ten carriers will be sufficient to confirm both the genomic stability of the challenge strain and its immunogenicity.

The statistical rationale relating to the sample required for the serological response (a secondary endpoint) is that using the standard deviations of serological response to wild type *N.lactamica* derived in our first experimental human challenge study published (Evans C et al 2011) which gave SDs on a log-10 scale of 0.11 for IgA Saliva and 0.26 for Serum total IgG. Using the SD of 0.26 we will be able to confirm a 4 fold rise with 10 carriers of Nlac with 90% power using analysis of variance.

Starting at a dose of 10^5 CFU, escalating by ½-1 $\log_{10}$ and with a maximum dose of 10^7 CFU, we estimate that 15-35 volunteers will be required to identify the standard inoculum and colonise 10 volunteers with this dose.

13.3 Statistical analysis

Statistical analysis will be performed using GraphPad Prismsoftware, by the statistical unit of the NIHR Southampton Biomedical Research Centre. Serological data will be analyzed

using 1-way analysis of variance. Log-transformed data will be used to construct areas under the curve for comparison of immune responses over the study period.

14. Study quality and Management procedures

14.1 Investigator procedures

Approved site-specific SOPs will be used at all clinical and laboratory sites.

14.2 Monitoring

Monitoring will be performed by the Sponsor according to International Conference on Harmonisation (ICH) Good Clinical Practice (GCP). Following written standard operating procedures, the monitors will verify that the clinical trial is conducted and data are generated, documented and reported in compliance with the protocol, GCP and the applicable regulatory requirements. The investigator sites will provide direct access to all trial related source data/documents and reports for the purpose of monitoring and auditing by the sponsor and inspection by local and regulatory authorities.

14.3 Study amendments

No amendments to this protocol will be made without consultation with, and agreement of, the Sponsor. Any amendments to the trial that appear necessary during the course of the trial must be discussed by the investigator and sponsor concurrently. If agreement is reached concerning the need for an amendment, it will be produced in writing by the chief investigator and will be made a formal part of the protocol following ethical and regulatory approval (NRES-REC SOPs – Version 5.1 March 2012: http://www.hra.nhs.uk/wp-content/uploads/2013/08/NRES_SOPs_v5.1_2012.03.14.pdf).

An administrative change to the protocol is one that modifies administrative and logistical aspects of a protocol but does not affect the subjects' safety, the objectives of the trial and its progress. An administrative change does not require UK ethical committee or regulatory approval.

Any amendments to study documents will follow established HRA and REC requirements.

The investigator is responsible for ensuring that changes to an approved trial, during the period for which regulatory and ethical committee(s) approval has already been given, are not initiated without regulatory and ethical committee(s)' review and approval except to eliminate apparent immediate hazards to the subject.

14.4 Protocol deviation

Any deviations from the protocol will be documented in a protocol deviation form and filed in the site trial master file.

14.5 Quality Control, Quality Assurance and statutory inspection

The UHS R&D department QA staff will provide Quality Assurance (QA) for the trial and perform internal audits to check that the trial is being conducted, data recorded, analysed and accurately reported according to the protocol, Sponsor's SOPs and in compliance with ICH GCP. The audits will also include laboratory activities according to an agreed audit schedule. The internal audits will supplement the sponsor's monitoring process and will review processes not covered by the sponsor's monitor.

A Quality control (QC) plan will be established at the start of the trial, as per local SOP.

The Sponsor, trial site and ethical committee may carry out audit to ensure compliance with the protocol, GCP and appropriate regulations. GCP inspections may also be undertaken by the regulatory authority to ensure compliance with protocol and national regulations. The sponsor will assist in any inspections.

14.6 Serious breaches

A serious breach is defined as “A breach of GCP or the trial protocol which is likely to affect to a significant degree – the safety or physical or mental integrity of the subjects of the trial; or the scientific value of the trial.”

In the event that a serious breach is suspected the Sponsor will be informed as soon as possible and in turn will notify the REC and external safety committee within 7 days.

14.7 Study progress

The progress of the trial will be overseen by the Chief Investigator.

14.8 Study completion/termination

The trial will be considered complete upon the last volunteer/last visit at the site. The data will be sent to the sponsor in the timeframe specified in the Clinical Trial Agreement.

The study may be terminated early at the discretion of the Chief Investigator, Sponsor or External Safety Committee if there are safety concerns, concerns about compliance with GCP or other appropriate regulations, poor recruitment or new information becomes available which has an impact on the scientific validity or safety of the trial.

14.9 Exploitation and dissemination

The investigators will be involved in reviewing drafts of the manuscripts, abstracts, press releases and any other publications arising from the study. Findings will be published in peer reviewed journals as soon as possible, even where results prove negative. The authors will acknowledge that the study has been funded by the National Institute for Health Research, Health Protection Research Unit (NIHR HPRU), University College London. The results of the study will be disseminated at relevant international scientific meetings. Volunteers will be sent a newsletter with a lay summary of the results of the study once the results are available. This will provide information regarding the study in general and not their individual results.

15. Ethics

15.1 Declaration of Helsinki

The Investigator will ensure that this study is conducted according to the principles of the current revision of the Declaration of Helsinki 2008.

15.2 ICH guidelines for good clinical practice

The Investigator will ensure that this study is conducted in full conformity to the ICH guidelines for GCP (CPMP/ICH/135/95) July 1996.

15.3 Informed consent

Written informed consent will be gained from all participants following the provision of detailed information about the aims of the study, the level of involvement required, and the risks involved. Participants will be provided with an information sheet prior to the start of the study either in print form or via email. They will be encouraged to use the contact details on this form to contact the research team to get further information if necessary. Prior to screening the participants' understanding of the study and risks involved will be explored and they will be asked to sign a consent form.

15.4 Informing participants' General Practitioners

A letter describing the study and the participant's involvement will be sent to their General Practitioner (GP) on the day of the screening visit. This will include contact details for the research team.

15.5 Research ethics committee

A copy of the protocol, proposed informed consent form, other written volunteer information and the proposed advertising material will be submitted to the REC and HRA for written approval, using the UK Integrated Research Application System. The investigator will submit and, where necessary, obtain approval from the REC for all subsequent amendments to the protocol and associated trial documents. A non-substantial amendment does not require UK ethical committee approval (NRES-REC SOPs – Version 5.1 March 2012: http://www.hra.nhs.uk/wp-content/uploads/2013/08/NRES_SOPs_v5.1_2012.03.14.pdf). The investigator will notify deviations from the protocol or SAEs occurring at the site to the sponsor and will notify the REC of these if necessary in accordance with procedures.

15.6 Volunteer confidentiality

All data will be link anonymised; volunteer data will be identified by a unique participant number in the CRF and database. Separate confidential files containing identifiable information will be stored in secured locations. Only the sponsor representative, investigators, the clinical monitor, the ethical committee(s) and the regulatory authorities will have access to the records.

16. Data handling and record keeping

16.1 Data handling

The chief investigator will be the data manager with responsibility for delegating the receiving, entering, cleaning, querying, analysing and storing of all data that accrues from the study in the site file held in the NIHR-CRF. The investigators will enter the data into the volunteers' CRFs, which will be in a paper format. This includes safety data, laboratory data (both clinical and immunological) and outcome data. Data will then be transcribed into a secure electronic database.

16.2 Record keeping

The investigators will maintain and retain appropriate medical and research records and essential documents for this trial in compliance with ICH E6 GCP and regulatory and institutional requirements for the protection of confidentiality of volunteers. The chief investigator, co-investigators and clinical research nurses will have access to records. The investigators will permit authorised representatives of the sponsor, regulatory agencies and the monitors to examine (and when required by applicable law, to copy) clinical records for the purposes of quality assurance reviews, audits and evaluation of the study safety and progress.

16.3 Source data and case report forms (CRFs)

All protocol-required information will be collected in CRFs designed by the investigator. All source documents, excluding hospital records, will be filed in the CRF. Source documents are original documents, data, and records from which the volunteer's CRF data are obtained. For this study these will include, but are not limited to; volunteer consent form, blood results, GP response letters, laboratory records and correspondence. In the majority of cases, CRF entries will be considered source data as the CRF is the site of the original recording (i.e. there is no other written or electronic record of data). In this study this will include, but is not limited to medical history, medication records, vital signs, physical examination records, urine assessments, blood results, adverse event data and details of study interventions. All source data and volunteer CRFs will be stored securely.

16.4 Data protection

The study protocol, documentation, data and all other information generated will be held in strict confidence. No information concerning the study or the data will be released to any unauthorised third party, without prior written approval of the sponsor. All study documents and clinical samples will be labelled with a unique participant ID. This participant ID will be linked to identifiable personal information on the informed consent form, GP letter and in hospital medical notes all of which will be stored securely. All other study documents and clinical samples will be link anonymised. No personal identifiable information will be stored in the electronic database.

17. Financing and insurance

17.1 Financing

The study will be will be supported by funding from the National Institute for Health Research, Health Protection Research Unit (NIHR HPRU) in Mucosal Immunology which is based at University College London with support from the Southampton NIHR CRF, the Wessex Comprehensive Research Network.

17.2 Insurance

The University of Southampton has a specialist insurance policy in place, which would operate in the event of any participant suffering harm as a result of their involvement in the research.

17.3 Compensation for time

Volunteers will be compensated for their time and for the inconvenience caused by procedures as below.

- Attending screening and follow-up sessions - £40 total (£30/visit plus additional £10 travel expenses)
- 1 x screening
- 1x challenge
- Up to 6 follow up visits

Optional 2 year sub-study - £60 plus up to £15 travel expenses.

The maximum individual volunteers will be compensated is £400 and the minimum £20

If volunteers withdraw from the study prior to its completion they will be offered financial re-imbursement corresponding to the number of visits attended.

18. References

Bidmos, F. A., K. R. Neal, N. J. Oldfield, D. P. Turner, D. A. Ala'Aldeen and C. D. Bayliss (2011). "Persistence, replacement, and rapid clonal expansion of meningococcal carriage isolates in a 2008 university student cohort." J Clin Microbiol **49**(2): 506-512.

Brown, N. M., N. K. Ragge and D. C. Speller (1987). "Septicaemia due to Neisseria lactamica--initial confusion with Neisseria meningitidis." J Infect **15**(3): 243-245.

Cartwright, K. A., J. M. Stuart, D. M. Jones and N. D. Noah (1987). "The Stonehouse survey: nasopharyngeal carriage of meningococci and Neisseria lactamica." Epidemiol Infect **99**(3): 591-601.

Davenport, V., T. Guthrie, J. Findlow, R. Borrow, N. A. Williams and R. S. Heyderman (2003). "Evidence for naturally acquired T cell-mediated mucosal immunity to Neisseria meningitidis." J Immunol **171**(8): 4263-4270.

Deasy, A. M., E. Guccione, A. P. Dale, N. Andrews, C. M. Evans, J. S. Bennett, H. B. Bratcher, M. C. Maiden, A. R. Gorringe and R. C. Read (2015). "Nasal Inoculation of the Commensal Neisseria lactamica Inhibits Carriage of Neisseria meningitidis by Young Adults: A Controlled Human Infection Study." Clin Infect Dis **60**(10): 1512-1520.

Denning, D. W. and S. S. Gill (1991). "Neisseria lactamica meningitis following skull trauma." Rev Infect Dis **13**(2): 216-218.

Evans, C. M., C. B. Pratt, M. Matheson, T. E. Vaughan, J. Findlow, R. Borrow, A. R. Gorringe and R. C. Read (2011). "Nasopharyngeal colonization by Neisseria lactamica and induction of protective immunity against Neisseria meningitidis." Clin Infect Dis **52**(1): 70-77.

Fraser, A., A. Gafter-Gvili, M. Paul and L. Leibovici (2006). "Antibiotics for preventing meningococcal infections." Cochrane Database Syst Rev(4): CD004785.

Glennie, S. J., D. Banda, W. Mulwafu, R. Nkhata, N. A. Williams and R. S. Heyderman (2012). "Regulation of naturally acquired mucosal immunity to Streptococcus pneumoniae in healthy Malawian adults and children." PLoS One **7**(12): e51425.

Lauer, B. A. and C. E. Fisher (1976). "Neisseria lactamica meningitis." Am J Dis Child **130**(2): 198-199.

Laver, J. R., S. E. Hughes and R. C. Read (2015). "Neisserial Molecular Adaptations to the Nasopharyngeal Niche." Adv Microb Physiol **66**: 323-355.

Pugsley, M. P., D. L. Dworzack, E. A. Horowitz, T. A. Cuevas, W. E. Sanders, Jr. and C. C. Sanders (1987). "Efficacy of ciprofloxacin in the treatment of nasopharyngeal carriers of Neisseria meningitidis." J Infect Dis **156**(1): 211-213.

Read, R. C., D. Baxter, D. R. Chadwick, S. N. Faust, A. Finn, S. B. Gordon, P. T. Heath, D. J. Lewis, A. J. Pollard, D. P. Turner, R. Bazaz, A. Ganguli, T. Havelock, K. R. Neal, I. O. Okike, B. Morales-Aza, K. Patel, M. D. Snape, J. Williams, S. Gilchrist, S. J. Gray, M. C. Maiden, D. Toneatto, H. Wang, M. McCarthy, P. M. Dull and R. Borrow (2014). "Effect of a quadrivalent meningococcal ACWY glycoconjugate or a serogroup B meningococcal vaccine on meningococcal carriage: an observer-blind, phase 3 randomised clinical trial." Lancet **384**(9960): 2123-2131.

Trotter, C. L., N. J. Gay and W. J. Edmunds (2006). "The natural history of meningococcal carriage and disease." Epidemiol Infect **134**(3): 556-566.

Vaughan, A. T., A. Gorringe, V. Davenport, N. A. Williams and R. S. Heyderman (2009). "Absence of mucosal immunity in the human upper respiratory tract to the commensal bacteria Neisseria lactamica but not pathogenic Neisseria meningitidis during the peak age of nasopharyngeal carriage." J Immunol **182**(4): 2231-2240.

Wright, A. K., M. Bangert, J. F. Gritzfeld, D. M. Ferreira, K. C. Jambo, A. D. Wright, A. M. Collins and S. B. Gordon (2013). "Experimental human pneumococcal carriage augments IL-17A-dependent T-cell defence of the lung." PLoS Pathog **9**(3): e1003274.
