# Supplementary material for: Controlled Human Infection of Healthy Adults With Lyophilized Neisseria lactamica Induces Asymptomatic, Immunogenic Nasopharyngeal Carriage in the United Kingdom and Mali
Source: Open Forum Infect Dis. 2026 Jan 7;13(1):ofaf809. doi: 10.1093/ofid/ofaf809 (PMC12822493; doi:10.1093/ofid/ofaf809)
Supplement: ofaf809_Supplementary_Data [file ofaf809_supplementary_data.zip › LacE1 (Mali study) Protocol v5.0 Clean_English.pdf]

A human controlled infection study to assess colonisation and immunogenicity following nasal inoculation of Malian adults with reconstituted lyophilised wild type *Neisseria lactamica* : Lactamica Etape 1

Clinical trial protocol

Funded by:
National Institute for Health Research
Mucosal Pathogens Research Unit
University College London

Sponsor:
CVD Mali
Centre pour le Développement des Vaccins du Mali
Bamako, Mali

Challenge material:
Neisseria lactamica strain Y92-1009

Manufacturing site:
University of Southampton
C Level
South Lab & Pathology Block
University Hospital Southampton NHS Foundation Trust
Tremona Road, Southampton, SO16 6YD

Formulation:
Lyophilised powder reconstituted in sterile water / 0.9% saline

Route of administration:
Intranasal

Chief Investigator:
Professor Robert C. Read
MD FRCP
University of Southampton
Southampton
University Hospital Southampton NHS Foundation Trust
Tremona Road, Southampton, SO16 6YD
Email: r.c.read@soton.ac.uk

Local Principal Investigator:
Professor Samba Sow, MD
Centre pour le Développement des Vaccins du Mali
Bamako, Mali

Co-Principal Investigator:
Professor Milagritos Tapia, MD
University of Maryland School of Medicine
Center for Vaccine Development (CVD)
Baltimore, MD, USA

Investigators:

**Fadima Haidara**<br>MD,Sub-Inv.<br><br>**Fatoumata Diallo**<br>MD, Sub-Inv.<br><br>**Youssouf Traore**<br>MD, Study coordinator<br><br>**Henry Badji, Lab microbiology**<br><br>**Adama Mamby Keita**<br>MD, Sub-Inv.<br><br>**Flanon Coulibaly**, MD, Sub-Inv.<br><br>**Awa Traoré**, PharmD, Lab immunology<br><br>**Professor Tieman Diarra**<br><br>**Uma Onwuchekwa,** Department of biostatistic	Centre pour le Développement des Vaccins du Mali<br>Bamako<br>Mali
**Diane Gbesemete**<br>BM, MRCPCH,<br>Clinical Research Fellow<br><br>**Muktar Ibrahim**<br>Msc<br>Research Fellow<br><br>**Jay Laver**<br>PhD<br>Senior Research Fellow	University of Southampton<br>University Hospital Southampton NHS Foundation Trust<br>Tremona Road<br>Southampton<br>SO16 6YD
**Jennifer MacLennan**<br>PhD<br>Senior Clinical Researcher	Department of Zoology<br>University of Oxford
**Professor Robert Heyderman**<br>PhD FRCP	NIHR Global Health Research Unit on Mucosal Pathogens<br>Division of Infection and Immunity<br>University College London
**Professor Andrew Gorringe**<br>PhD	Public Health England<br>Porton Down,Salisbury

**Trial Site:**	**CVD Mali**<br>Centre pour le Développement des Vaccins du Mali<br>Bamako<br>Mali
**Study safety committee**	**Professor Robert Heyderman PhD FRCP (Chair)**<br>NIHR Global Health Research Unit on Mucosal Pathogens<br>Division of Infection and Immunity<br>University College London<br><br>**Professor Samba Sow, MD**<br>Centre pour le Développement des Vaccins du Mali<br>Bamako, Mali<br><br>**Diane Gbesemete, BM MRCPCH**<br>Clinical Research Fellow<br>NIHR Clinical Research Facility<br>University of Southampton<br>University Hospital Southampton NHS Foundation Trust<br><br>**Jennifer MacLennan PhD**<br>Senior Clinical Researcher<br>Department of Zoology<br>University of Oxford<br><br>**Professor Milagritos Tapia**, **MD**<br>University of Maryland School of Medicine<br>Center for Vaccine Development (CVD)<br>Baltimore<br><br>**Professor Robert C. Read MD FRCP**<br>University of Southampton<br>University Hospital Southampton NHS Foundation Trust<br>Tremona Road, Southampton, SO16 6YD
**Study monitor:**	**Clinipharm**
**Protocol version number:**	5.0
**Protocol version date:**	20 May 2021

Table of Contents

1 Sigles and abbreviations 8
2 Synopsis 9
3 Background and rationale 13
3.1 The rationale for the current study 13
3.2 *Neisseria lactamica* and *Neisseria meningitidis* 13
3.3 Carriage of *Neisseria* spp 14
3.4 Vaccine induced immunity to carriage 14
3.5 Meningococcal disease in the meningitis belt 14
3.6 Controlled human infection with *Neisseria lactamica* 15
3.7 Lyophilised *N. lactamica* 15
4 Objectives 16
4.1 Primary objective 16
4.2 Secondary objectives 16
4.3 Exploratory objectives 16
5 Endpoints 16
5.1 Safety endpoints 16
5.2 Acceptability endpoints 16
5.3 Microbiological endpoints 17
5.4 Immunological endpoints 17
6 Description and justification of the study design 17
6.1 Overview 17
6.2 Starting dose 17
6.3 Dose escalation 17
6.4 Challenge procedure 19
6.5 Assessment of colonisation 19
6.6 Definition of the start and the end of the study 19
7 Study setting and recruitment 19
7.1 Study site 19
7.2 Study population 20
7.3 Duration of volunteer participation 20
7.4 Recruitment 20
7.5 Consent 20
7.6 Eligibility criteria 20
7.6.1 Inclusion criteria 20

7.6.2 Exclusion criteria........ 21
7.6.3 Effective contraception for female volunteers........ 21
7.7 Potential benefits for volunteers........ 22
7.8 Mitigation of infection risk during the COVID-19 pandemic........ 22
8 Inoculum........ 22
8.1 Selection of strain of *N. lactamica*........ 22
8.2 Supply and storage of the stock strain........ 22
8.3 Lyophilisation of *N. lactamica*........ 23
8.4 Transport of the lyophilised stock to Mali........ 23
8.5 Storage and quality control of the lyophilised stock in Mali........ 23
8.6 Preparation of nasal inoculum........ 23
8.7 Monitoring of the *N. lactamica* dose administered to volunteers........ 23
9 Conduct of the study........ 23
9.1 Study documentation........ 23
9.2 Study schedule........ 24
9.3 Visit 1 – Screening........ 24
9.3.1 Consent........ 24
9.3.2 Screening........ 24
9.3.3 Baseline investigations........ 25
9.4 Visit 2 – Challenge........ 25
9.4.1 Review of eligibility........ 25
9.4.2 Baseline investigations........ 25
9.4.3 Inoculation........ 25
9.5 Visit 3........ 25
9.6 Visit 4........ 26
9.7 Visit 5........ 26
9.8 Visit 6........ 26
9.9 Visit 7........ 26
9.10 Withdrawal of volunteers........ 26
9.11 Compensation........ 27
10 Clinical and laboratory monitoring........ 27
10.1 Monitoring of volunteers........ 27
10.2 Management of clinical symptoms........ 27
10.2.1 Phlebotomy........ 28
10.2.2 Inoculation with N. lactamica........ 28
10.2.3 Throat swab samples........ 28

11 Laboratory procedures 28
11.1 Laboratory work 28
11.2 Labelling of samples 28
11.3 Processing of clinical samples 28
12 Assessment of safety 29
12.1 Safety Evaluation 29
12.2 Adverse Event (AE) 29
12.3 Adverse Reaction (AR) 29
12.4 Unexpected Adverse Reaction (UAR) 30
12.5 Serious Adverse Event (SAE) 30
12.6 Serious Adverse Reaction (SAR) 30
12.7 Suspected Unexpected Serious Adverse Reactions (SUSARs) 30
12.8 Procedures to be followed in the event of abnormal findings 30
12.9 Foreseeable medical occurrences 30
12.10 Severity grading of clinical adverse events 31
12.11 Causality assessment 33
12.12 Reporting procedures for AEs 33
13 Acceptability and feasibility assessment 34
13.1 Overview 34
13.2 Research questions 34
13.3 Geographical location 35
13.4 Discussant characteristics 35
13.5 Consent methods 35
13.6 Assessment methods 35
13.7 Assessment instruments 35
14 Statistical analysis 35
14.1 Sample size 35
14.2 Statistical analysis 36
15 Quality control and quality assurance 36
15.1 Investigator procedures 36
15.2 Quality Control 36
15.3 Quality Assurance 36
15.3.1 Internal Monitoring 36
15.3.2 External Monitoring 36
16 Regulatory and Ethical Requirements 36
16.1 Institutional Review Boards and Ethics Committees 37

16.2 Local Community Permission ... 37
16.3 Study amendments ... 37
16.4 Protocol deviation ... 37
16.5 Serious breaches ... 37
16.6 Study completion/termination ... 37
16.7 Exploitation and dissemination ... 38
17 Data handling and record keeping ... 38
17.1 Data handling ... 38
17.2 Volunteer confidentiality ... 38
18 Financing ... 38
19 References ... 39

1 Sigles and abbreviations

AE	Adverse Event
AR	Adverse Reaction
CFU	Colony forming units
CNS	Central nervous system
CVD-Mali	Centre pour le Développement des vaccins du Mali
COVID-19	Coronavirus Desease 2019
LyoNlac	Lyophilised *Neisseria lactamica*
MPRU	Mucosal Pathogens Research Unit
Nlac	*Neisseria lactamica*
Nmen	*Neisseria meningitidis*
PCR	Polymerase Chain Reaction
PBS	Phosphate Buffered Saline
SAE	Serious adverse event
SOP	Standard Operating Procedure
SUSARs	Suspected Unexpected Serious Adverse Reaction
UCL	University College London
UAR	Unexpected Adverse Reaction

2 Synopsis

**Title**	**A human controlled infection study to assess colonisation and immunogenicity following nasal inoculation of Malian adults with reconstituted lyophilised wild type *Neisseria lactamica*: Lactamica Etape 1**
**Sponsor**	Centre pour le Développement des Vaccins du Mali<br>Bamako, Mali
**Trial Centre**	Centre pour le Développement des Vaccins du Mali<br>Bamako, Mali
**Design**	Prospective dose ranging human challenge study<br>Nasal inoculation with reconstituted, previously lyophilised *Neisseria lactamica* with dose escalation
**Population**	Healthy Malian volunteers aged 18-45 years
**Sample size**	Total up to 100 volunteers<br>Dose ranging study – up to 5 volunteers per dose cohort
**Follow up duration**	Challenge at day 0, follow up on Days 4, 7, 14, 28 and 168
**Planned Trial Period**	November 2020-November 2022
**Primary Objective**	i. To demonstrate successful nasopharyngeal colonisation of healthy Malian adults with nasally administered reconstituted lyophilised *Neisseria lactamica*
**Secondary Objectives**	i. To assess the safety of nasal inoculation of healthy Malian adults with reconstituted lyophilised *Neisseria lactamica*<br>ii. To establish the dose of nasally administered reconstituted lyophilised *Neisseria lactamica* required to induce nasopharyngeal colonisation in at least 70% of healthy Malian adults<br>iii. To demonstrate the immunogenicity of colonisation induced by nasal inoculation with reconstituted lyophilised *Neisseria lactamica* in healthy Malian adults<br>iv. To assess the acceptability of nasal inoculation of healthy Malian adults with reconstituted lyophilised *Neisseria lactamica*

**Exploratory Objectives**	i.	To assess the duration of colonisation induced by nasal inoculation with reconstituted lyophilised *Neisseria lactamica* in healthy Malian adults
**Safety endpoints**	i.<br>ii.<br>iii.	Occurrence of solicited reactogenicity within 25 minutes post-inoculation<br>Occurrence of unsolicited adverse events within 28 days post-inoculation<br>Occurrence of serious adverse events within the study period
**Acceptability endpoints**	i.	Evidence of saturation for concept elicitation
**Microbiological endpoints**	i.<br>ii.	Assessment of successful induction of colonisation with *Neisseria lactamica* – culture of *Neisseria lactamica* from throat swabs taken between Day 4 and Day 7 following inoculation<br>Assessment of carriage of *Neisseria lactamica* and other *Neisseria* spp. – culture and PCR of colonies isolated from throat swabs taken at any study visit
**Immunological endpoints**	i.	Rise in serological specific antibody titre comparing Day 0 versus Day 28 post inoculation
**Challenge material**<br>**Form**<br>**Dose**<br>**Route**		Wild type *Neisseria lactamica* strain Y92-1009<br>1ml liquid - Lyophilised powder, reconstituted in sterile water / 0.9% saline<br>$10^5 – 10^7$ cfu<br>Intranasal
**Inclusion criteria**	1.<br>2.<br>3.<br>4.	Healthy adults aged 18 to 45 years inclusive on the day of enrolment residing outside the demographic surveillance area<br>Able and willing (in the investigator's opinion) to understand and comply with all study requirements including availability for all study follow up visits<br>Provide written informed consent to participate in the trial<br>For females only, all the following:<br>o willingness to practice continuous effective contraception at least until the Day 28 visit is complete

	○ negative urine pregnancy test on the screening day<br>○ negative urine pregnancy test on inoculation day
**Exclusion criteria**	1. *N. lactamica* detected on throat swab taken at the screening visit<br>2. Individuals who have an ongoing acute illness at the time of inoculation<br>3. Individuals who have been involved in other clinical trials involving receipt of an investigational product over the last 12 weeks or if there is planned use of an investigational product during the study period<br>4. Use of systemic antibiotics within the period 30 days prior to the challenge<br>5. Any confirmed or suspected immunosuppressive or immune-deficient state, including HIV infection; asplenia; recurrent, severe infections and chronic (more than 14 days) immunosuppressant medication (including oral steroids) within the past 6 months (topical steroids are allowed)<br>6. Use of immunoglobulins or blood products within 3 months prior to enrolment.<br>7. History of allergic disease or reactions to soya.<br>8. Any clinically significant abnormal finding on clinical examination or screening investigations<br>9. History of any surgery to the nose or throat in the previous 3 months<br>10. Any other significant disease, disorder, or finding which may significantly increase the risk to the volunteer because of participation in the study, affect the ability of the volunteer to participate in the study or impair interpretation of the study data<br>11. Occupational, household or intimate contact with immunosuppressed persons<br>12. Positive pregnancy test or lactation

Study schedule	Visit 1 Screening	Visit 2 Challenge	Visit 3	Visit 4	Visit 5	Visit 6	Visit 7
Timeline (days)	-2 to -17	0	4	7	14	28	168
Visit window				+/-1	+/- 2	+/- 3	+/-42
Volunteer Information Sheet	+						
Informed consent	+						
Vital signs	+	+	+	+	(+)	(+)	(+)
Medical history	+						
Physical examination	+	(+)	+	+	(+)	(+)	(+)
Urine pregnancy test (females only)	+	+					
Review eligibility	+	+					
Inoculation		+					
Photograph		+					
Review of adverse events and concomitant medications			+	+	+	+	
Review of serious adverse events and concomitant medications							+
Throat swab (microbiology)	+	+	+	+	+	+	+
Safety bloods (ml)	10		10				
Immunological blood tests (ml)		20				20	
Cumulative blood volume (ml)	10	30	40			60	

3 Background and rationale

3.1 The rationale for the current study

This study is funded by the Mucosal Pathogens Research Unit (MPRU) based at University College London (UCL). It is part of a programme of research investigating controlled human infection with the nasopharyngeal commensal *Neisseria lactamica* (Nlac) and its interaction with *Neisseria meningitidis* (Nmen), the causative agent of meningococcal disease. Previous studies in the United Kingdom have shown that nasal inoculation of healthy adult volunteers with Nlac can induce safe, long-standing colonisation, which is immunogenic, inducing specific mucosal and systemic immune responses and some cross-protective responses against Nmen. An Nlac-induced suppression of meningococcal carriage has also been demonstrated. The aim of this study is to validate the use of this controlled human infection model in Malian adults in order to investigate the ability to induce immunising colonisation with Nlac within the meningitis belt.

We have previously nasally inoculated over 400 volunteers with Nlac derived from frozen stocks. The use of frozen stocks of Nlac is relatively unsatisfactory because of the instability of frozen stocks and rapid loss of viability after thawing and would not be practical in Mali. A recent UK based study has shown successful colonisation of volunteers with a modification of the methodology for inoculum preparation using reconstituted lyophilised Nlac (lyoNlac). This facility to reconstitute dry powdered lyoNlac into water or saline and to inoculate as a nose drop greatly simplifies the experimental method.

In summary this is a pilot study of the safety, efficacy and immunogenicity of the intranasal inoculation of healthy Malian volunteers with lyoNlac. We aim to determine the dose of inoculum required to induce colonisation in at least 70% of volunteers (the Standard Inoculum or SI). This SI will be used in future studies aiming to optimise duration of colonisation and immunogenicity induced by lyoNlac.

3.2 *Neisseria lactamica* and *Neisseria meningitidis*

Nlac and Nmen are Gram negative diplococci which both colonise the human nasopharynx. Nlac is non-pathogenic, non-encapsulated and lactose-fermenting and is a common commensal, particularly in young children. Transmission occurs through close contact and only a few cases of clinical significance have been reported (1-3).

Nmen is a human-adapted, often encapsulated species that uses the human nasopharynx as its sole biological niche (4). In the vast majority of interactions with humans, the organism colonises silently, and invasion is extremely rare. When invasion occurs, Nmen can cause fulminant bloodstream or CNS infection with a high case fatality rate and devastating sequelae for survivors. Outbreaks and epidemics can occur, particularly in the meningitis belt of sub-Saharan Africa where meningococcal disease remains a significant public health challenge.

There is an inverse epidemiological relationship between carriage of the commensal Nlac and meningococcal carriage and disease (5). The mechanism of this relationship is undetermined, but is not due to cross-protective antibody production, as the early years of life associated with high rates of Nlac carriage predate the development of natural bactericidal meningococcal antibodies (6).

3.3 Carriage of *Neisseria* spp.

The highest rate of natural carriage of Nlac occurs in infants. Carriage wanes in toddlers and older children and by the time a person reaches adolescence, carriage is about 1%. By contrast, carriage of Nmen is lowest in infants, increasing gradually as childhood progresses. The highest rates of carriage are seen in teenagers and University students. Although carriage of the organism is quite common, disease is extremely rare – currently less than 1 per 100,000 per annum in the UK. Therefore, the likelihood of disease even when a person is carrying a virulent strain of Nmen, is very low (6).

Although Nlac and Nmen colonise the same location within the upper respiratory tract, previous studies suggest they engage with the human mucosal immune system in very different ways (7). In contrast to Nmen, Nlac maintains a purely commensal relationship with the host. Nlac lacks a polysaccharide capsule, so any adaptive immune responses to this bacterium must be directed at non-capsular antigens, providing a good platform for assessing non-anti-polysaccharide immunity against colonising bacteria when compared with wild type Nlac.

3.4 Vaccine induced immunity to carriage

Asymptomatic oropharyngeal carriage of Nmen is a prerequisite for meningococcal disease. National programmes administering glycoconjugate vaccines have had dramatic effects on disease incidence, at least partly due to herd protection conferred by reduced carriage and transmission (8). The subcapsular vaccine, 4CMenB induces bactericidal antibodies against a range of strains, including serogroup B, but a large prospective randomised study has shown that the effect on carriage of serogroup B *N. meningitidis* is relatively modest (9).

Future successful vaccines will target pathogen colonisation, using antigens known to induce immunity critical for colonisation, in age groups most likely to transmit to others.

3.5 Meningococcal disease in the meningitis belt

The meningitis belt of Sub-saharan Africa has a high incidence of meningococcal disease with both endemic disease and cyclical epidemics. The increase in meningococcal meningitis incidence occurs annually with peaks coinciding with the middle of the dry season and abating with the onset of the rainy season. Weekly incidence rates increase by a factor of 10-100 in comparison to the baseline rate outside of this season (10, 11).

Sporadic localised epidemics or outbreaks also occur intermittently and more widespread epidemic waves, affecting larger regions over more than one season, occur at irregular periods of approximately every 5-12 years (10, 12).

The main pathogen responsible for outbreaks and epidemics in the meningitis belt has historically been *N. meningitidis* serogroup A. With the introduction of a serogroup A vaccine MenAfriVac, the disease incidence due to serogroup A has declined dramatically, but other serogroups of *N. meningitidis* such as W, C and X have been identified in localised outbreaks and occasional larger epidemics (10, 13).

Control strategies include the use of routine vaccination with MenAfriVac as well as active surveillance and reactive serogroup specific vaccination programmes when an outbreak is detected (14). The implementation of these strategies involves significant challenges and may not always be achievable in the required time frame (15, 16). One limitation to the

timely accessibility of vaccines is the requirement for a cold chain, which may be logistically challenging in a resource poor setting (14).

Future epidemic control strategies must focus on the speed of implementation and carriage reduction, as well as considering the effects on anti-microbial resistance, the required infrastructure and cost. An ideal form of reactive prophylaxis would be cheap, readily available with no requirement for a cold chain, easy to administer and have no impact on anti-microbial resistance.

3.6 Controlled human infection with *Neisseria lactamica*

Nlac has been shown to be safe in controlled human infection as we have found in over 350 volunteers experimentally nasally inoculated with the wild type organism (17, 18). Even at a very low dose of 10^4 colony forming units (CFU), long lasting colonisation with Nlac is easily induced in 35-65% of participants. In 80-90% of those successfully colonised, this is detectable by 1-2 weeks after inoculation. (17, 18). In a subgroup of volunteers who were not successfully colonised despite two challenges with 10^4 CFU, increasing the inoculum dose to 10^5 CFU resulted in successful colonisation of 50% of the subgroup (17). Colonisation has a clear effect on the nasal mucosal microbiome, in that meningococcal acquisition is effectively inhibited in participants who carry the organism (18). Colonisation is immunogenic with an increase in specific serum IgG by 2 weeks and specific salivary IgA by 4 weeks (17).

Colonisation by Nlac is an immunising event; we proved this in humans by inoculating university students intranasally with Nlac and we observed both specific systemic and mucosal antibody responses by 4 weeks. However, this did not induce significant cross-reactive serum bactericidal antibodies to Nmen (17).

3.7 Lyophilised *N. lactamica*

In previous controlled human infection studies we have used frozen stocks of *N. lactamica* which are thawed and diluted and then administered to the participants. The disadvantages of this technique are that (a) stocks need to be maintained at -80°C, (ii) there is a gradual decline in viable counts from frozen stocks, (iii) frozen stocks are relatively difficult to transport, and (iv) dilution of frozen stock can be an inaccurate process resulting in lower or higher inocula than planned.

Lyophilisation (freeze drying) is a long-established process in which bacteria are dessicated to form a powder material which allows long term storage with efficient reconstitution to a viable state on re-addition of water or saline.

We have shown that our stock of Nlac can be lyophilised, that lyophilised Nlac (lyoNlac) can be reconstituted to yield viable CFU and that lyoNlac retains its viability for at least 3 months following the lyophilisation process. Cryoprotection of mid-to-late log phase Nlac is achieved using a mixture of commercially available soya milk (Alpro) and commercially available sucrose (10%) (w/v) in PBS, which is used to resuspend a washed bacterial cell pellet prior to freezing. Frozen bacteria are then dried in a strong vacuum overnight to generate a powder that can be stored until needed and contain defined amounts of reconstitutable bacteria.

LyoNlac is then reconstituted in sterile water or 0.9% saline to yield cultivable *N. lactamica* which is phenotypically and genetically identical to our inoculum stocks of the bacterium.

LyoNlac has been used in one controlled human infection study to date and has been shown to be safe and successfully induce colonisation. In this dose-ranging study, doses from 10^4 to 10^5 colony forming units have been nasally administered to a total of 28 volunteers to date. The colonisation fraction at a dose of 10^5 CFU was 100%. Immunological results are awaited. (Unpublished data).

4 Objectives

4.1 Primary objective

1. To demonstrate successful nasopharyngeal colonisation of healthy Malian adults with nasally administered reconstituted lyophilised *Neisseria lactamica.*

4.2 Secondary objectives

1. To assess the safety of nasal inoculation of healthy Malian adults with reconstituted lyophilised *Neisseria lactamica.*
2. To establish the dose of nasally administered reconstituted lyophilised *Neisseria lactamica* required to induce nasopharyngeal colonisation in at least 70% of healthy Malian adults.
3. To demonstrate the immunogenicity of colonisation induced by nasal inoculation with reconstituted lyophilised *Neisseria lactamica* in healthy Malian adults.
4. To assess the acceptability of nasal inoculation of healthy Malian adults with reconstituted lyophilised *Neisseria lactamica.*

4.3 Exploratory objectives

1. To assess the duration of colonisation induced by nasal inoculation with reconstituted lyophilised *Neisseria lactamica* in healthy Malian adults.

5 Endpoints

5.1 Safety endpoints

1. Occurrence of solicited reactogenicity within 25 minutes post-inoculation.
2. Occurrence of unsolicited adverse events within 28 days post-inoculation.
3. Occurrence of serious adverse events within the study period.

5.2 Acceptability endpoints

1. Evidence of saturation for concept elicitation.

5.3 Microbiological endpoints

1. Assessment of successful induction of colonisation with *Neisseria lactamica* – culture of *Neisseria lactamica* from throat swabs taken between Day 4 and Day 7 following inoculation.
2. Assessment of carriage of *Neisseria lactamica* and other *Neisseria* spp. – culture and PCR of colonies isolated from throat swabs taken at any study visit.

5.4 Immunological endpoints

1. Rise in serological specific antibody titre comparing Day 0 versus Day 28 post inoculation.

6 Description and justification of the study design

6.1 Overview

This is a dose-ranging controlled human infection study in which healthy Malian volunteers aged 18-45 years will be recruited. Participants will be inoculated intranasally with reconstituted lyoNlac (0.5 ml per nostril). This study seeks to determine the minimum dose of inoculum that results in colonisation of at least 70% of volunteers.

A dose ranging strategy will be used, challenging volunteers with reconstituted lyoNlac in cohorts of 5 volunteers per dose. The initial dose will be 10^5 colony-forming units (CFU) and will be escalated in steps of 0.5 to 1 $\log_{10}$ up to a maximum of 10^7 CFU to achieve a colonisation fraction of at least 80% (4 out of 5 volunteers). Once the minimum number of CFU sufficient to colonise 80% of 5 volunteers is determined, a further 5 volunteers will be challenged with that dose to confirm at least 70% colonisation overall (7 out of 10 volunteers). This dose will then be defined as the Standard Inoculum (SI). If the maximum dose of 10^7 CFU is reached, it will be defined as the Standard Inoculum, accepting a lower colonisation fraction. Further volunteers will be challenged with the SI until a total of 10 volunteers have been colonised at that dose as a sample size of 10 is required to show immunogenicity. Safety parameters will be monitored during each visit. Immunogenicity will be assessed at Day 28 in comparison to baseline. We estimate that the total number of volunteers required will be in the range 25-100.

6.2 Starting dose

In our previous study using reconstituted lyophilised *N. lactamica* administered intranasally, colonisation was achieved in 60% of volunteers at a dose of 10^4 CFU, 60% at a dose of $5x10^4$ CFU and 100% at a dose of 10^5 CFU with no significant safety concerns (unpublished data). 10^5 CFU has therefore been chosen as the starting dose for this study, with the dose ranging aspect allowing for the potential increase in dose required to colonise volunteers from a population with differing background carriage rates.

6.3 Dose escalation

Volunteers will be challenged in cohorts of 5 volunteers with a specified intended dose. This intended dose will then be escalated or unchanged for the following cohort of 5 depending

on the number of volunteers colonised and the actual dose administered as estimated by a viable count of the residual inoculum. The initial dose will be 10^5 CFU and the maximum dose used will be 10^7 CFU. The intended dose escalation plan is shown in Figures 6.1 and 6.2 below but smaller dose escalations or repeated doses may be agreed by the study safety committee on the basis of safety parameters or actual doses administered. This process will be repeated until the dose inducing at least 70% colonisation has been identified. Once colonisation has been induced in 7-10 out of a total of 10 volunteers at a given dose, or once the maximum dose of 10^7 CFU has been reached, this dose will be defined as the standard inoculum (SI). Further volunteers will then be challenged with the SI until a total of at least 10 volunteers have been successfully colonised with this dose.

If 70% colonisation is not achieved with 10^7 CFU then this dose may be used as the SI for future studies, accepting a lower colonisation rate.

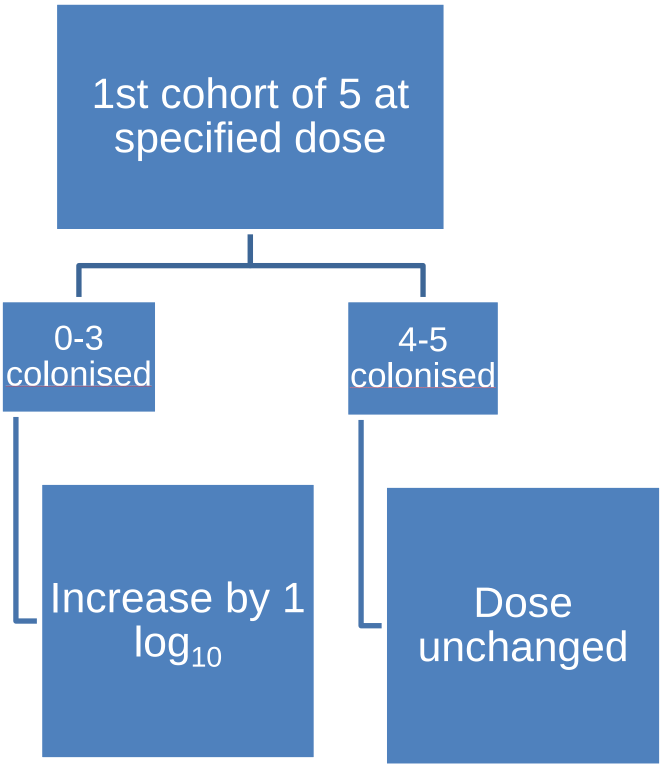

Figure 6.1: Dose ranging strategy for first cohort challenged with a specified dose. The doses for the first cohort in the study are given as an example.

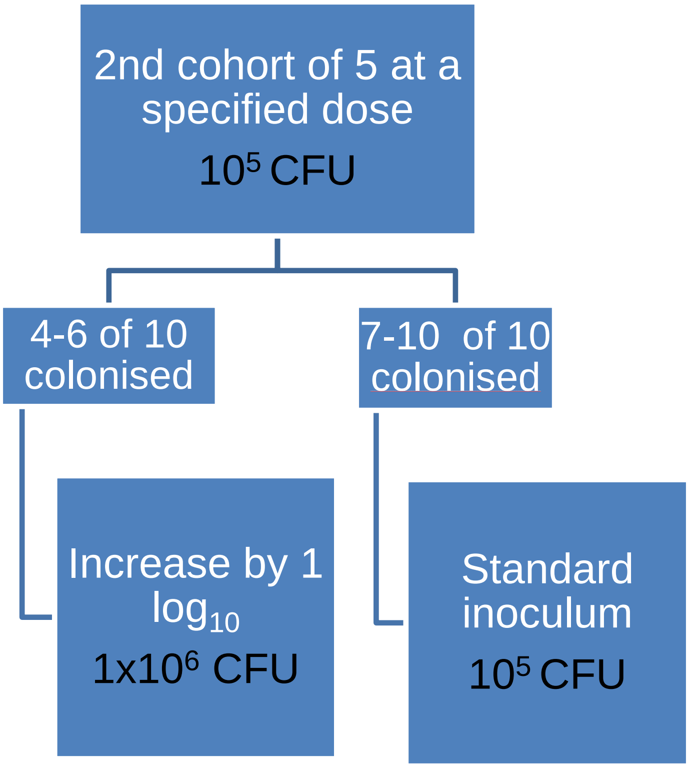

Figure 6.2: Dose ranging strategy for the second cohort challenged with a specified dose. The doses for the first cohort in the study are given as an example.

6.4 Challenge procedure

Our priority is to conduct this study without causing harm to the volunteer. As *N. lactamica* is a non-virulent commensal organism and based on previous challenge studies with this organism, we consider that the likelihood of disease resulting from inoculation of the volunteers is so low as to be considered negligible.

6.5 Assessment of colonisation

For the purposes of dose escalation, successful colonisation will be defined as the culture of viable *N. lactamica* from a throat swab taken on or before visit 4 (day 7 post inoculation).

6.6 Definition of the start and the end of the study

The start of the study is defined as the date of screening of the first volunteer. The end of the study is defined as 12 months after the date of the last visit of the last volunteer to allow for sample processing and data analysis.

7 Study setting and recruitment

7.1 Study site

The study will be conducted at Centre pour le Developpement des Vaccins- Mali (CVD-Mali). This is a research facility that is partnered with the University of Maryland School of Medicine Center for Vaccine Development in Baltimore, MD. CVD-Mali has a long history of conducting clinical trials according to international norms. The headquarters is located in Bamako, Mali. This study will be performed at the Clinical Trials Unit, a facility equipped with the necessary clinical space and pharmacy. The participants will be screened, evaluated,

inoculated and observed in the clinical space. The laboratory components of the study will be completed at the Microbiology and Molecular Biology Laboratory. All study documentation will be stored onsite.

7.2 Study population

The participants will be recruited from among the residents in quartiers other than those involved with the CVD-Mali ongoing demographic surveillance.

7.3 Duration of volunteer participation

The duration of involvement of volunteers in the study will be from visit 1 to visit 7 – a study period of approximately 175 days.

7.4 Recruitment

Participants will be recruited at the local health centers where interested persons will be asked to then come to CVD-Mali clinical trials unit. Community liaisons will be used to guide the volunteers and will be the link between the community and the study site. The liaisons will be appointed by the local health center or the community leaders.

7.5 Consent

Prior to undertaking any study-related activities, the investigator must obtain community permission to conduct the study. This process entails a series of meetings with the different community members, including the local health center personnel, local religious and cultural leaders as well as other community leaders. At each of these meetings, the investigator will explain the study procedures, the risks and benefits of study participation and other relevant aspects of the meeting. Then an audiotaped version of the consent form is played for the audience. Attendees have an opportunity to ask any questions or clarify any aspects of the study. Once all questions have been answered and if the community agrees, the attendees sign (or mark) an attendance list. These lists are stored in the study file with other study-related documentation.

After this community permission has been obtained, recruitment activities will begin in the health centers. Study personnel will meet with potential participants. The study objectives, risks and benefits and other relevant aspects will be explained. If he or she chooses, the participant may read the consent form or listen to an audiotaped version of the consent form. Since the literacy rate in Mali is ~40%, the consent form will be translated into the pertinent local languages and recorded on audiotapes. Then potential participants will be given the opportunity to ask any questions they may have. They may choose to take a copy of the form home for further review or may choose to sign the form right away. In the event that they are illiterate, they may place their mark on the signature line and an independent literate witness will sign and date the form as well.

A signed copy of the consent form will be provided to the participant for their reference. The informed consent procedure will be documented in a form that will record among other items, who provided consent and whether or not a witness was present.

7.6 Eligibility criteria

7.6.1 Inclusion criteria

The volunteer must satisfy all the following inclusion criteria to be eligible for the study:

1. Healthy adults aged 18 to 45 years inclusive on the day of enrolment residing outside the demographic surveillance area
2. Able and willing (in the investigator's opinion) to understand and comply with all study requirements including availability for all study follow up visits
3. Provide written informed consent to participate in the trial
4. For females only,
    - willingness to practice continuous effective contraception at least until the Day 28 visit is complete
    - negative pregnancy test on the screening day
    - negative pregnancy test on inoculation day

7.6.2 Exclusion criteria

The volunteer may not enter the study if any of the following criteria apply:

1. *N. lactamica* detected on throat swab taken at the screening visit
2. Individuals who have an ongoing acute illness at the time of inoculation
3. Individuals who have been involved in other clinical trials involving receipt of an investigational product over the last 12 weeks or if there is planned use of an investigational product during the study period
4. Use of systemic antibiotics within the period 30 days prior to the challenge
5. Any confirmed or suspected immunosuppressive or immune-deficient state, including HIV infection; asplenia; recurrent, severe infections and chronic (more than 14 days) immunosuppressant medication (including oral steroids) within the past 6 months (topical steroids are allowed)
6. Use of immunoglobulins or blood products within 3 months prior to enrolment.
7. History of allergic disease or reactions to soya.
8. Any clinically significant abnormal finding on clinical examination or screening investigations
9. History of any surgery to the nose or throat in the previous 3 months
10. Any other significant disease, disorder, or finding which may significantly increase the risk to the volunteer because of participation in the study, affect the ability of the volunteer to participate in the study or impair interpretation of the study data
11. Occupational, household or intimate contact with immunosuppressed persons
12. Pregnancy or lactation

7.6.3 Effective contraception for female volunteers

Female volunteers are required to use an effective form of contraception at least until the Day 28 visit is completed. Acceptable forms of contraception include:

- Established use of oral, injected or implanted hormonal methods of contraception
- Placement of an intrauterine device or intrauterine system
- Total abdominal hysterectomy
- Barrier methods of contraception (condom or occlusive cap with spermicide)
- Male sterilisation if the vasectomised partner is the sole partner for the subject
- True abstinence when this is in line with the preferred and usual lifestyle of the subject

7.7 Potential benefits for volunteers.

Volunteers will not benefit directly from participation in this study. However, it is hoped that the information gained from this study will contribute to knowledge about nasopharyngeal colonisation and therefore to the development of safe and effective vaccines in the future. Volunteers will also receive information about their general health status.

7.8 Mitigation of infection risk during the COVID-19 pandemic

Procedures and processes for the mitigation of risk of transmission between study volunteers and staff members is detailed in the SOP "**COVID-19 risk mitigation processes for Lactamica Etape 1**". These processes will include the following:

- Organisation of visits and clinical space to allow physical distancing and ensure proper air circulation
- Use of PPE – visits and clinical procedures
- Exclusion of symptomatic volunteers and staff from visits
- Screening of asymptomatic volunteers only if requested by the study safety committee
- Management and testing of symptomatic volunteers according to national policies. If testing is indicated, it would be performed as part of the national control program.

8 Inoculum

8.1 Selection of strain of *N. lactamica*

A wild-type strain of *Neisseria lactamica* strain Y92-1009 (sequence type 3493, clonal complex 613) will be used because we have previously used it safely in experimental challenge of over 350 human volunteers (18).

8.2 Supply and storage of the stock strain

Stocks of *N. lactamica* Y92-1009 (sequence type 3493, clonal complex [CC] 613) in Frantz medium containing 30% (v/v) glycerol were supplied by the Current Good Manufacturing Practices pharmaceutical manufacturing facilities at Public Health England (Porton Down, United Kingdom) as vials of $1x10^6$ bacteria, suspended in Frantz medium containing 30% (v/v) glycerol, transported and stored at -80°C. These stocks were transferred to the

University Hospital Southampton under temperature-monitored conditions and are stored at -80°C in a locked, dedicated, temperature monitored freezer.

8.3 Lyophilisation of *N. lactamica*

The lyophilised stock has been prepared in the Medical School laboratory LC70 at the University of Southampton by Dr Jay Laver, following the standard operating procedure "**Production, storage and monitoring of lyophilised *N. lactamica* inoculum for human challenge studies"** with a chain of accountability recorded at a GMP-like standard.

8.4 Transport of the lyophilised stock to Mali

Vials of lyophilised Nlac will be transported to CVD Mali by courier under refrigerated conditions.

8.5 Storage and quality control of the lyophilised stock in Mali

Vials of lyophilised Nlac will be stored in a refrigerator (at 2-8°C) in a laboratory with restricted access in CVD Mali. The stock vials will be monitored for purity and viable count on arrival in Mali and following each inoculation, according to the SOP: **Preparation and monitoring of lyoNlac for nasal inoculation (Lac Etape 1)**

8.6 Preparation of nasal inoculum

The nasal inoculum will be prepared for inoculation by reconstituting lyoNlac in sterile water or 0.9% saline following the SOP: **Preparation and monitoring of lyoNlac for nasal inoculation (Lac Etape 1)**

8.7 Monitoring of the *N. lactamica* dose administered to volunteers

After the inoculum is given, a sample of the residual inoculum will be diluted and cultured overnight according to the SOP: **Preparation and monitoring of lyoNlac for nasal inoculation (Lac Etape 1)**. The actual dose administered will then be estimated from the viable count and the species confirmed.

9 Conduct of the study

9.1 Study documentation

All study-related data will be collected on paper case report forms and/or an electronic database. The database will be stored on a server at CVD-Mali and will be password-protected.

9.2 Study schedule

	Visit 1 Screening	Visit 2 Challenge	Visit 3	Visit 4	Visit 5	Visit 6	Visit 7
Timeline (days)	-2 to -17	0	4	7	14	28	168
Visit window				+/-1	+/- 2	+/- 3	+/-42
Volunteer Information Sheet	+						
Informed consent	+						
Vital signs	+	+	+	(+)	(+)	(+)	(+)
Medical history	+						
Physical examination	+	(+)	+	(+)	(+)	(+)	(+)
Urine pregnancy test (females only)	+	+					
Review eligibility	+	+					
Inoculation		+					
Photograph		+					
Review of adverse events and concomitant medications			+	+	+	+	
Review of serious adverse events and concomitant medications							+
Throat swab (microbiology)	+	+	+	+	+	+	+
Safety bloods (ml)	10		10				
Immunological blood tests (ml)		20				20	
Cumulative blood volume (ml)	10	30	40			60	

9.3 Visit 1 – Screening

This visit will take place 2 to 17 days prior to inoculation.

9.3.1 Consent

The volunteer will be fully informed of all aspects of the trial, the potential risks and their obligations and will give informed consent as described in section 6.5 before any study specific procedures are performed.

9.3.2 Screening

A set of screening questions, a medical history and a physical examination including vital signs will be completed to ensure that the volunteer meets all inclusion and no exclusion criteria. Females will have a urine pregnancy test as part of the screening process.

9.3.3 Baseline investigations

Eligible volunteers will have a throat swab to look for carriage of *N. lactamica,* and other *Neisseria* species including *N. meningitidis* at baseline. Blood tests will be taken as a baseline for safety analysis. Results that fall outside of the normal range may not be of clinical significance but should be considered on an individual basis and may be repeated once prior to confirming eligibility.

9.4 Visit 2 – Challenge

9.4.1 Review of eligibility

The volunteer will be given an opportunity to ask further questions and asked to confirm that they are still willing to continue with the study. An interim medical history and physical examination will be performed and female volunteers will have a repeat pregnancy test to ensure that they remain eligible for the study.

9.4.2 Baseline investigations

Eligible volunteers will have a throat swab to look for carriage of *N. lactamica* and other *Neisseria* species prior to inoculation. Blood tests will be taken as a baseline for immunological analysis.

9.4.3 Inoculation

9.4.3.1 Location of the challenge

The challenge will be performed at the CVD-Mali Clinical Trials Unit.

9.4.3.2 Preparing the inoculum

The inoculum will be prepared in the CVD-Mali laboratory using a dedicated category II safety hood by technical staff. Two people will be present during preparation: one team member will prepare the inoculum, while the other team member will check the procedure which will be carried out according to the SOP: **Preparation and monitoring of lyoNlac for nasal inoculation**.

9.4.3.3 Administering the inoculum

The challenge procedure will be carried out by according to the SOP: **Performance of nasal inoculation for Lactamica Etape 1**. The volunteer will remain supine for 5 minutes following inoculation.

9.4.3.4 Post inoculation observation

The participant will move to an observation area and will be observed for a further 20 minutes. During this time, they will have a photograph taken for their study identification card. Coughing, sneezing, nasal irritation and any other adverse events will be recorded.

9.5 Visit 3

This visit will take place 4 days following inoculation. An interim history and physical examination including vital signs will be completed to assess for AEs and to document the general health of the participant. A set of safety bloods will be obtained and throat swab will be taken to assess colonisation with *N. lactamica*.

9.6 Visit 4

This visit will take place 7 days (+/- 1 day) following inoculation. An interim history and physical examination including vital signs will be completed to assess for AEs and to document the general health of the participant. A throat swab will be taken to assess colonisation with *N. lactamica*.

9.7 Visit 5

This visit will take place 14 days (+/- 2 days) following inoculation. An interim history will be taken to assess for AEs and to document the general health of the participant. If there are any safety concerns then a physical examination with vital signs will be completed. A throat swab will be taken to assess colonisation with *N. lactamica*.

9.8 Visit 6

This visit will take place 28 days (+/- 3 days) following inoculation. An interim history will be taken to assess for AEs and to document the general health of the participant. If there are any safety concerns then a physical examination with vital signs will be completed. A throat swab will be taken to assess colonisation with *N. lactamica*. Blood tests will be taken for immunological analysis.

9.9 Visit 7

This visit will take place 168 days (+/- 42 days) following inoculation. An interim history will be taken to assess for SAEs and to document the general health of the participant. If there are any safety concerns then a physical examination with vital signs will be completed. A throat swab will be taken to assess colonisation with *N. lactamica*. This visit will conclude the volunteer's participation in the study

9.10 Withdrawal of volunteers

In accordance with the principles of the current revision of the Declaration of Helsinki (updated 2008) and any other applicable regulations, a volunteer has the right to withdraw from the study at any time and for any reason and is not obliged to give his reasons for doing so. In addition, the volunteer may withdraw/be withdrawn from further study procedures at any time in the interests of the volunteer's health and well-being, or for any of the following reasons:

- Administrative decision by the Investigator.
- Ineligibility (either arising during the study or retrospectively, having been overlooked at screening).
- Significant protocol deviation.
- Volunteer non-compliance with study requirements.
- An AE, which requires discontinuation of the study involvement or results in inability to continue to comply with study procedures.
- The reason for withdrawal from further study procedures will be recorded in the Case Report Form (CRF). Except in case of complete consent withdrawal, long-term safety data collection will be continued. For all AEs, appropriate follow-up visits or medical care will be arranged, with the agreement of the volunteer, until the AE has resolved,

stabilised or a non-trial related causality has been assigned. Any volunteer who withdrew consent or is withdrawn from further study procedures may be replaced.

- If a volunteer withdraws from the study, blood samples collected before their withdrawal from the trial will be used/stored unless the volunteer specifically requests otherwise. Data from volunteers withdrawn from the study will be included in the analysis of results relating to the study's primary objective.
- In all cases of subject withdrawal, excepting those of complete consent withdrawal, long-term safety data collection will continue as appropriate if subjects have received the inoculum.

9.11 Compensation

Each participant will receive money for transportation to and from study visits and 10kg of sugar per study visit.

10 Clinical and laboratory monitoring

10.1 Monitoring of volunteers

Following the challenge, the volunteers will be monitored for 25 minutes by the study team. They will be provided with a study identification card with study information and contact details for the study team, in case of any concerns or systemic symptoms. The investigator will consider an extra clinical review if the volunteer has any symptoms that are moderate or severe.

10.2 Management of clinical symptoms

N. lactamica is a commensal organism which commonly colonises the nasopharynx but does not cause disease in immunocompetant individuals, with only a few reported cases of clinically significant infection in immunocompromised individuals. We have inoculated over 400 volunteers in the past with wild type *N. lactamica* with no significant safety concerns. In the unlikely event that the lyophilised *N. lactamica* causes disease we would anticipate that this would be either due to involvement of the respiratory tract (which would be signalled by fever and cough) or due to invasion of the bloodstream (which would be signalled by fever and features of sepsis akin to meningococcal disease).

Carriage of *N. lactamica* can be cleared with oral ciprofloxacin or IV ceftriaxone. Ciprofloxacin proved effective at eradicating *N. meningitidis* (18) and should eradicate *N. lactamica* carriage within 24 hours (19). The strain used is fully sensitive to this antibiotic with an exceptionally low MIC on E-testing.

In the event that a study participant develops any clinical symptoms suggestive of infection during the study period they may be reviewed by a member of the study team. If clinically indicated then additional safety bloods will be taken including a blood culture or other investigations as appropriate. If no immediate treatment is indicated then the symptoms will be managed as appropriate for patients with undefined infections. Suspected or confirmed *N.lactamica* disease will be recorded as an AE. The study safety committee will discuss any significant safety concerns.

10.2.1 Phlebotomy

The maximum volume of blood drawn over the study period (60 mls) should not compromise these otherwise healthy volunteers. There may be minor bruising, local tenderness or pre-syncopal symptoms associated with venepuncture, which will not be documented as Adverse Events (AEs) if they occur.

10.2.2 Inoculation with N. lactamica

The inoculation with 0.5 millilitres of *N. lactamica* suspension per nostril can cause some irritation or stinging of the nasal mucosa that will disappear within a few seconds to minutes. Very occasionally, instillation may induce coughing or sneezing.

10.2.3 Throat swab samples

The collection of throat swabs can cause gagging and local discomfort. This discomfort will disappear within a few minutes and will not be recorded as an AE.

11 Laboratory procedures

11.1 Laboratory work

Standard operating procedures for all laboratory work will be followed. Investigators will follow laboratory health and safety standards for working in hazard group 2 pathogens within a containment level 2 laboratory.

11.2 Labelling of samples

Throat swabs and blood samples for immunology will be labelled with a sample ID code containing the participant identification number, sample type and visit number and the date. Safety blood samples will be labelled with the participant identification number, initials and date. Samples will not be labelled with any personal identifiable information.

11.3 Processing of clinical samples

Throat swabs will be delivered to the lab and directly plated within one hour of sampling on to GC agar plates supplemented with X-Gal (5-bromo-4-chloro-3-indolyl-β-D-galactoside). GC + X-gal plates will be incubated for 16-24 hours at 37°C in a CO_2 atmosphere. Presumptive identification of *Neisseria lactamica* will be made based on hydrolysis of X-Gal, which results in dark blue colonies. Successful induction of colonisation will be defined as the culture of at least one colony morphologically consistent with *N. lactamica* on or before day 7 after inoculation.

Four different colonies of presumptive *N. lactamica* (blue colonies) will be subcultured and each subculture will be confirmed as oxidase positive gram negative diplococci. Colonies from each pure subculture will be used for analysis by PCR for confirmation of the presence of the inoculated strain (Y92-1009), see specific SOP for further details. Each subculture will be frozen in TSB plus 15% glycerol for batch analysis for PCR. If the PCR result indicates all subcultures are Y92-1009, two frozen samples will be discarded and one sample will be used for preparation of DNA. If the PCR indicates that the subcultures are not Y92-1009, API will be undertaken to determine the species,

Colonies with morphology typical for *Neisseria spp* on the selective plate from the throat swab that are not blue will be subcultured. Oxidase positive, gram negative diplococci will be considered as putative *Neisseria spp* and further analysed by API. Each *Neisseria* subculture confirmed as a different *Neisseria spp* by API will be frozen at -80ºC in TSB plus 15% glycerol for preparation of DNA using the same methodology as for *N. lactamica*.

The PCR for Y92-1009 will be set up for analysis of the samples at CVD Mali. Boilates will be prepared using standard methodology, and DNA extracted from each subculture using Qiagen kits and shipped to Southampton for further molecular or genomic analysis of presumptive *Neisseria* colonies as appropriate.

Blood samples for immunological assays will be initially processed in the CVD-Mali lab and serum frozen in 2ml aliquots at -80ºC in temperature monitored freezers. One aliquot will remain in Mali, and the remaining aliquots transported to Southampton in dry ice according to IATA guidelines for further analysis. at the University of Southampton and Public Health England, Porton Down, UK. *N. lactamica* specific IgG will be measured using previously described methods. Other potentially relevant immunological or microbiological assays may be performed on stored samples at the discretion of the investigator.

Safety blood samples will be processed in the Centre Rodolphe Merieux laboratory (Bamako, Mali) according to their established laboratory procedures and evaluated using locally approved reference ranges.

12 Assessment of safety

12.1 Safety Evaluation

All relevant safety data will be reviewed by the study safety committee prior to any dose escalation. The local study team will be responsible for providing all the requested information in the required format and approval for dose escalation will be given by the study safety committee. Recruitment and screening may continue while the study safety committee is considering the information provided to them.

12.2 Adverse Event (AE)

An AE is any untoward medical occurrence in a volunteer, including a dosing error, which may occur during or after administration of the inoculum and does not necessarily have a causal relationship with the intervention. An AE can therefore be any unfavourable and unintended sign (including an abnormal laboratory finding), symptom or disease temporally associated with the study intervention, whether or not considered related to the study intervention.

12.3 Adverse Reaction (AR)

An AR is any untoward or unintended response to the inoculum. This means that a causal relationship between the inoculum and an AE is at least a reasonable possibility, i.e., the relationship cannot be ruled out. All cases judged by either the reporting medical investigator or the sponsors as having a reasonable suspected causal relationship to the inoculum (i.e. possibly, probably or definitely related to the inoculum) will qualify as adverse reactions.

12.4 Unexpected Adverse Reaction (UAR)

An adverse reaction, the nature or severity of which is not consistent with the applicable information about the inoculum in the protocol, is considered as an unexpected adverse reaction.

12.5 Serious Adverse Event (SAE)

A serious adverse event is any untoward medical occurrence that results in any of the following outcomes, whether or not considered related to the study intervention.

- Results in death
- Is life threatening
- Results in persistent or significant disability/incapacity
- Requires in-patient hospitalization or prolongation of existing hospitalization
- Is a congenital anomaly/birth defect in the offspring of a study subject
- Is an important medical event that may jeopardize the subject or may require intervention to prevent one of the other outcomes listed above should be considered serious (examples of such treatments are intensive treatment in an emergency room or at home for allergic bronchospasm; blood dyscrasias or convulsions that do not result in hospitalization; or development of drug dependency or drug abuse).

12.6 Serious Adverse Reaction (SAR)

An adverse event (expected or unexpected) that is both serious and, in the opinion of the reporting investigator or sponsors, believed to be possibly, probably or definitely due to the inoculum or any other study treatments, based on the information provided in the protocol.

12.7 Suspected Unexpected Serious Adverse Reactions (SUSARs)

A SUSAR is an SAE that is unexpected and thought to be possibly, probably or definitely related to the inoculum.

12.8 Procedures to be followed in the event of abnormal findings

Abnormal clinical findings from medical history, examination or blood tests, will be assessed as to their clinical significance. If a test is deemed clinically significant, it may be repeated, to ensure it is not a single occurrence. If a test remains clinically significant, the volunteer will be informed and appropriate medical care arranged as appropriate with the permission of the volunteer. Decisions to exclude the volunteer from enrolling in the trial or to withdraw a volunteer from the trial will be at the discretion of the Investigator.

12.9 Foreseeable medical occurrences

The following medical occurrences are foreseeable:

- Local sensation effects in the nose following inoculation.
- Local bruises following venepuncture

12.10 Severity grading of clinical adverse events

The severity of clinical adverse events will be assessed according to the scales in table 12.1. Safety bloods will be obtained prior to inoculation and on day 7 and will be assessed according to tables 12.2 and 12.3.

**GRADE 0**	None
**GRADE 1**	Mild: Transient or mild discomfort (< 48 hours); no medical intervention/therapy required
**GRADE 2**	Moderate: Mild to moderate limitation in activity - some assistance may be needed; no or minimal medical intervention/therapy required
**GRADE 3**	Severe: Marked limitation in activity, some assistance usually required; medical intervention/therapy required, hospitalisation possible

Table 12.1: Severity grading criterion for AEs.

Biochemistry		Mild (Grade 1)	Moderate (Grade 2)	Severe (Grade 3)	Potentially Life Threatening (Grade 4)
Creatinine (µmol/l)		132-150	151-176	177-221	> 221 or requires dialysis
ALT (IU/L)		50-112	113-225	226-450	> 450

Table 12.2 – Grading of laboratory adverse events - Biochemistry

Hematology		Mild (Grade 1)	Moderate (Grade 2)	Severe (Grade 3)	Potentially Life Threatening (Grade 4)
Hemoglobine (gm/dL)	Female – absolute value	10.0 – 10.9	9.0 – 9.9	8.0 – 8.9	< 8.0
	Female – decrease from baseline value		1.6 – 2.0	2.1 – 5.0	> 5.0
	Male – absolute value	11.5 – 12.4	10.5 – 11.4	9.5 – 10.4	< 9.5
	Male – decrease from baseline value		1.6 – 2.0	2.1 – 5.0	> 5.0
WBC (10^3 / µL)	Increase	11.6 – 15.0	15.1 – 20.0	20.1 – 25.0	> 25.0
	Decrease	2.35 – 3.20	1.50-2.34	1.00 – 1.49	< 1.0
Lymphocytes (10^3 / µL)	Decrease	0.75 – 0.99	0.50 – 0.74	0.49 – 0.25	< 0.25
Neutrophils (10^3 / µL)	Decrease	1.19 – 0.85	0.84 – 0.50	0.49 – 0.40	< 40
Eosinophils ((10^3 / µL)		0.50 – 1.50	1.51 – 5.00	> 5.00	Hypereosinophilic
Platelets (10^3 / µL)	Decrease	125 – 135	124 – 100	99 – 25	< 25

Table 12.3 – Grading of laboratory adverse events - Hematology

* The laboratory values provided in the tables serve as guidelines and are dependent upon institutional normal parameters. Institutional normal reference ranges should be provided to demonstrate that they are appropriate.

**“ULN” is the upper limit of the normal range

12.11 Causality assessment

For each AE, an assessment of the relationship of the AE to the study intervention(s) will be undertaken. The relationship of the adverse event with the study procedures will be categorised as unrelated, unlikely to be related, possibly related, probably related or definitely related (Table 12.4). An intervention-related AE refers to an AE for which there is a possible, probable or definite relationship to the study intervention. The investigator will use clinical judgment to determine the relationship. Alternative causes of the AE, such as the natural history of pre-existing medical conditions, concomitant therapy, other risk factors and the temporal relationship of the event to the challenge will be considered and investigated.

0	**No Relationship**	No temporal relationship to the challenge ***and***<br>Alternate aetiology (clinical state, environmental or other interventions); ***and***<br>Does not follow known pattern of response to *N. lactamica*
1	**Unlikely**	Unlikely temporal relationship to the challenge ***and***<br>Alternate aetiology likely (clinical state, environmental or other interventions) ***and***<br>Does not follow known pattern of response to *N. lactamica*
2	**Possible**	Reasonable temporal relationship to the challenge; ***or***<br>Event not readily produced by clinical state, environmental or other interventions; ***or***<br>Follows expected pattern of response to *N. lactamica*
3	**Probable**	Reasonable temporal relationship to the challenge; ***and***<br>Event not readily produced by clinical state, environment, or other interventions ***or***<br>Follows expected pattern of response to *N. lactamica*
4	**Definite**	Reasonable temporal relationship to the challenge; ***and***<br>Event not readily produced by clinical state, environment, or other interventions; ***and***<br>Follows expected pattern of response to *N. lactamica*

Table 12.4: Guidelines for assessing the relationship of an AE to inoculation with *N. lactamica*

12.12 Reporting procedures for AEs

If an adverse event occurs it will first be reported to the study doctor, who will investigate and document it in the CRF. If an AE is considered to be related and serious, a report will be written and sent to the study safety committee within 24 hours of PI being aware. The Sponsor/PI will be informed if the SAE is assessed by the safety committee as having potential to cause harm to the volunteer or subsequent volunteers. The Sponsor/PI will then decide the action to be taken. All SAEs will be reported to the local Ethics Committee within 3 business days of the PI becoming aware. All related and serious AEs and SUSARs will be reported to the UMB IRB within 5 business days of the PI becoming aware.

All AEs will be summarized and reviewed by the study safety committee prior to dose change. Though there are no specific halting criteria, the study safety committee will consider all available safety information prior to proceeding to the next dose. The Chair or his designee will provide the final go-ahead to the next dose level based on discussions with the other committee members.

AEs that result in a volunteer's withdrawal from the study or that are present at the end of the study will be followed up (if volunteer consents to this) until a satisfactory resolution or stabilisation occurs, or until a non-study related causality is assigned.

13 Acceptability and feasibility assessment

13.1 Overview

Concurrent with commencing inoculations in these volunteers, a qualitative acceptability and feasibility assessment will be performed. Members of the local community will be invited to participate in focus groups and/or semi-structured interviews facilitated by CVD Mali social and behavioural scientists. Discussants will include a broad range of individuals from representative locations. These tools will be developed in the appropriate language and used to investigate levels of knowledge about meningitis and attitudes towards nasal inoculation and other study procedures within the community. Verbal consent will be gained prior to participation.

13.2 Research questions

This assessment will explore the following questions in order to evaluate the acceptability and feasibility of performing nasal inoculation with lyoNlac in healthy Malian volunteers.

1. What knowledge do people have about meningitis in the community?
2. What is the attitude towards interventions for meningitis in the community?
3. Are there any products which are used nasally in the local community?
4. What products are used nasally? Are these considered to be generally acceptable or risky within this community?
5. What might the constraints be for the nasal inoculation of healthy adult volunteers with lyoNlac? If this route of administration is used for products not generally approved of in the community, how would the community view the use of this route of administration of a potential vaccine?
6. What might the enabling factors be for the nasal inoculation of healthy adult volunteers with lyoNlac?
7. Is it acceptable to the local community to nasally inoculate healthy adult volunteers with lyoNlac as part of a study seeking to improve protection from meningitis in the future?
8. Who are the stakeholders to facilitate the implementation of this study?
9. What are the perspectives of different stakeholders about the acceptability of this study?
10. What are the requirements for the implementation of this study, at different levels but in particular at the community level?
11. What are the recommendations for the implementation of this study?

13.3 Geographical location

The acceptability and feasibility assessment will be conducted in Niamakoro and Sebeninkoro within the district of Bamako. These two areas have been selected as they are distinct from, but similar to, the recruitment areas for the clinical study. They are urban areas for which demographic data is available, A notable difference from the clinical study recruitment areas is that one area is on the right side of the Niger River.

13.4 Discussant characteristics

The discussants will include:

- Community leaders
- Religious leaders (traditional, Muslim, Christian)
- Health service providers (modern and traditional)
- Adults (female and male)

13.5 Consent methods

Consent will be obtained verbally while using an IRB- and Ethics Committee-approved script.

13.6 Assessment methods

The acceptability and feasibility assessment will be conducted using:

- In-depth interview of key informants (community leaders, religious leaders, health service providers)
- Focus group discussions with adults representative of potential study participants
- Observations (events related to the nasal use of products, types of products)

13.7 Assessment instruments

The following instruments will be IRB- and Ethics Committee- approved prior to use:

- Guide for in-depth interview
- Guide for Focus group discussions
- Guide for observations

14 Statistical analysis

14.1 Sample size

Volunteers will be challenged in cohorts of up to 5 volunteers, with a dose ranging strategy continuing until 10 volunteers have been colonised by day 7 with the standard inoculum (the dose inducing colonisation in at least 70% or a maximum dose of 10^7 CFU). Ten carriers will be sufficient to confirm both the genomic stability of the inoculated strain and its immunogenicity.

The statistical rationale relating to the sample required for the serological response (a secondary endpoint) is that using the standard deviations of serological response to wild

type *N. lactamica* derived in our first experimental human challenge study published (Evans C et al 2011) which gave SDs on a log-10 scale of 0.26 for Serum total IgG. Using the SD of 0.26 we will be able to confirm a 4-fold rise with 10 carriers of Nlac with 90% power using analysis of variance.

Starting at a dose of 10^5 CFU, escalating by up to 1 $\log_{10}$ and with a maximum dose of 10^7 CFU, we estimate that 25-100 volunteers will be required to identify the standard inoculum and colonise 10 volunteers with this dose.

14.2 Statistical analysis

Statistical analysis will be performed using GraphPad Prismsoftware, by the statistical unit of the NIHR Southampton Biomedical Research Centre. Serological data will be analyzed using 1-way analysis of variance. Log-transformed data will be used to construct areas under the curve for comparison of immune responses over the study period.

15 Quality control and quality assurance

15.1 Investigator procedures

Approved site-specific SOPs will be used at all clinical and laboratory sites.

15.2 Quality Control

As a first step to establishing the reliability of the data, all case report forms will undergo a quality control check by members of the supervising staff. This verification will be completed as soon as possible after the completion of the form to ensure the prompt resolution of any omissions or inconsistencies reported on the case report forms. Any necessary corrections will be performed according to standard procedure.

15.3 Quality Assurance

15.3.1 Internal Monitoring

The CVD-Mali Local Regulatory Affairs Specialist, in conjunction with the CVD-Baltimore International Regulatory Affairs Specialist, will be responsible for conducting periodic audits of all study related documentations, including the regulatory file and a selected number of case report forms and consent forms. Their findings will be reported to the CI and PI.

15.3.2 External Monitoring

Clinipharm is an independent external monitoring agency that will be responsible for providing regular monitoring of all study documentation according to the monitoring plan designed with the sponsor.

16 Regulatory and Ethical Requirements

The study will be conducted according to most recent version of ICH Good Clinical Practice guidelines, the Declaration of Helsinki, and applicable laws and regulations of Mali.

16.1 Institutional Review Boards and Ethics Committees

The protocol and associated consent forms will be reviewed and approved by the University of Maryland Human Research and Protections Office and the Ethics Committee of the Faculté de Medecine, Pharmacie et Odonto-Stomatologie in Bamako, Mali prior to study start. All communications regarding any future amendments and annual approvals will be filed along with the original study approval in the regulatory files.

16.2 Local Community Permission

As described above, it is customary to obtain the permission of the community prior to initiating study activities. These permissions will be documented and stored in the regulatory.

16.3 Study amendments

No amendments to this protocol will be made without consultation with, and agreement of, the Sponsor. Any amendments to the trial that appear necessary during the course of the trial must be discussed by the investigator and sponsor concurrently. If agreement is reached concerning the need for an amendment, it will be produced in writing by the chief investigator and will be made a formal part of the protocol following ethical and regulatory approval.

An administrative change to the protocol is one that modifies administrative and logistical aspects of a protocol but does not affect the subjects' safety, the objectives of the trial and its progress. An administrative change does not require ethical committee or regulatory approval.

Any amendments to study documents will follow established regulatory requirements.

The investigator is responsible for ensuring that changes to an approved study, during the period for which regulatory and ethical committee(s) approval has already been given, are not initiated without regulatory and ethical committee(s)' review and approval except to eliminate apparent immediate hazards to the subject.

16.4 Protocol deviation

Any deviations from the protocol will be documented in a protocol deviation form and filed in the site trial master file.

16.5 Serious breaches

A serious breach is defined as "A breach of GCP or the trial protocol which is likely to affect to a significant degree – the safety or physical or mental integrity of the subjects of the trial; or the scientific value of the trial."

In the event that a serious breach is suspected the study safety committee and Sponsor will be informed as soon as possible and in turn will notify the ethical committee within 7 days.

16.6 Study completion/termination

The trial will be considered complete upon the last volunteer/last visit at the site. The data will be sent to the sponsor in the timeframe specified in the Clinical Trial Agreement.

The study may be terminated early at the discretion of the Chief Investigator, Sponsor or Study Safety Committee if there are safety concerns, concerns about compliance with GCP

or other appropriate regulations, poor recruitment or new information becomes available which has an impact on the scientific validity or safety of the trial.

16.7 Exploitation and dissemination

The investigators will be involved in reviewing drafts of the manuscripts, abstracts, press releases and any other publications arising from the study. Findings will be published in peer reviewed journals as soon as possible, even where results prove negative. The authors will acknowledge that the study has been funded by the National Institute for Health Research, Mucosal Pathogens Research Unit (NIHR MPRU), University College London. The results of the study will be disseminated at relevant international scientific meetings.

17 Data handling and record keeping

17.1 Data handling

The local study team will enter all study data into the volunteers' case report forms (CRFs), which will be in a paper format. This includes safety data, laboratory data (both clinical and immunological) and outcome data. Data will then be entered and stored in password protected computers.

17.2 Volunteer confidentiality

All data will be link anonymised; volunteer data will be identified by a unique participant number in the case report form. Separate confidential files containing identifiable information will be stored in secured locations. Only the sponsor representative, investigators, the clinical monitor, the ethical committee(s) and the regulatory authorities will have access to the records. All case report forms will be stored in locked cabinets and any computers used to collect or store study data will be password protected.

Given that study personnel may visit the participant's home it is possible that in a given household, an individual's participation in this trial could become known. Nonetheless, study personnel will make every attempt to maintain the privacy of individuals who are participating within the household. For example, testing results will only be discussed with the concerned participants and private discussions and assessments will be performed apart from other household members.

18 Financing

The study will be supported by funding from the National Institute for Health Research, Health Protection Research Unit (NIHR HPRU) in Mucosal Immunology which is based at University College London.

19 References

1. Bidmos FA, Neal KR, Oldfield NJ, Turner DP, Ala'Aldeen DA, Bayliss CD. Persistence, replacement, and rapid clonal expansion of meningococcal carriage isolates in a 2008 university student cohort. Journal of clinical microbiology. 2011;49(2):506-12.
2. Denning DW, Gill SS. Neisseria lactamica meningitis following skull trauma. Reviews of infectious diseases. 1991;13(2):216-8.
3. Brown NM, Ragge NK, Speller DC. Septicaemia due to Neisseria lactamica--initial confusion with Neisseria meningitidis. The Journal of infection. 1987;15(3):243-5.
4. Laver JR, Hughes SE, Read RC. Neisserial Molecular Adaptations to the Nasopharyngeal Niche. Adv Microb Physiol. 2015;66:323-55.
5. Cartwright KA, Stuart JM, Jones DM, Noah ND. The Stonehouse survey: nasopharyngeal carriage of meningococci and Neisseria lactamica. Epidemiol Infect. 1987;99(3):591-601.
6. Trotter CL, Gay NJ, Edmunds WJ. The natural history of meningococcal carriage and disease. Epidemiol Infect. 2006;134(3):556-66.
7. Lauer BA, Fisher CE. Neisseria lactamica meningitis. American journal of diseases of children (1960). 1976;130(2):198-9.
8. Maiden MC, Ibarz-Pavón AB, Urwin R, Gray SJ, Andrews NJ, Clarke SC, et al. Impact of meningococcal serogroup C conjugate vaccines on carriage and herd immunity. The Journal of infectious diseases. 2008;197(5):737-43.
9. Read RC, Baxter D, Chadwick DR, Faust SN, Finn A, Gordon SB, et al. Effect of a quadrivalent meningococcal ACWY glycoconjugate or a serogroup B meningococcal vaccine on meningococcal carriage: an observer-blind, phase 3 randomised clinical trial. Lancet. 2014;384(9960):2123-31.
10. Agier L, Martiny N, Thiongane O, Mueller JE, Paireau J, Watkins ER, et al. Towards understanding the epidemiology of Neisseria meningitidis in the African meningitis belt: a multi-disciplinary overview. International journal of infectious diseases : IJID : official publication of the International Society for Infectious Diseases. 2017;54:103-12.
11. Mueller JE, Gessner BD. A hypothetical explanatory model for meningococcal meningitis in the African meningitis belt. International journal of infectious diseases : IJID : official publication of the International Society for Infectious Diseases. 2010;14(7):e553-9.
12. Koutangni T, Boubacar Mainassara H, Mueller JE. Incidence, carriage and case-carrier ratios for meningococcal meningitis in the African meningitis belt: a systematic review and meta-analysis. PloS one. 2015;10(2):e0116725.
13. World Health O. Epidemic meningitis control in countries of the African meningitis belt, 2017. Weekly epidemiological record. 2018.
14. WHO Guidelines Approved by the Guidelines Review Committee. Managing Meningitis Epidemics in Africa: A Quick Reference Guide for Health Authorities and Health-Care Workers. Geneva: World Health Organization

Copyright (c) World Health Organization 2010. Revised 2015.; 2015.
15. Hassan A, Mustapha GU, Lawal BB, Na'uzo AM, Ismail R, Womi-Eteng Oboma E, et al. Time delays in the response to the Neisseria meningitidis serogroup C outbreak in Nigeria - 2017. PloS one. 2018;13(6):e0199257.
16. Lewis R, Nathan N, Diarra L, Belanger F, Paquet C. Timely detection of meningococcal meningitis epidemics in Africa. Lancet (London, England). 2001;358(9278):287-93.
17. Evans CM, Pratt CB, Matheson M, Vaughan TE, Findlow J, Borrow R, et al. Nasopharyngeal colonization by Neisseria lactamica and induction of protective immunity against Neisseria meningitidis. Clinical infectious diseases : an official publication of the Infectious Diseases Society of America. 2011;52(1):70-7.
18. Deasy AM, Guccione E, Dale AP, Andrews N, Evans CM, Bennett JS, et al. Nasal Inoculation of the Commensal Neisseria lactamica Inhibits Carriage of Neisseria meningitidis

by Young Adults: A Controlled Human Infection Study. Clinical infectious diseases : an official publication of the Infectious Diseases Society of America. 2015;60(10):1512-20.
19. Fraser A, Gafter-Gvili A, Paul M, Leibovici L. Antibiotics for preventing meningococcal infections. The Cochrane database of systematic reviews. 2006(4):CD004785.
